# Hydrogen Bonding within Dynamic ^19^F‑Tagged Oligothiourea Foldamers in Solution and in Membranes

**DOI:** 10.1021/jacs.6c05314

**Published:** 2026-06-17

**Authors:** Lucia Trevisan, Kathryn S. Foster, Jonathan Clayden

**Affiliations:** School of Chemistry, 1980 University of Bristol, Bristol BS8 1TS, U.K.

## Abstract

Molecular conformation plays an integral role in biological signal transduction, with several classes of biomolecules using conformational changes to translate local inputs into spatially remote outputs. This natural communication mechanism has inspired the development of dynamic foldamers with the ability to use conformational changes as a communication mechanism. These foldamers include ethylene-bridged hydrogen-bonded oligo­(thio)­urea foldamers with reversible hydrogen-bond polarity. Using *p*-fluorobenzyl capping groups, we show that ^1^H and ^19^F variable-temperature and 2D NMR reveal details of the directionality and resilience of the intramolecular hydrogen-bond network of these dynamic foldamers at a range of temperatures. Crucially, ^19^F VT NMR allowed the dynamic behavior of the foldamers to be studied in micelles and bicelles, into which they were readily incorporated. The intactness of the network was investigated both in solution and in membrane-mimetic environments by ^19^F NMR spectroscopy for foldamers containing between two and five thioureas. With longer foldamers, the high resolution and sensitivity of the fluorine probes allowed intermediates in the global hydrogen-bond directionality reversal process to be identified. These findings reveal subtleties in the conformational preferences of hydrogen-bonded foldamers that have relevance to their use as communication devices both in solution and in membranes separating compartmentalized environments.

## Graphical abstract

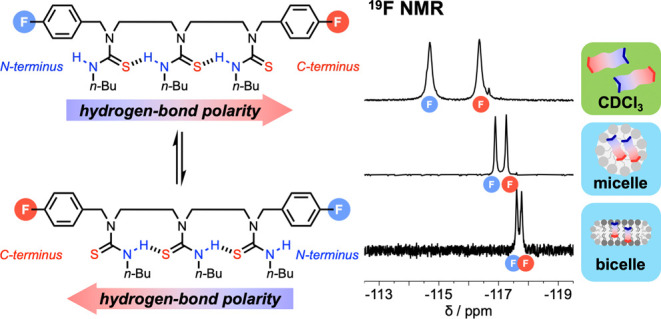

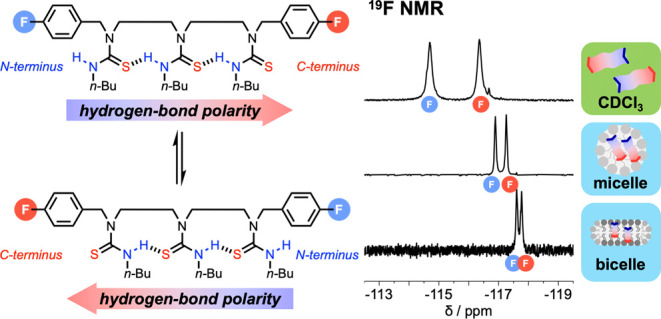

## Introduction

Inspired by nature’s use of conformational switching to relay signals through molecular structures, for example, in receptor kinases^1^ and G-protein-coupled receptors,^2^ chemists have developed classes of extended molecules with switchable conformations that are maintained by coherent networks of hydrogen bonds.^3–5^ We have reported several families of such “dynamic foldamers”^6^ which are capable of communicating information, adopting multiple well-defined conformations while retaining overall structural definition. Global inversion of helical screw sense in oligomeric structures built from α-aminoisobutyric acid (Aib) residues (Figure 1a) has been used as a conformational relay mechanism, translating input signals such as a chiral acid,^3^ light,^7–9^ or a chiral ligand,^10^ to spatially remote sites, detected through fluorescence emission,^10^ NMR spectroscopy,^8,10^ or a change in selectivity.^9^ With the aim of using these dynamic foldamers as mimics of transmembrane proteins, Aib foldamers have been incorporated into the membrane of vesicles^8,11^ or micelles.^12^ ^19^F NMR has proved especially useful for monitoring conformational switching in foldamer systems, both in solution and in the membrane phase,^8,11,12^ as the chemical shifts of ^19^F labels are very sensitive to changes in environment in both small molecules^13,14^ and proteins.^15–18^

**Figure 1:**
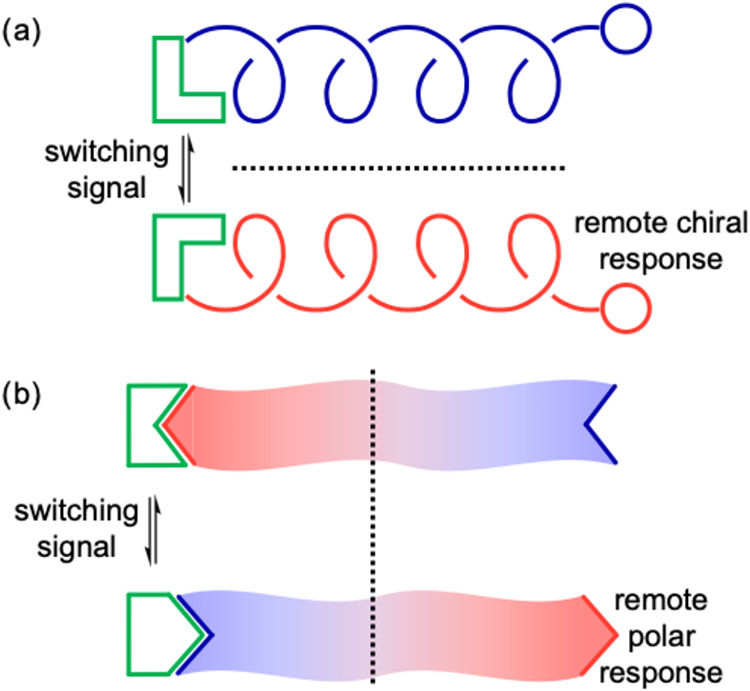
Molecular communication of information through (a) chirality switching of a helical foldamer and (b) hydrogen bond polarity reversal of an oligo­(thio)­urea foldamer.

Switching of global hydrogen bond directionality has more recently also been demonstrated as a communication mechanism, initially in hydrogen-bonded oligourea helices built from cyclohexane-1,2-diamine units,^19,20^ and more recently in achiral ethylene-bridged oligoureas and thioureas (Figure 1b).^4,21,22^ The directionality of the global intramolecular hydrogen bonding network in these dynamic foldamers may be modulated using input signals such as pH^4^ or anion complexation,^21^ and monitored by output signals such as fluorescence,^4^ capture and release of a ligand,^22^ or activation of nucleophilicity by induced tautomerism at a remote site.^21^ The oligo­(thio)­urea “ribbon” has lower conformational rigidity than the oligo-Aib helix, making it a more versatile scaffold, and achiral communication mechanisms based (like the genetic code) on hydrogen bond directionality, rather than chirality, have greater potential for use in homochiral biological environments. However, further applications of the ethylene-bridged oligoureas are limited by the lack of detailed information on the structure and dynamics of their hydrogen bonding networks, whether in solution or in the membrane phase.

In this paper, we show for the first time that oligothiourea foldamers can insert into membrane mimics. We demonstrate the use of ^19^F NMR as a means of probing the conformational dynamics of these oligomers in solution, and we show that ^19^F NMR can also reveal details of conformational preference in the membrane phase. These techniques allow a comparison of the conformational dynamics and hydrogen-bond resilience of the same molecules both in solution and in membrane mimics.

## Approach

The wealth of structural information provided by NMR spectroscopy at a range of temperatures in solution has provided a detailed picture of the dynamic behavior of poly-Aib foldamers^8–12^ and has likewise illuminated some important aspects of the conformational kinetics of oligourea^4^ scaffolds. However, the application of this spectroscopic technique to study molecules embedded in colloids is constrained by several factors: the size of the particles, which often results in very broad signals due to the slow tumbling rate, the high concentration of lipid, detergent, or buffer compared to the molecule of interest needed to obtain a stable suspension, and the limited temperature range that can be explored. In this study, we reveal ways to overcome these challenges, comparing the dynamic behavior of oligothiourea foldamers in solution and in different membrane-mimetic environments.

The chemical shift of the ^19^F NMR nucleus is highly sensitive to its environment, the ^19^F isotope is present at 100% natural abundance, and ^19^F NMR spectra are uncluttered by signals from solvent or nonfluorinated membrane components such as lipids or detergents.^23^ The dispersion of the ^19^F NMR chemical shift range is 100-fold larger than that of ^1^H NMR due to the paramagnetic shielding from the fluorine lone pairs,^23^ which allows ^19^F NMR to amplify subtle differences in chemical environments at the N- and C-terminus of a foldamer: fluorine probes adjacent to hydrogen-bond donors and hydrogen-bond acceptors display distinct chemical shifts. We therefore envisaged that fluorine probes within ethylene-bridged oligothioureas would reveal detailed kinetic and thermodynamic information about the structure and hydrogen bond networks of these foldamers, both in solution and in the membrane phase. To do this, we synthesized a series of ethylene-bridged oligothiourea foldamers **2**–**5**, along with the reference monomeric thiourea **1** (Figure 2a), capped by terminal 4-fluorobenzyl groups to provide ^19^F NMR probes. *n*-Butyl side chains were chosen to avoid the complication of π-π stacking interactions between the terminal thioureas and these capping groups.

**Figure 2:**
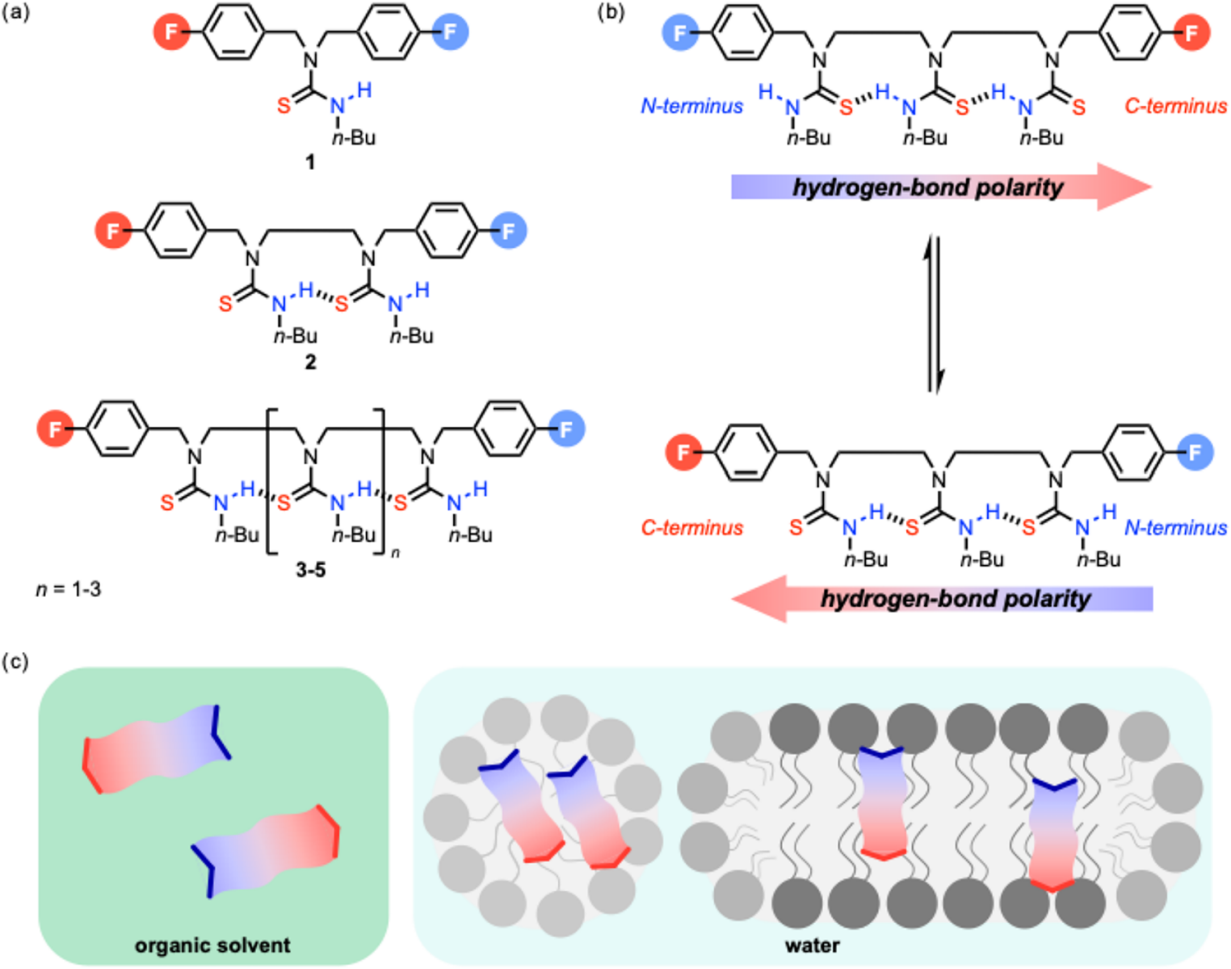
(a) Hydrogen-bonded oligourea foldamers **2**–**5** and reference monomer **1** with ^19^F-tagged capping groups (b) to study the foldamers’ dynamics (c) in organic solvents, in micelles and bicelles.

In comparison with previous oligourea foldamers,^4,21,22^ the intramolecular hydrogen-bonded networks of thioureas in **2**–**5** are expected to exhibit higher barriers to rotation Δ*G* ^‡^ about the C–N bonds^13,22^ that may be increased even more significantly by the presence of a hydrogen-bond acceptor.^24^ The detailed study of conformational processes by dynamic NMR requires signals relating to different conformers to undergo intermediate or slow exchange on the NMR time scale at accessible temperatures, and hydrogen-bonded conformers with opposite directionalities (Figure 2b) fulfill this requirement as they will be in slow exchange above 273 K. Flexing of the scaffold backbone between anti and gauche conformers^4^ and rotation of side chains also occur, but these are invariably fast on the NMR time scale. The slower conformational interconversion of thiourea C–N bonds thus allows dynamic NMR to filter out the conformational dynamics of the hydrogen-bond network, both in solution and in colloidal systems dispersed in water, uncluttered by inconsequential rotation of the aliphatic chains. Throughout this paper, we therefore use the term “conformer” to refer exclusively to the ensemble of conformers associated with a single thiourea C–N rotamer. With these oligothiourea foldamers **2**–**5** in hand, we set out to quantify the structure and dynamics of their intramolecular hydrogen bond networks by NMR spectroscopy, both in organic solvents (deuterated chloroform and DCM) and in colloidal particles, specifically micelles and bicelles (Figure 2c).

## Results and Discussion

### Synthesis of the Oligomers

Compounds **1**–**5** were synthesized by the route shown in Figure 3. The synthesis of **1** and **2** employed the reductive amination of a commercial amine or diamine to give an oligo­(ethylenediamine) backbone carrying two fluorine probes. Functionalization with *n*-butyl isothiocyanate installed the thiourea side chains. The synthesis of **3**, **4**, and **5** first necessitated protection of the terminal NH_2_ sites of the commercial polyamine prior to installation of the central *n*-Bu thiourea side chains. Subsequent deprotection, reductive amination, and *n*-Bu thiourea side-chain installation then provided access to the full homologous series of **1**–**5**.

**Figure 3:**
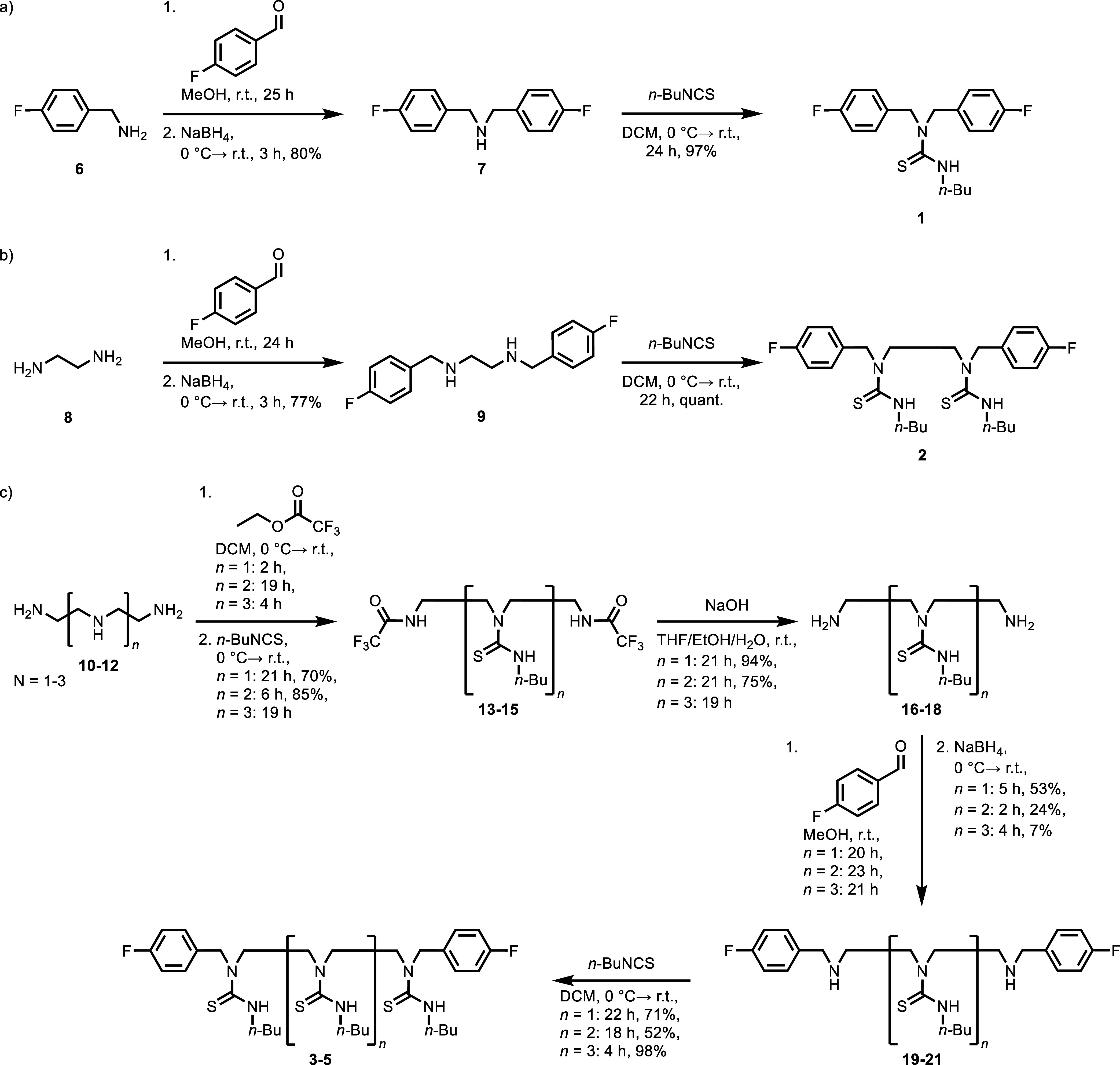
Synthesis of (a) reference monomer **1** and oligothiourea foldamers (b) **2** and (c) **3**-**5**.

### Dynamic Conformation of Oligothiourea Foldamers in Solution

The conformational dynamics of the intramolecular hydrogen-bonding network in reference compound **1** and foldamers **2**–**5** were first investigated in solution, using both ^1^H and ^19^F NMR spectroscopy (Figure 4). Reference compound **1** contains no intramolecular hydrogen bond, and thus, in the ^1^H NMR spectrum, it exhibited only one NHA (see labeling in Figure 4a) peak at 5.41 ppm at 298 K (Figure 4c). The presence of a single intramolecular hydrogen bond in compound **2** resulted in a broad signal at 6.45 ppm for the two NH signals (NHA and NHA′) in the ^1^H spectrum at 298 K (Figure 4c). Cooling the sample to 248 K led to their decoalescence into two separate peaks at 5.33 and 7.94 ppm (Figure 4c). Given the difference in chemical shift of 2.6 ppm, the upfield peak corresponds to the non-hydrogen-bonded NHA and the downfield signal to the hydrogen-bonded NHA′.^25,26^ Longer foldamers **3**-**5** all exhibit a single NH peak at an upfield chemical shift (5.35 ppm for **3**, 5.39 ppm for **4**, 5.37 ppm for **5**) and a series of more downfield NH peaks (7.75 ppm for **3**; 7.87 ppm, 7.98 ppm for **4**; 7.78 ppm, 7.98 ppm, 8.10 ppm, 8.20 ppm for **5**) at 298 K (Figure 4c). The upfield signals each correspond to the terminal NHA that is not involved in the intramolecular hydrogen-bonded network, and their chemical shifts are comparable to the (evidently non-hydrogen-bonded) NHA peak of reference compound **1**, at 5.41 ppm. For example, in the rotating frame overhauser enhancement spectroscopy (ROESY) experiment for **3** (Figure 4b) only, the NHA peak exhibits a ROE cross-peak with the benzylic H5 signals (green highlight). Thus, the series of downfield peaks, which are deshielded by at least 2.4 ppm, belong to the intramolecularly hydrogen-bonded NHB and NHA′ signals.^25,26^

**Figure 4:**
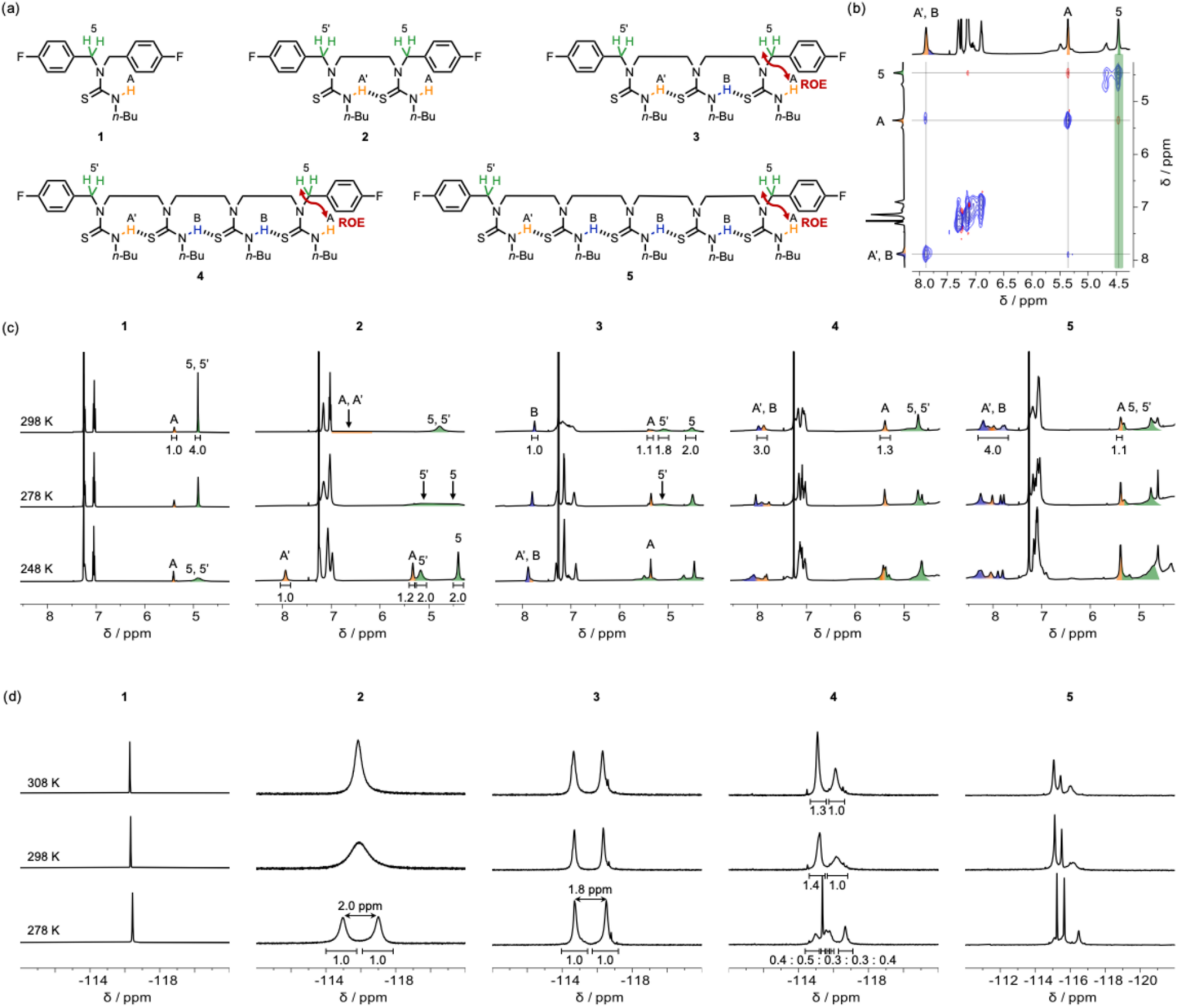
NMR study of oligothioureas foldamers **1**–**5** conformation in solution. (a) Molecular structures of **1**–**5** with NMR labels. (b) 500 MHz ^1^H–^1^H ROESY spectrum of **3** (8 mM) in CDCl_3_ at 248 K. A green highlight shows that H5 has an ROE cross-peak with NHA, and no ROE cross-peaks with NHA′ or NHB. (c) 500 MHz ^1^H VT NMR spectra of **1**–**5** (8 mM) at 298, 278 and 248 K in CDCl_3_. (d) 565 MHz ^19^F VT NMR spectra of **1**–**5** (8 mM) at 308, 298 and 278 K in CDCl_3_.

The partitioning of the NH groups in each oligomer **2**–**5** indicates that, typically, all of them except for one (i.e., NHA) are in hydrogen-bonded environments, suggesting a single coherent hydrogen-bonded chain of thiourea groups for all these compounds. The decoalescence of the benzylic protons H5 and H5′ of the capping groups for **2**–**5** at around 4–5 ppm gives at least two peaks with different line shapes at 248 K that overlap with neighboring signals (Figure 4c). However, due to its higher sensitivity and more disperse frequency range, ^19^F NMR allowed the analysis of this complex conformational behavior, with multiple fluorine environments evident in the longer oligomers (Figure 4d). In the ^19^F NMR spectrum, monomer **1** shows a single sharp peak arising from two NH environments in fast exchange down to 278 K, the lower C–N rotational barrier being due to the absence of an intramolecular hydrogen bond.^4^ Moving to dimer **2**, significant ^19^F NMR peak broadening implies a shift to intermediate exchange at 298 K, and further toward slow exchange at 278 K with two equally integrating peaks (Figure 4d), presumably because the dimer possesses one intramolecular hydrogen bond which must be broken and then reformed in order to exchange the environments of the two fluorine probes. Upon introduction of a second intramolecular hydrogen bond, in trimer **3**, two distinct ^19^F NMR peaks appear, indicating that the fluorine probes in the trimer are in slow exchange even up to 308 K. The two peaks integrate equally (with no change in NMR spectra upon cooling to 278 K), which is consistent with **3** populating only the pair of degenerate conformers shown in Figure 5a, in which the thiourea units are linked by a coherent chain of hydrogen bonds. It is likely that the clear separation into two well-resolved peaks here is also facilitated by cooperativity effects, which enhance the difference in chemical shift of the two fluorine probes relative to the dimer by increasing the electron richness of the terminal thiourea C=S unit and the electron deficiency of the terminal thiourea N–H unit.^27^

**Figure 5:**
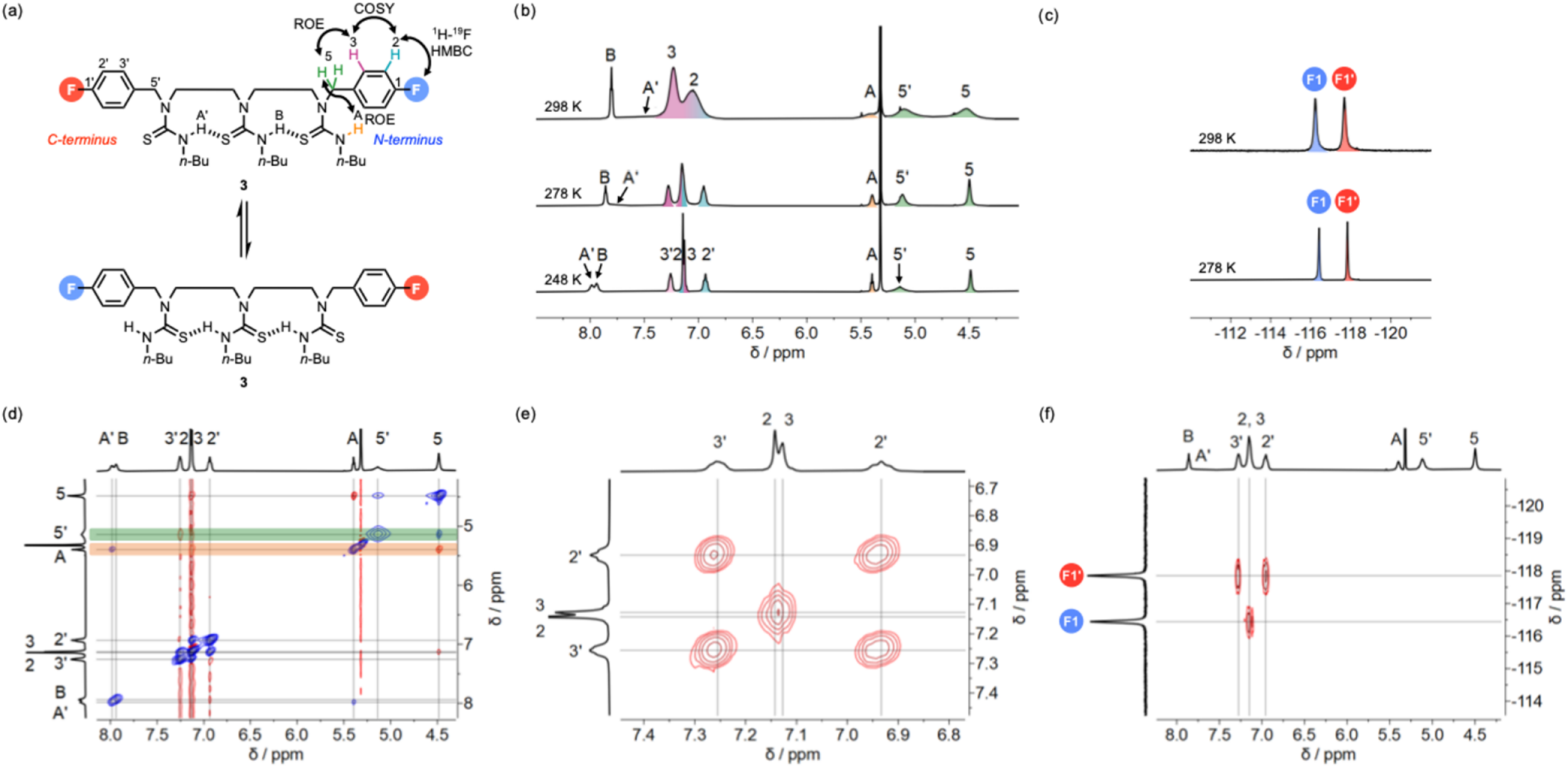
Analysis of the conformation of **3** in solution by NMR. (a) Molecular structure of **3** and NMR labels. (b) 500 MHz ^1^H VT NMR of **3** (10 mM) in CD_2_Cl_2_ at 298, 278 and 248 K. (c) 565 MHz ^19^F VT NMR of **3** (10 mM) in CD_2_Cl_2_ at 298 and 278 K. (d) 500 MHz ^1^H–^1^H ROESY of **3** (10 mM) in CD_2_Cl_2_ at 248 K. An orange highlight shows that NHA has an exchange cross-peak with NHA′, an ROE cross-peak with H5 and no ROE cross-peak with H5′. A green highlight shows that H5′ has an ROE cross-peak with H3′ and an exchange cross-peak with H5. (e) 500 MHz ^1^H–^1^H COSY of **3** (10 mM) in CD_2_Cl_2_ at 248 K. (f) 400 MHz ^1^H–^19^F HMBC of **3** (10 mM) in CD_2_Cl_2_ at 278 K.

The situation changes significantly upon moving to tetramer **4**, which shows two ^19^F NMR peaks with different line widths and integrals at 298 K. On cooling of the sample to 278 K, these two peaks decoalesce to reveal five different peaks, with characteristic line shapes (one sharp, four broad), indicating the population of multiple conformers. The ^19^F NMR spectrum for pentamer **5** increases in complexity even further, showing at least three ^19^F NMR signals at 308 K.

The intricate NMR spectra of **3**-**5** were assigned by means of detailed ^1^H and ^19^F VT NMR analysis, using the more uniform conformational population of trimer **3** and the better resolution in CD_2_Cl_2_ compared to CDCl_3_ to validate methods for assigning NMR signals that were then applied to tetramer **4**. We were able to assign the two ^19^F NMR signals to the N-terminal and the C-terminal environments of trimer **3** by a series of homonuclear and heteronuclear correlations (Figure 5a) in the ^1^H and ^19^F NMR spectra. At 298 K (Figure 5b), the ^1^H NMR spectrum shows three NH peaks: at 5.41 ppm for NHA (in orange, clearly non-hydrogen bonded, and hence located at the N-terminus), at 7.51 ppm (broad peak) and 7.80 ppm for NHA′ and NHB, respectively (both hydrogen bonded). The benzylic protons appear as two equally integrating signals at 5.14 ppm for H5′ and 4.49 ppm for H5. The assignment of the signal at 4.49 ppm to H5, i.e., the N-terminal benzylic position, was made through a ROESY correlation with the (non-hydrogen bonded) NHA, a correlation that was absent for the C-terminal H5′ signal at 5.14 ppm (orange highlight in Figure 5d). The two ^19^F NMR signals (Figure 5c) at −116.44 ppm (N-terminal F1, blue) and at −117.85 ppm (C-terminal F1′, red) were likewise unequivocally assigned through a series of 2D NMR correlations as shown in Figure 4a: ROEs between N-terminal H5 and the signals H2, H3 of its associated aromatic ring (which are resolved by decoalescence at 248 K – see ^1^H in Figure 5b, green highlight in ROESY in Figure 5d and COSY in Figure 5e), and then a long-range heteronuclear correlation between the *ortho* protons H2 and F1 in blue (and similarly between H2′ and F1′ in red – see Figure 5f). Together, these experiments allow us to draw the important conclusion that the ^19^F NMR signal of a 4-fluorobenzyl group at the N-terminus of an oligothiourea appears downfield (−116.4 ppm) of the corresponding upfield ^19^F NMR signal (−117.9 ppm) at the C-terminus. It is important to note that, for this molecule, the two fluorine environments are always in slow exchange (at both 298 and 278 K, Figure 5c). For the corresponding proton environments to move into slow exchange, it is necessary to cool the sample to 248 K (Figure 5b).

To validate the *para*-fluorobenzyl groups as probes of conformational preference, *meta*-fluoro, *ortho*-fluoro, and *para*-trifluoromethyl benzyl groups were also investigated (Figure 6) through ^19^F VT NMR studies for compounds **22** (Figure 6b), **23** (Figure 6c), and **24** (Figure 6d). At 298 K, **24** (see Figure 6d) and **23** showed a single broad ^19^F NMR peak, while **22** showed two broad resonances. At lower temperatures (278 K), these signals decoalesced into two equally integrating signals for all of **22**, **23**, and **24**, separated, respectively, by 3.6 ppm, 1.9 ppm, and 2.0 ppm. The equal integration of all these pairs of signals is consistent with, and most parsimoniously explained by, a single, coherent intramolecular chain of hydrogen bonds which distinguishes the chemical environments of the ^19^F probe at the N-terminus (adjacent to the exposed NH group, in blue) and the C-terminus (adjacent to the exposed C=O group, in red). The environments of the two ^19^F probes move from intermediate to slow exchange as the rate of reversal of the directionality of this chain (and hence rate of dynamic exchange of N and C-termini) decreases on cooling. Although the *o*-F probe was more sensitive to its environment (presumably due to its proximity to the hydrogen-bond donor or acceptor), the risk of an interfering intramolecular hydrogen bond to F^28^ led us to prefer the *p*-F probe. ^19^F VT NMR analysis of **24** (Figure 6d) compared to **3** (Figure 3d) showed that the *p*-F probe in trimer **3** (1.7 ppm separation) was more sensitive than the *p*-CF_3_ in trimer **24** (0.3 ppm) (Figure 6d). In fact, it has been shown in proteins that a CF_3_ group is less sensitive to local electric fields and van der Waals interactions than a monofluorinated tag, resulting in a smaller chemical shift dispersion.^29,30^ We therefore used *p*-fluorobenzyl-capped *N*-butyl thiourea oligomers for the remainder of this work.

**Figure 6:**
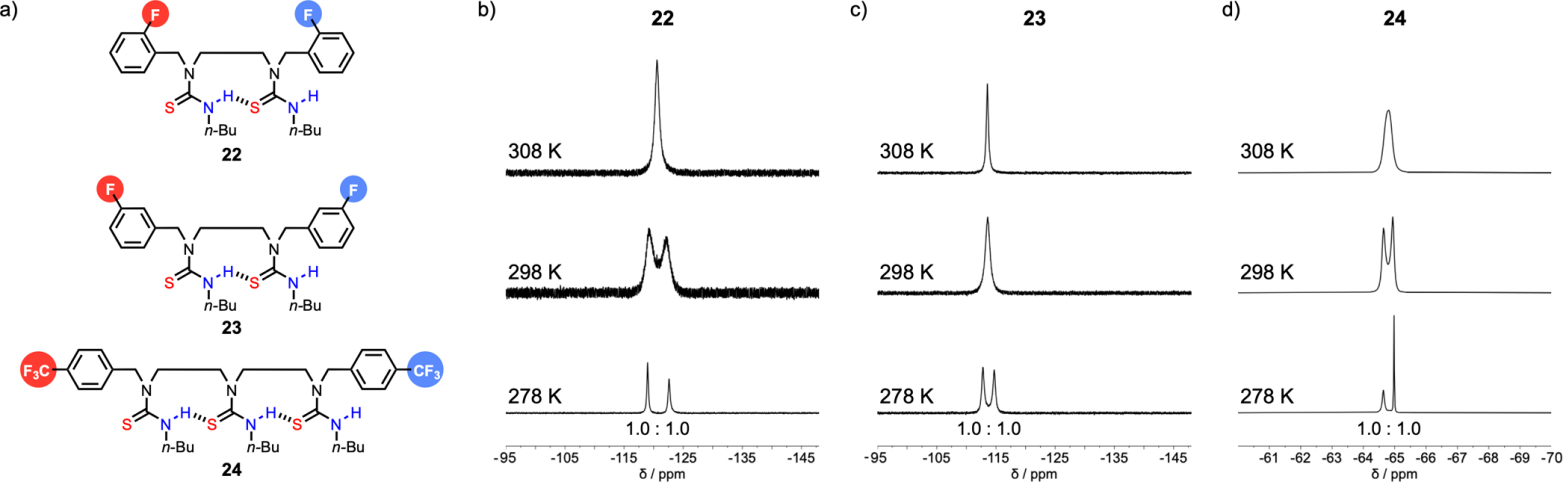
NMR study of oligothiourea foldamers **22**, **23**, and **24** in solution. (a) Molecular structures of **22**, **23**, and **24**. 565 MHz ^19^F VT NMR spectra of (b) **22**, (c) **23**, and (d) **24** (8 mM) at 308, 298 and 278 K in CDCl_3_.

#### ^1^H and ^19^F NMR Structural Study of **4**

Unlike the simple NMR spectra of conformationally uniform **3**, the ^19^F NMR spectra of tetramer **4** in CDCl_3_ at both 298 and 278 K (Figure 4d) reveal multiple conformers, which are in slow exchange at 278 K. These conformers must arise from rotamers that contain breaks in an otherwise coherent chain of hydrogen bonds. The occurrence of “flaws” in hydrogen-bonded foldamers has been explored in Aib-derived polyamide helices^31^ and their existence has been noted in oligourea foldamers,^4^ but quantitative analyses of the extent to which a foldamer may deviate from the ideal “specific compact conformation”^32^ are rare. Oligomer **4** therefore presents an opportunity to use structural information derived from ^19^F and 2D NMR experiments to explore the structure and dynamics of the variously hydrogen-bonded conformers that it populates.

Figure 7a shows the mechanism we propose for hydrogen-bond directionality reversal as a result of C–N bond rotations from **4a** to **4a′** via intermediates **4b**, **4c**, and **4b′**, which from here onward will also be called “broken” conformers. The degenerate conformers **4a** and **4a′** have a globally coherent hydrogen-bonded chain but with opposite directionality, thus resulting in two different environments for the fluorine at either end, shown in red (at the C-terminus) and in blue (at the N-terminus). Degenerate intermediates **4b** and **4b′** derived from **4a** and **4a′** by the rotation of one thiourea at either end of the foldamer. We expect the fluorine nuclei of **4b** and **4b′** to be characterized by different chemical shifts as one (in green) is at the N-terminus of a chain of three hydrogen-bonded thioureas, while the other one (in pink) is near only one non-hydrogen-bonded thiourea group. From these intermediates **4b** and **4b′**, another rotation of one of the central thioureas leads to the symmetric **4c** where both fluorines (in orange) are in the same environment at the N-terminus of a chain of two hydrogen-bonded thioureas. ^1^H–^1^H ROESY NMR at 248 K (Figure 7b) revealed that all the upfield (and thus non-hydrogen-bonded) NH peaks of **4** (see orange shading in Figure 7b) showed ROEs to the butyl urea CH_2_ peaks around 3.60 ppm and to the benzylic CH_2_ peaks at 4.63 ppm (i.e., H5). No ROEs were observed between these upfield NH peaks and the ethylene-bridge CH_2_ peaks of the foldamer backbone (broad peak around 4.00 ppm). Conversely, the downfield NH peaks (i.e., hydrogen-bonded, see blue shading in Figure 7b) have a ROE cross-peak with the butyl urea CH_2_ peaks and ethylene-bridge CH_2_ signals. These observations are consistent with the existence of conformers **4a**, **4a′**, **4b**, **4b′**, and **4c**, where the non-hydrogen-bonded NH points toward the *p*-fluorobenzyl capping groups, but not **4d**, **4d′**, or **4e** (Figure 7c) with outward-orientated C=S groups. Molecular mechanics calculations (see Section S5) corroborated these findings. Among the computed 50 lowest energy conformers for **4**, structures corresponding to both the “globally coherent” and “broken” conformers **4a**, **4b**, and **4c** were identified. However, even among the one hundred lowest energy structures of **4**, “broken” conformers **4d** and **4e** could not be found.

**Figure 7:**
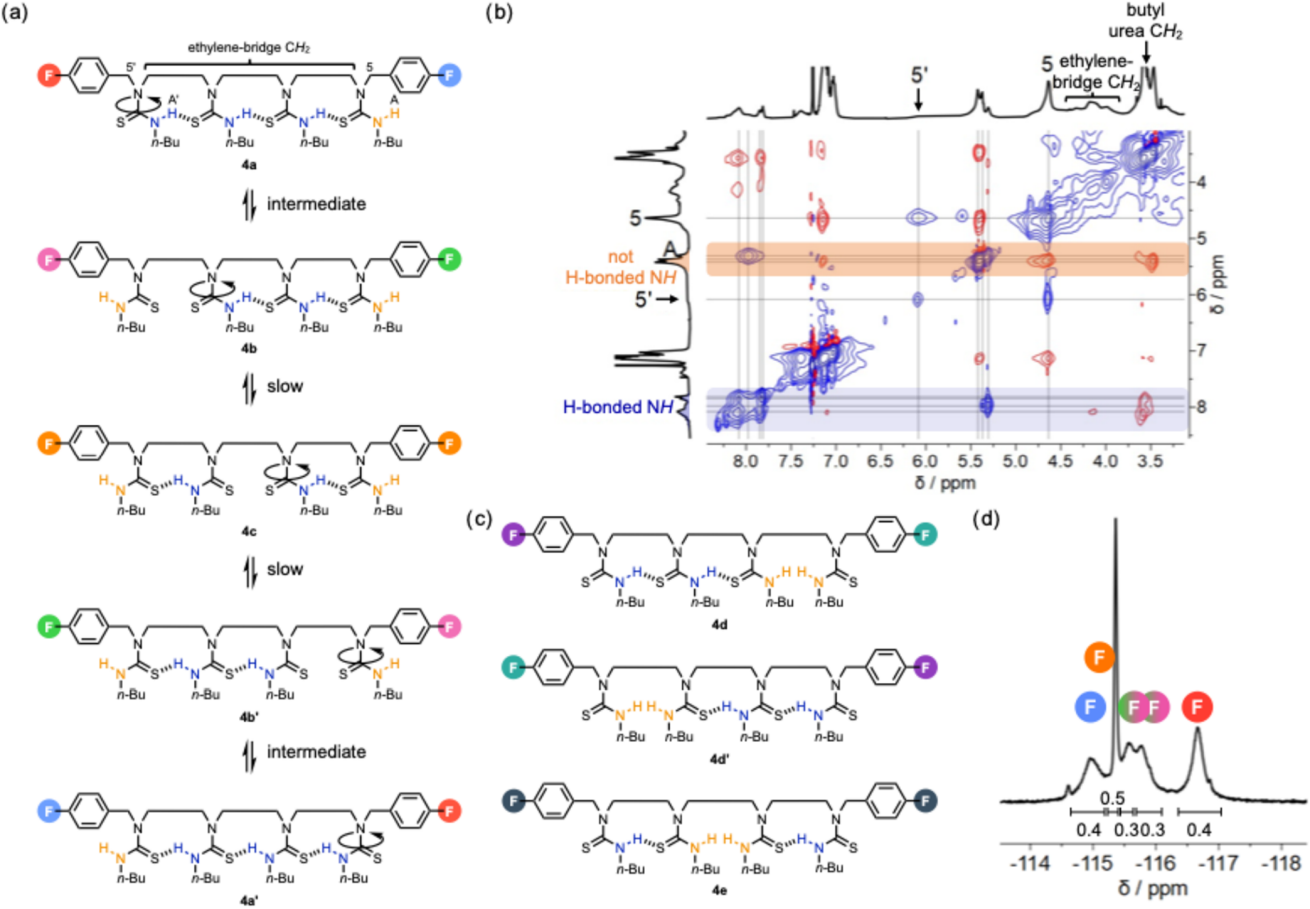
Structural study of oligothiourea foldamer **4**. (a) Proposed mechanism for reversal of global hydrogen bond directionality in 4 through “broken” conformers **4b**, **4b′**, and **4c**. (b) 500 MHz ^1^H–^1^H ROESY of **4** (8 mM) in CDCl_3_ at 248 K. An orange highlight shows that not hydrogen-bonded NHA has an exchange cross-peak with NHA′ (which is hydrogen bonded), an ROE cross-peak with H5, an ROE cross-peak with CH_2_ (in α to NH) of the butyl thiourea chain, and no ROE cross-peak with H5′. A blue highlight shows that hydrogen-bonded NH have exchange cross-peaks with other hydrogen-bonded NH, an exchange cross-peak with not hydrogen-bonded NHA, an ROE cross-peak with ethylene-bridge CH2, ROE cross-peaks with CH_2_ (in α to NH) of the butyl thiourea chain, and no ROE cross-peaks with either H5 or H5′. (c) Conformers **4d**, **4d′**, and **4e** not detected. (d) 471 MHz ^19^F NMR of **4** at 298 K with assignments.

Thus, it seemed likely that **4** populated the globally coherent conformer **4a** along with one or both of the “broken” conformers **4b**, **4b′**, and **4c** in Figure 7d. Considering the experimental evidence from the ROESY spectrum and by analogy with the ^19^F NMR peaks assignment of **3**, the five peaks visible in the ^19^F NMR spectrum of **4** at 298 K were assigned as follows. The equally integrating pair of ^19^F NMR peaks showing the greatest chemical shift separation was assigned to **4a** and **4a′**, with the N- and C-terminal fluorine nuclei (blue and red, respectively, assigned analogously to **3**). Of the three remaining peaks in the ^19^F NMR spectrum, two of these are broad but integrate equally, and so were assigned to **4b** and **4b′**, in which the fluorine nuclei (in green and pink) – while both adjacent to an NH – are nonetheless in slightly different environments. The chain of three hydrogen-bonded thioureas in **4b** and **4b′** likely benefits from cooperativity^33^ strengthening the intramolecular hydrogen bonds, which is lost when the thioureas are intramolecularly hydrogen bonded in two pairs, as they are in **4c**. Thus, we suggest that the single sharp peak is assigned to the fluorine nuclei (orange) in the symmetrical conformer **4c** in which symmetry places both fluorine probes in the same environment, and that **4c** is in slow exchange with **4b** and **4b′**. The fact that the ^19^F NMR peaks for these “broken” conformers have chemical shifts closer to the signal of the blue F at the N-terminus of the globally coherent conformer is consistent with their assignment to conformers in which the fluorine nuclei lie adjacent to terminal thiourea N–H (rather than C=S) bonds. This study corroborates a previous hypothesis that the global hydrogen-bond directionality inversion process in analogous oligoureas has been proposed to occur in a nonconcerted manner, with each urea unit rotating in turn.^4^ A similar phenomenon may give rise to the mixture of sharp and broad ^19^F NMR peaks observed for pentamer **5**. It is notable that no evidence for the population of conformers other than the fully hydrogen-bonded conformer of trimer **3** was seen at the temperatures studied by ^1^H and ^19^F NMR.

### Dynamic Conformation of Oligothiourea Foldamers in Membrane Mimics

Incorporation of foldamers into the membranes of liposomes could enable the development of new signal communication systems relying on a conformational change, and Aib-derived helical foldamers have already shown promise in this area.^8,10^ The exploration of foldamer conformations in the membrane phase is in its infancy, with only two examples of conformational analysis being reported.^11,12^ However, foldamers that have well-defined structures in both solution and in a membrane offer opportunities rarely presented by biogenic molecules to compare behavior of the same molecule in both isotropic and nonisotropic environments.

Having successfully validated ^19^F NMR as a means of exploring the conformation of oligothiourea foldamers in solution, we set about extending the use of the technique to the membrane phase. We initially explored small unilamellar vesicles (SUVs) as a membrane environment that would both solubilize the foldamers and allow the acquisition of well-resolved spectra to study their dynamic behavior. However, a significant increase in the line width of the signal was observed for the tested pentamer foldamers, presumably due to increased rotational correlation time of the lipid vesicles^34,35^ (see Section S4.9 in the Supporting Information).

We therefore tried two alternative rapidly reorienting membrane-mimetic environments suitable for high-resolution NMR studies: micelles and bicelles. Both micelles^36^ and bicelles^37^ are dynamic assemblies on the NMR time scale, undergoing rapid molecular exchange and structural fluctuations, which may contribute to the averaging of the environments experienced by the embedded foldamers.

Micelles are nanometer-scale spherical particles suspended in an aqueous solution consisting of aggregated detergent molecules with a hydrophobic core.^37^ In particular, micelles composed of sodium dodecyl sulfate (SDS) have been extensively employed for structural characterization of membrane proteins through solution NMR techniques.^38–40^ SDS micelles were prepared by suspending the detergent in MOPS (20 mM, pH 7.4), NaCl (100 mM), and KF (0.05 mM, $F_{\left(\mathrm{aq}\right)}^{-}$ was used as a reference for the ^19^F NMR spectra). In order to study the dynamic conformation of compounds **1**–**5** in micelles, an aliquot of the desired compound dissolved in 9:1 MeOD/*d*_6_-DMSO was added to the colloidal suspension. Dynamic light scattering (DLS) of the micellar suspensions showed that their size was unchanged after incorporation of the foldamer (see Section S3.4 of the Supporting Information). The successful incorporation of reference compound **1** and foldamers **2**–**5** into micelles was confirmed by the observation of signals characteristic of each compound when the micellar suspensions were analyzed by ^1^H and ^19^F VT NMR spectroscopy (Figure 9).

Figure 8 shows the aromatic region of the ^1^H NMR spectra and the ^19^F NMR spectra at 318, 298 and 288 K in micelles. (Cooling to 278 K led to precipitation.) Sharp signals were noted in both ^1^H and ^19^F spectra of all the investigated compounds **1**–**5** unless slow exchange processes were occurring. The region of the ^1^H NMR spectra at chemical shifts lower than 5 ppm is dominated by the residual water solvent peak and by the signals of the buffer (MOPS) and of the detergent (SDS), but suppression of the water signal by presaturation^41^ reveals the signals of aromatic protons H2,2′ and H3,3′ of the various compounds.

**Figure 8:**
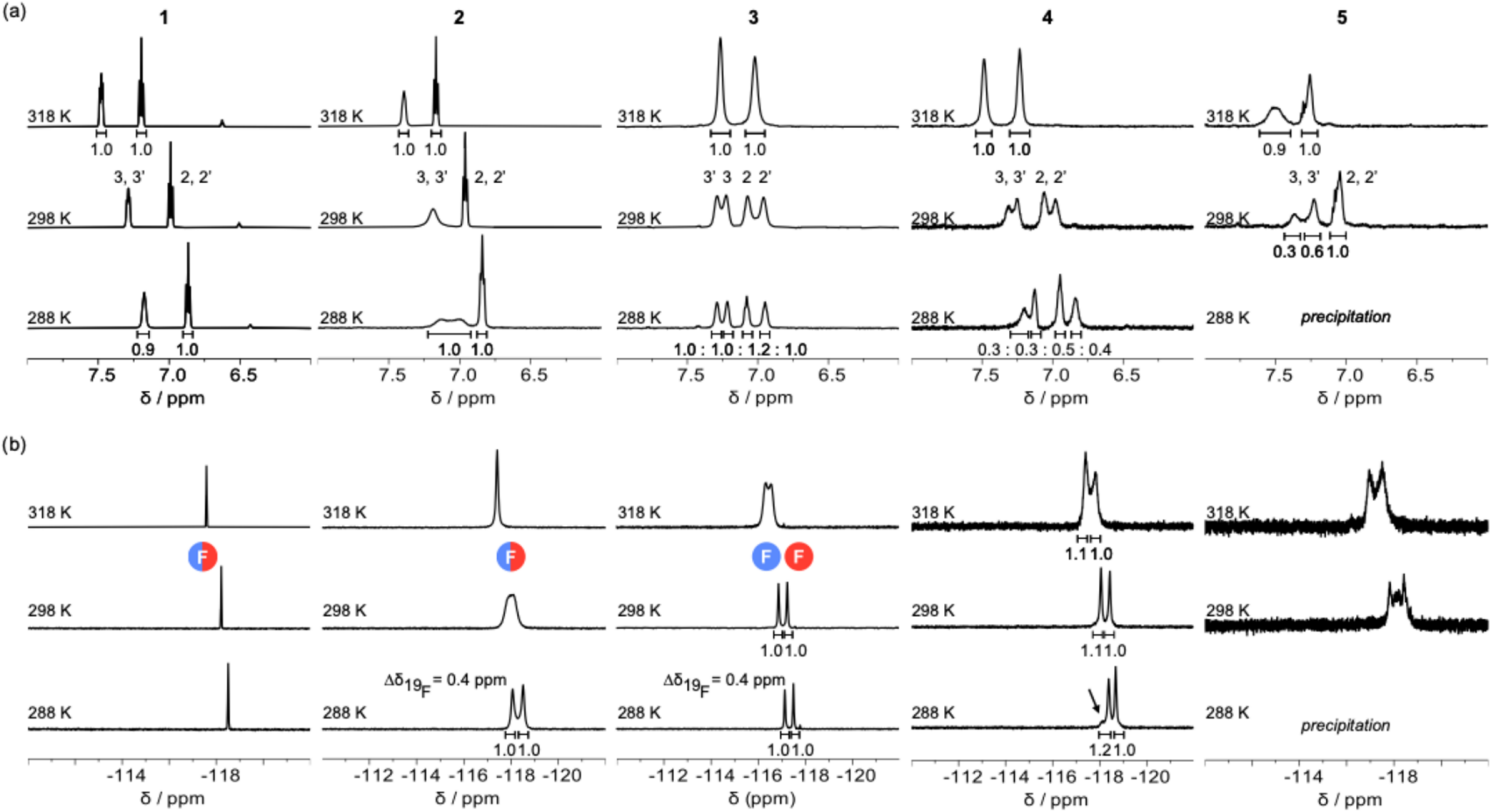
(a) 600 MHz ^1^H (with presaturation^42^ for the suppression of the water signal) and (b) 565 MHz ^19^F VT NMR spectra at 318, 298 and 288 K of compounds **1**–**5** (1 mM) embedded in micelles (SDS, 200 mM) suspended in MOPS (20 mM, pH 7.4), NaCl (100 mM), KF (0.05 mM) in D_2_O.

Confounding signals from solvent and SDS may be completely eliminated by using ^19^F NMR spectroscopy, since only the incorporated foldamers contain fluorine atoms. Reference compound **1**, in micelles between 318 and 288 K, shows two peaks with characteristic multiplicities for H3,3′ (downfield) and H2,2′ (upfield) in the ^1^H spectrum (Figure 8a) and one sharp peak in the ^19^F NMR (Figure 8b), indicating fast C–N bond rotation on the NMR time scale. In addition, the micellar environment shields the ^19^F nuclei of **1** by 1.9 ppm, leading to an upfield shift of the peak from −116.3 ppm in CDCl_3_ to −118.2 ppm in micelles.

Compound **2** in micelles exhibits in the ^1^H NMR spectrum (Figure 8a) a sharp peak for H2,2′ and a peak for H3,3′ that broadens into intermediate exchange at 288 K, and in the ^19^F NMR spectrum (Figure 8b) a sharp peak at 318 K that decoalesces in two separate peaks at 288 K. We therefore conclude that in micelles dimer **2** populates just two identical hydrogen-bonded conformations with opposite hydrogen bond directionality, having a single intramolecular hydrogen bond that persists when **2** is embedded in the micelle. The slow-exchange chemical shift difference between the two decoalesced fluorine peaks is ∼2.0 ppm in CDCl_3_ (at 278 K) and ∼0.4 ppm in micelles (at 288 K), suggesting more equalized environments for at the N- and C-termini once the foldamer is incorporated in micelles.

Compound **3** showed at 298 K four equally integrating peaks in the ^1^H spectrum for H2, H2′, H3, H3′ (Figure 9a, assigned by analogy with spectra in CDCl_3_ and CD_2_Cl_2_) and two separate equally integrating peaks in the ^19^F NMR (Figure 8b). As the temperature was raised to 318 K, the proton signals coalesced into two separate peaks while the fluorine peaks appeared broadened, demonstrating that in micelles trimer **3** exists as two degenerate equilibrating conformers with opposite hydrogen-bond directionality, which remain in slow exchange at 298 K. In this case, the difference between the chemical shifts of the N- and C-terminal F nuclei decreases from 1.8 ppm in CDCl_3_ (at 278 K, Figure 4d) to 0.4 ppm in micelles (at 288 K).

**Figure 9:**
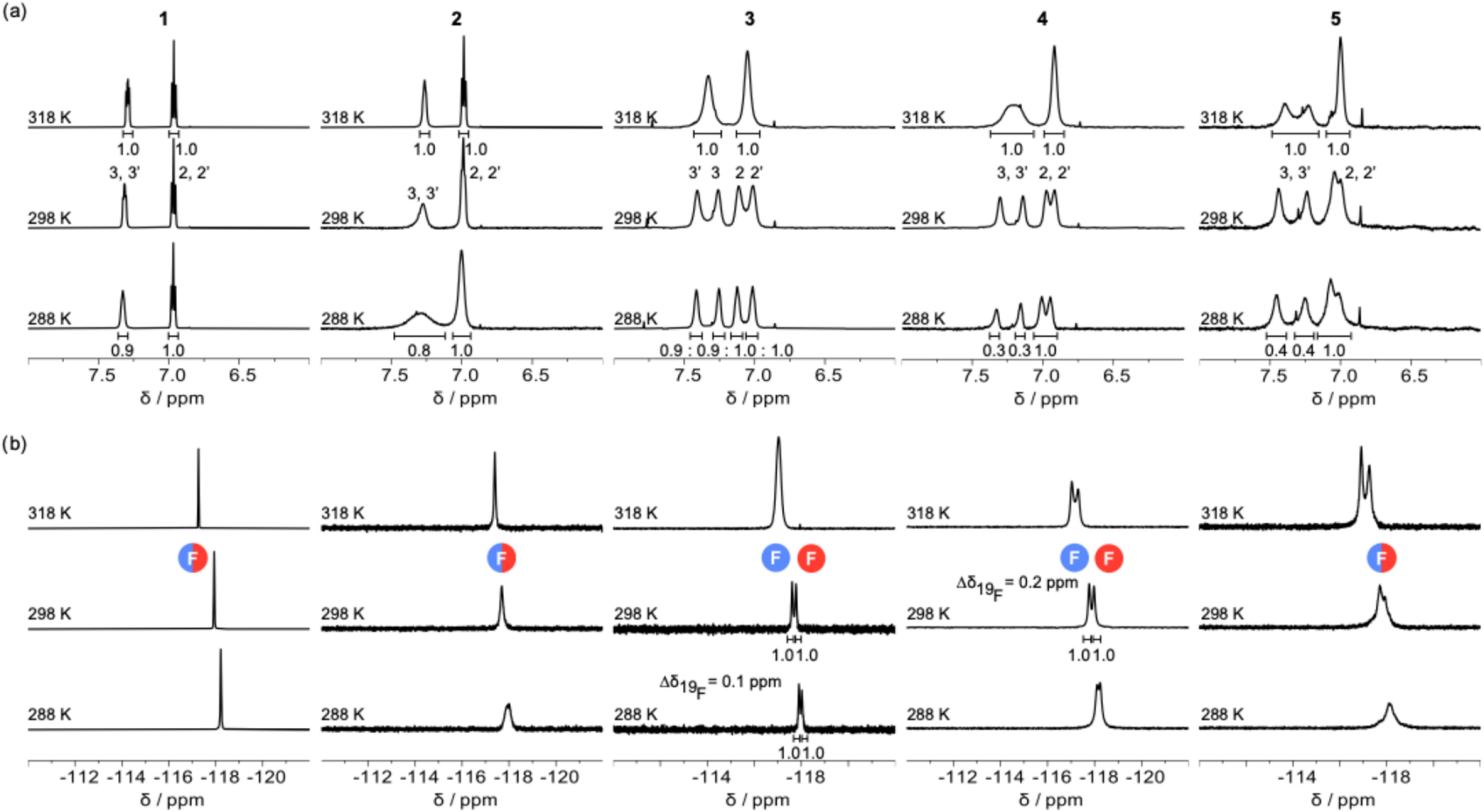
(a) 600 MHz ^1^H (with presaturation^42^ for the suppression of the water signal) and (b) 565 MHz ^19^F NMR spectra at 318, 298 and 288 K of compounds **1**–**5** (1 mM) embedded in bicelles (DLPC:DHPC, 300 mM, *q* = 0.5) suspended in MOPS (20 mM, pH 7.4), NaCl (100 mM), KF (0.05 mM) in D_2_O.

With the third intramolecular hydrogen-bond of tetramer **4**, the ^1^H NMR spectrum (Figure 8a) contains four broad peaks at 288 K, which coalesce into two equally integrating signals at 318 K. The ^19^F NMR (Figure 8b) shows two peaks with different integrals at all temperatures and at 288 K also a small peak at higher chemical shifts (see arrow in Figure 8b). The downfield signal of the fluorine at the N-terminus integrates more than the upfield fluorine at the C-terminus similarly to **4** in CDCl_3_. This suggests that in micelles at 298 K, a small population of “broken” conformers, with the NH groups pointing toward the capping group, contribute to this integral, although less than in CDCl_3_ (integrals 1.4:1.0 in CDCl_3_ in Figure 4d versus 1.1:1.0 in micelles in Figure 9b at 298 K). It is clear that for **4**, both in solution and in micelles, ^19^F NMR reveals the presence of minor conformers more readily than does ^1^H NMR. This increased sensitivity is particularly visible in the case of foldamer **5**. In the ^1^H spectrum (Figure 8a) only the peaks corresponding to H3 and H3′ appear decoalesced at 298 K, and in the ^19^F spectrum multiple peaks reveal the population of both the coherent and “broken” conformers of **5** in the micellar environment.

Micelles have a monolayer structure with a high curvature, with loosely packed head-groups and tight chains entangled in the hydrophobic core. These features mean that micelles differ significantly from the biological membrane morphology.^42^ By contrast, bicelles consist of a disc-shaped phospholipid bilayer, surrounded by a rim of short-chain phospholipid molecules, and have been used for NMR studies of membrane-associated peptides and proteins.^43–46^ Specifically, a mixture of 1,2-dilauroyl-*sn*-glycero-3-phosphocholine (DLPC) and 1,2-dihexanoyl-*sn*-glycero-3-phosphocholine (DHPC) with a q ratio of 0.5 ^47^ mimics the phospholipid bilayer of a liposome, but on a much smaller scale than SUVs (6 nm diameter for bicelles versus about 100 nm for SUVs), which minimizes the broadening of NMR peaks.

Similar to SDS micelles, DLPC:DHPC bicelles were prepared by suspending the lipids in the same aqueous buffer, followed by the addition of an aliquot of foldamer dissolved in 9:1 MeOD/*d*_6_-DMSO to the colloidal suspension.

Reference compound **1** and foldamers **2**–**5** were evidently incorporated into bicelles, as distinct signals characteristic of each compound were visible when the bicelle suspensions were analyzed by ^1^H and ^19^F NMR spectroscopy (Figure 9). Analogously to Figure 8 for micelles, Figure 9 shows the aromatic region of the ^1^H NMR spectra (with the signals for H3,3′ and H2,2′) and the ^19^F NMR spectra at 318, 298 and 288 K of a series of foldamers in bicelles. As in CDCl_3_ and micelles, the signals for reference compound **1** are in fast exchange at all temperatures investigated in both the ^1^H and ^19^F NMR spectra. The decrease of the temperature from 318 to 288 K led to shielding of the fluorine peak by 1 ppm, but, unlike in micelles, the signals of H3,3′ and H2,2′ were relatively insensitive to the temperature change. Dimer **2** showed two broad peaks (especially for H3,3′) in the ^1^H NMR spectrum at 288 K (intermediate exchange) that sharpened at 318 K (fast exchange), and likewise a broad fluorine peak that moved from intermediate at 288 K to fast exchange at 318 K. Bicelle suspensions incorporating the foldamers did not precipitate at 278 K, so it was possible to cool all the samples to lower temperatures. In the case of **2**, this revealed the decoalescence of both the H3,3′ signal in the ^1^H NMR and the fluorine signal in the ^19^F NMR into two equally integrating peaks. Being able to identify the two signals in slow exchange confirms that, as in micelles, two interconverting degenerate conformers of **2** are present in bicelles, with an intact intramolecular hydrogen bond. The addition of a second intramolecular hydrogen bond in trimer **3** further slows down the directionality interconversion, as even at 298 K, it is possible to observe four peaks in the ^1^H NMR spectrum for the aromatic protons and two peaks in the ^19^F NMR. The comparison of the ^19^F NMR spectra of **3** in CDCl_3_ at 278 K (Figure 4d), in micelles at 288 K (Figure 8b) and in bicelles at 288 K (Figure 9b) reveals that the chemical shift difference between the two fluorine peaks, and thus between the F environments at the C- and N-termini, is even smaller in bicelles (0.1 ppm).

The ^1^H NMR of **4** suggested that at 288 K two identical “coherent” conformers are populated, since both H3,3′ and H2,2′ are in slow exchange and transition to intermediate exchange at 318 K. However, VT NMR of tetramer **4** in both CDCl_3_ and micelles showed additionally the presence of “broken” conformers. In the bicelle, ^19^F NMR reveals a spectrum in which the two ^19^F NMR peaks integrate identically, i.e., 1.0:1.0, at 298 K, suggesting that in bicelles the “broken” conformers of **4** cannot be detected at this temperature, possibly due to the small difference in chemical shifts between the two F peaks (only 0.2 ppm at 298 K). Pentamer **5** exhibits broad peaks in both the ^1^H and ^19^F NMR spectra at 288 K that sharpen as the temperature increases to 318 K. Unlike for **4**, “broken” conformers of **5** can be detected in bicelles, as the F peak at 298 K appears to be a broad combination of multiple overlapping signals. For trimer **24**, at 288 K, all the aromatic protons and the fluorine are in slow exchange but move to fast exchange at 318 K, confirming the presence of two conformers. The separation of the two F peaks at 288 K is 0.1 ppm, which is comparable to that of **3** in bicelles.

In the case of dimer **2**, VT NMR studies showed that it is possible to detect the two conformers with opposite hydrogen-bond directionality in both the fast and in the slow exchange regime not only in solution but also in membrane mimics. This compound was chosen as a case study to determine the barriers to the interconversion of the two conformers in CD_2_Cl_2_, micelles and bicelles (Figure 10). Lineshape analysis of the ^19^F VT NMR spectra of **2** in different media (see Section S4.6) allowed us to determine the following free Gibbs energy ${\Delta G}_{298\;K}^{‡}$ values associated with the hydrogen-bond polarity reversal: 51.5 kJ mol^–1^ in CD_2_Cl_2_, 56.7 kJ mol^–1^ in micelles and 55.9 kJ mol^–1^ in bicelles. Colloidal particles exhibit temperature-dependent dynamics, with acyl chain packing and disorder influencing molecular mobility.^48^ However, the small differences in ${\Delta G}_{298\;K}^{‡}$ observed between solution, micelles and bicelles suggest that in the membrane environments the effect is modest. This is consistent with the dynamic nature of SDS micelles^49^ and the fluid DLPC:DHPC bicelles in which strong packing effects are less pronounced than in extended bilayers.^37^ We believe this to be the first use of dynamic NMR to evaluate the energetics of conformational interconversion of a synthetic molecule in a membrane environment. For amide-like C–N bonds, the barrier of rotation increases with increasing solvent polarity.^50^ However, we have previously shown that for a foldamer containing two intramolecularly hydrogen-bonded ureas, the addition of a polar solvent to CD_2_Cl_2_ resulted in a decrease of the rotation barrier, presumably due to hydrogen bonding to these more polar solvents reducing the enthalpic cost of breaking an intramolecular hydrogen bond to switch the directionality.^4^ Therefore, we hypothesize that the membrane-mimetic environments in the micelles and the bicelles where the compounds are embedded are less polar than CD_2_Cl_2_, which is also supported by the shielding of the fluorine signals. However, these environments are highly inhomogeneous systems in which polarity varies with depth,^51^ and molecules may adopt interfacial or partially inserted states rather than residing in the bilayer center.^52^

**Figure 10:**
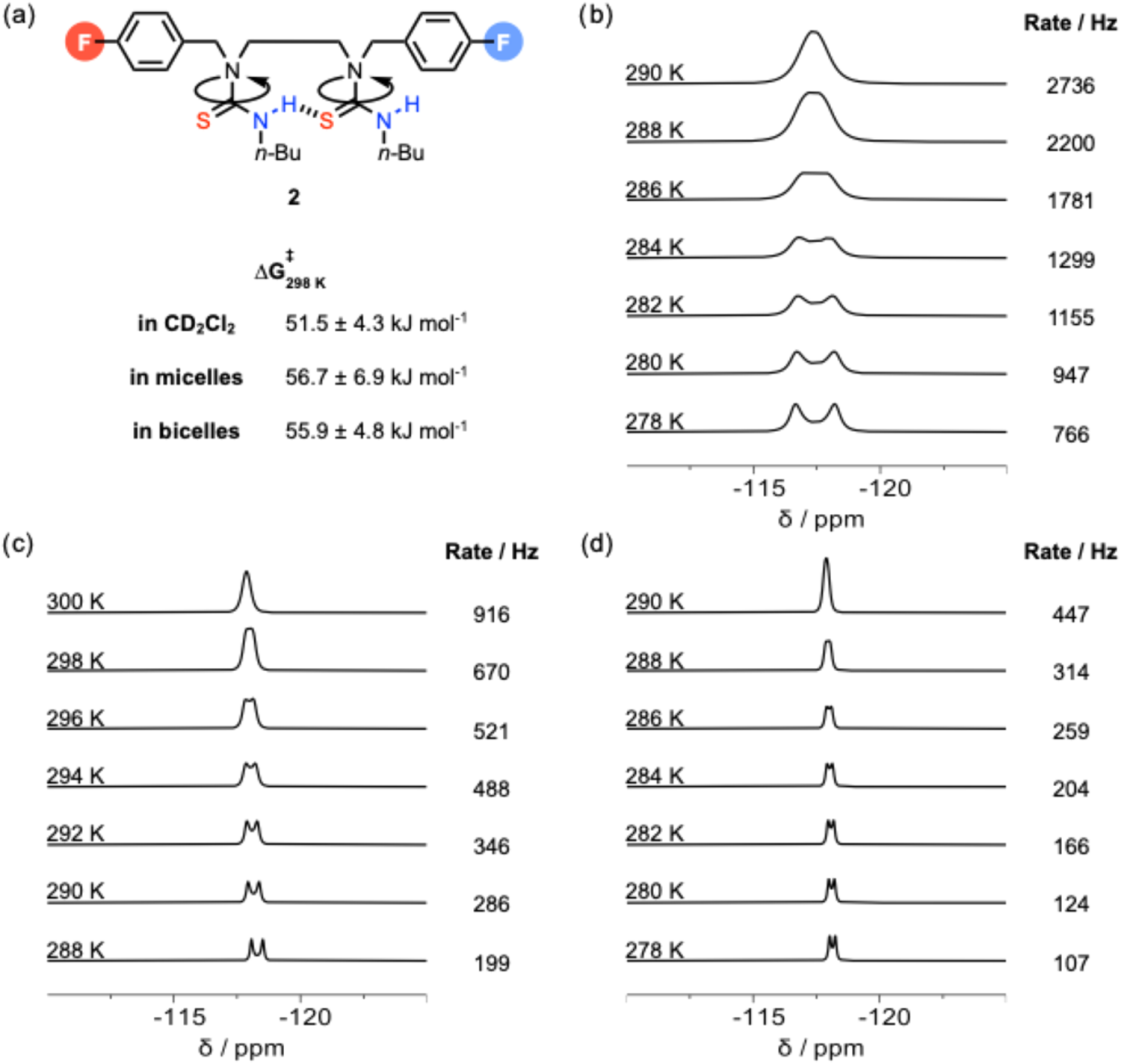
Conformational dynamics of **2**. (a) Gibbs free energy ${\Delta G}_{298\;K}^{‡}$ for C–N bond rotation determined through line shape fitting of the ^19^F VT NMR spectra of **2** in (b) CD_2_Cl_2_, (c) micelles (SDS, 200 mM) and (d) bicelles (DLPC:DHPC, 300 mM, *q* = 0.5). Both micelles and bicelles were suspended in MOPS (20 mM, pH 7.4), NaCl (100 mM), KF (0.05 mM) in D_2_O.

## Conclusions

In conclusion, we have reported a family of ethylene-bridge oligothiourea foldamers whose structure contains a single intramolecular sequence of N–H···S=C hydrogen bonds. The high rotational barrier around the thiourea C–N bond (relative to the corresponding urea) and the strong intramolecular H-bonds allow the two degenerate, fully hydrogen-bonded conformers for foldamers **2**–**5** to be detected above 278 K by NMR spectroscopy. In the longer oligomers **4** and **5**, additional “broken” conformers, arising from stepwise breaks in the chain of hydrogen bonds during polarity reversal, were observed and characterized for the first time. ^19^F NMR probes proved especially powerful in revealing these conformational features in solution and were likewise applicable to the study of the foldamers embedded into membrane mimics.

Comparison across solution, micelles, and bicelles reveals clear differences in conformational behavior. In a chloroform solution, the hydrogen-bonded chain is well-defined, but conformational heterogeneity increases with oligomer length. Trimer **3** populates almost exclusively the two fully coherent degenerate conformers, while tetramer **4** and pentamer **5** additionally populate “broken” intermediates. The ^19^F chemical shift separation between the N- and C-terminal probes is largest in solution, reflecting strongly differentiated terminal environments and pronounced hydrogen-bond polarity.

In SDS micelles, the foldamers are incorporated within an apolar membrane-mimetic region and retain an intact hydrogen-bond network. The same two degenerate conformers are observed for shorter oligomers, and minor broken conformers remain detectable for **4** and **5**. However, the chemical shift separation between terminal fluorine probes is significantly reduced compared to solution, indicating similar N- and C-terminal environments within the curved micellar assembly. Although conformational exchange remains accessible on the NMR time scale, the “broken” intermediates are less populated than in organic solvent.

In DLPC:DHPC bicelles, which more closely resemble a phospholipid bilayer, the terminal environments become even more similar. The ^19^F chemical shift differences between N- and C-termini are further compressed, and for tetramer **4** the “broken” conformers are no longer readily distinguishable at 298 K, although they remain detectable for pentamer **5**. Thus, while the hydrogen-bonded chain remains resilient and directionally reversible, conformational heterogeneity and the spectroscopic visibility of intermediates decrease progressively from solution to micelles to bicelles.

Dynamic NMR analysis of dimer **2** further demonstrated that hydrogen-bond polarity reversal occurs with comparable but slightly higher rotational barriers in micelles and bicelles than in an organic solvent, consistent with incorporation into a less polar membrane-mimetic environment. This study therefore establishes that these oligothiourea foldamers retain their directional hydrogen-bond networks and dynamic switching behavior not only in solution but also within membrane-mimetic systems.

This work provides the first instance in which bicelles have been used to incorporate a synthetic, non-biological molecule to enable quantitative analysis of its dynamic conformational behavior by NMR spectroscopy. The ability to monitor hydrogen-bond resilience, directionality, and intermediate populations in both isotropic and membrane-mimetic environments will be crucial for the development of synthetic communication systems that exploit hydrogen-bond polarity to transmit signals across lipid bilayers.

## Supplementary Material



## Notes

Materials and Methods, detailed synthetic procedures, NMR experiments, computational study (PDF)

## Abbreviations

DHPC: 1,2-dihexanoyl-*sn*-glycero-3-phosphocholine
DLPC: 1,2-dilauroyl-*sn*-glycero-3-phosphocholine
DLS: dynamic light scattering
DOPC: 1,2-dioleoyl-*sn*-glycero-3-phosphocholine
MOPS: 3-(*N*-morpholino)­propanesulfonic acid
NMR: nuclear magnetic resonance
ROESY: rotating frame overhauser enhancement spectroscopy
SDS: sodium dodecyl sulfate
SUVs: small unilamellar vesicles
VT: variable temperature
