# Supplementary material for: Hydrogen Bonding within Dynamic 19F‑Tagged Oligothiourea Foldamers in Solution and in Membranes
Source: J Am Chem Soc. 2026 Jun 17;148(25):26171–83. doi: 10.1021/jacs.6c05314 (PMC13339130; doi:10.1021/jacs.6c05314)
Supplement: Supplementary file 1 [file ja6c05314_si_001.pdf]

Supporting Information

Hydrogen Bonding within Dynamic ^{19}F-Tagged Oligothiourea Foldamers in Solution and in Membranes

Lucia Trevisan‡* Kathryn S. Foster‡ and Jonathan Clayden*

‡ These authors contributed equally.

Table of Contents **Page**

Table of Contents	Page
S1 Materials and Methods	S2
S2 Synthesis	S4
S3 Membrane Preparation	S33
S3.1 General Procedure for Vesicle Preparation	S33
S3.2 General Procedure for Micelle Preparation	S33
S3.3 General Procedure for Bicelle Preparation	S33
S3.4 Dynamic Light Scattering	S34
S4 NMR Studies	S36
S4.1 ^{19}F Referencing	S36
S4.2 VT NMR in $CDCl_3$	S37
S4.3 2D NMR in $CDCl_3$ at 248 K	S47
S4.4 Assignment of the ^{19}F NMR spectrum of **3**	S48
S4.5 VT NMR in micelles	S51
S4.6 VT NMR in bicelles	S54
S4.7 Lineshape analysis of VT NMR of **2** and **3** in CD_2Cl_2, micelles and bicelles	S57
S4.8 Insolubility of Foldamers in Water	S61
S4.9 Foldamers **24-27** in $CDCl_3$, vesicles, micelles and bicelles	S62
S5 Computational Study	S72
S6 References	S73

S.1 Materials and Methods

Reagents and solvents: Reagents and non-anhydrous solvents (acetonitrile [MeCN], chloroform [$CHCl_3$], dichloromethane [CH_2Cl_2], *N*,*N*-dimethylformamide [DMF], ethanol [EtOH], ethyl acetate [EtOAc], diethyl ether [Et_2O], iso-propanol [IPA], methanol [MeOH], petroleum ether 40-60 °C [petrol], and tetrahydrofuran [THF]) were purchased from commercial suppliers (Sigma-Aldrich, Fluorochem, Merck) and used without further purification. Anhydrous solvents were commercially supplied (Sigma-Aldrich) or dried using an Anhydrous Engineering Grubbs-type solvent system. Lipids and detergents (Avanti Research^{TM}, Sigma-Aldrich) were purchased from Merck and used without further purification.

Inert techniques: Where specified, procedures were performed under a nitrogen atmosphere and air and moisture-sensitive liquids were transferred to reaction flasks via syringe under a nitrogen atmosphere. Room temperature (rt) ranged between 15-25 °C.

Stirring: Agitation of reaction mixtures was achieved using Teflon-coated stirrer bars by magnetic induction.

Chromatography: Thin layer chromatography (TLC) experiments were conducted using pre-coated plastic plates and visualised using ultraviolet (UV) light (254 nm) or staining ($KMnO_4$ or ninhydrin). Flash chromatography was performed on an automated Biotage IsoleraTM Spektra Four with gradient elution on pre-packed silica gel Sfär Duo columns.

NMR experiments: All room temperature NMR experiments (25 °C) were performed using a Bruker Avance spectrometer (400 MHz), a Jeol ECZ spectrometer (400 MHz), a Bruker Avance III HD spectrometer with a 5 mm Cryo probe (500 MHz), a Bruker Avance III HD spectrometer with a 5 mm TCI Prodigy probe (500 MHz), or a Bruker Neo spectrometer with a 5 mm TXO Cryo Probe (600 MHz). ^1H and ^{13}C NMR chemical shifts (δ) are reported in parts per million (ppm) relative to residual solvent.[1] ^{19}F NMR chemical shifts were referenced through use of a sealed glass capillary containing C_6F_6 (δ_F = -164.90 ppm, 8.5 mM) in CD_2Cl_2 placed inside the NMR tube. Variable Temperature NMR (VT NMR) experiments were performed using the Bruker Avance III HD spectrometer with a 5 mm Cryo probe (500 MHz) or the Bruker Neo spectrometer with a 5 mm TXO Cryo Probe (600 MHz). NMR spectra were processed in MestreNova. The multiplicities of NMR signals are reported as follows: s = singlet, d = doublet, t = triplet, q = quartet, quint = quintet, sext = sextet, m = multiplet, br = broad, or some combination thereof. Spin-spin coupling constants (*J*) are reported in hertz (Hz) to the nearest 0.1 Hz. 2D NMR experiments (^1H-^1H COSY, ^1H-^{13}C HSQC, ^1H-^{13}C HMBC, and ^1H-^1H TOCSY) were used, where necessary, to assign ^1H and ^{13}C NMR spectra.

Mass spectrometry: High-resolution mass spectrometry experiments (HRMS) were recorded by technical staff at the University of Bristol on a Bruker microOTOF II spectrometer using electrospray ionisation (ESI), a Thermo Scientific Orbitrap Elite spectrometer using ESI, nanospray, or

atmospheric pressure chemical ionisation (APCI), or a Waters Synapt G2S spectrometer using nanospray. Only molecular ion ($[M+H]^+$ or $[M+Na]^+$) peaks are reported.

Melting points: Melting points (mp) were recorded on a Stuart SMP30 melting point machine following recrystallisation from the specified solvent(s).

Infrared spectroscopy: Fourier transform infrared (IR) spectra were recorded neat as a thin film using a PerkinElmer Spectrum Two FTIR Spectrometer. Signals are described as follows: s = sharp, m = medium, br = broad. All absorbances are reported in cm^{-1}.

pH-meter: pH of the buffer solution was measured with a Jenway 3510 pH meter equipped with a Hanna HI2130 electrode.

Dynamic light scattering spectroscopy: DLS was conducted using a Malvern Instruments Zetasizer Nano-S fitted with a 4 mW He-Ne laser (633 nm).

S.2 Synthesis

In order to validate the ^{19}F probe and the choice of thioureas over ureas, additional ethylene-bridged oligo(thio)ureas **22-27** (Figure S1) were synthesized (Schemes S1 and S2) analogously by using an alternative aldehyde in the reductive amination (**22**, **23**, **24**), phenyl isothiocyanate (**25**), isocyanates in place of isothiocyanates (**27**), or a combination thereof (**26**).

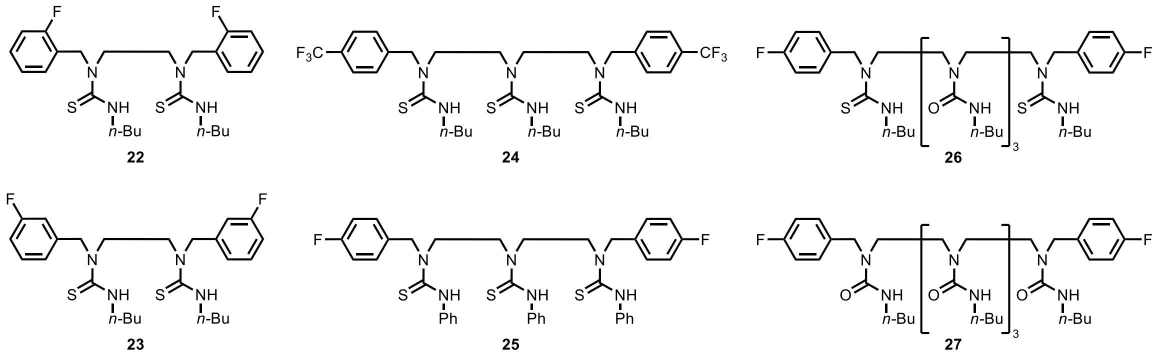

Figure S1. Other synthesized oligo(thio)thiourea foldamers **22-27**.

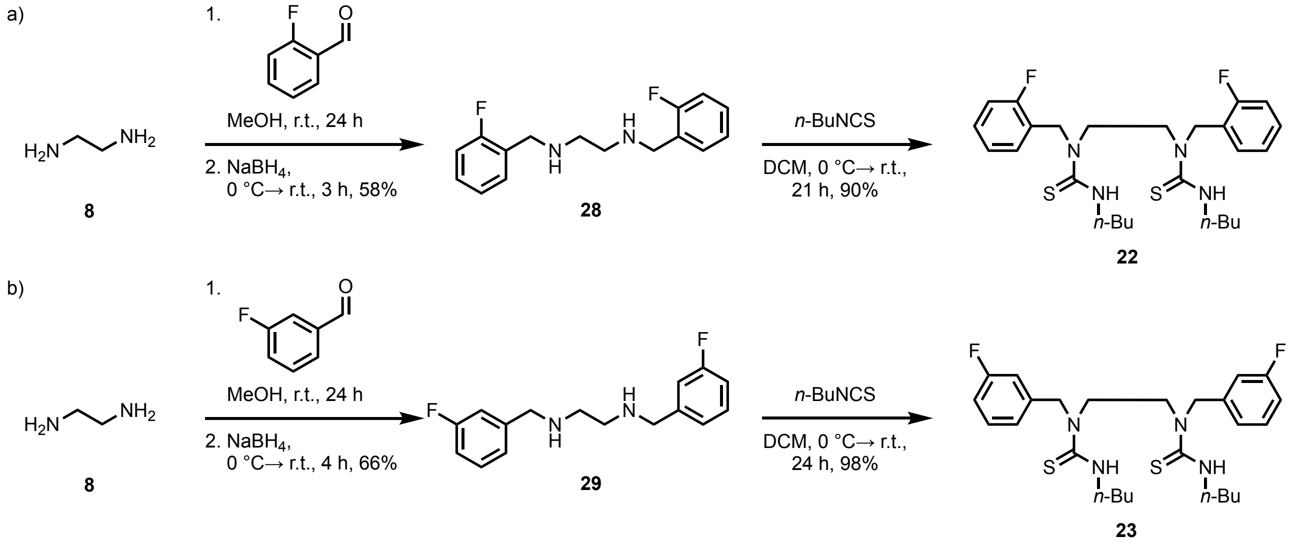

Scheme S1. Synthesis of foldamers with the fluorine probe (a) in *ortho* **22** and (b) in *meta* position **23** on the benzyl capping groups.

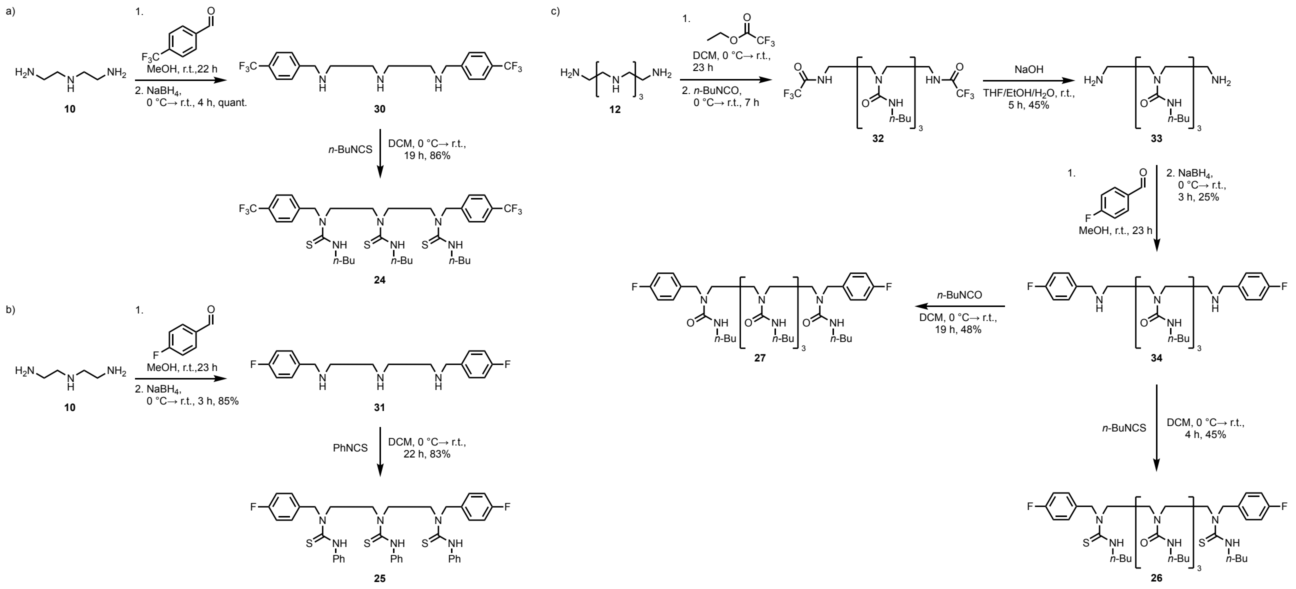

Scheme S2. Synthesis of foldamers (a) **24**, (b) **25**, (c) **26** and **27**.

Bis(4-fluorobenzyl)amine (7)

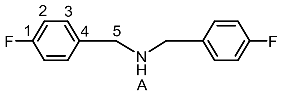

To a stirred solution of 4-fluorobenzylamine (0.57 mL, 5.0 mmol, 1.0 eq.) in MeOH (50 mL) was added 4-fluorobenzaldehyde (0.38 mL, 3.5 mmol, 0.7 eq.) under air. The reaction mixture was stirred at r.t. for 25 h. The reaction mixture was then cooled to 0 °C and $NaBH_4$ (227 mg, 6.0 mmol, 1.2 eq.) was added portionwise. The reaction mixture was warmed to r.t. and stirred for 3 h. The reaction mixture was quenched with aqueous NaOH (1 M) and diluted with 3:1 $CHCl_3$/IPA. The organic layer was separated, and the aqueous layer was extracted three times with 3:1 $CHCl_3$/IPA. The organic extracts were combined, dried over $MgSO_4$, filtered, and concentrated *in vacuo*. The crude residue was purified by flash column chromatography (SiO_2, loading in CH_2Cl_2; gradient elution: 100:0:0 CH_2Cl_2/MeOH/[35% aqueous NH_3] to 95.2:4.0:0.8 CH_2Cl_2/MeOH/[35% aqueous NH_3]) to yield the title compound as a colourless liquid (655 mg, 80%).

Data consistent with literature.[2]

^{1}H NMR (400 MHz, $CDCl_3$) δ_H: 7.30 (4H, dd, *J* = 8.5, 5.3 Hz, *H*2), 7.02 (4H, t, *J* = 8.5 Hz, *H*3), 3.76 (4H, s, *H*5), 1.57 (1H, br s, N_A*H*).

^{13}C NMR (101 MHz, $CDCl_3$) δ_C: 162.1 (d, *J* = 244.8 Hz, *C*1), 136.0 (d, *J* = 3.2 Hz, *C*4), 129.8 (d, *J* = 8.0 Hz, *C*2), 115.3 (d, *J* = 21.0 Hz, *C*3), 52.5 (s, *C*5).

^{19}F NMR (376 MHz, $CDCl_3$) δ_F: –115.85 (tt, *J* = 9.3, 5.2 Hz).

3-Butyl-1,1-bis(4-fluorobenzyl)thiourea (1)

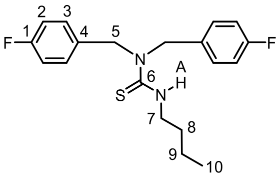

To a stirred solution of **7** (146 mg, 0.63 mmol, 1.0 eq.) in CH_2Cl_2 (20 mL) at 0 °C was added *n*-butyl isothiocyanate (81 μL, 0.69 mmol, 1.1 eq.) dropwise under air. The reaction mixture was warmed to r.t. and stirred for 24 h. The reaction mixture was quenched with MeOH, stirred at r.t. for 5 min, and concentrated *in vacuo*. The crude residue was purified by flash column chromatography (SiO_2, loading in CH_2Cl_2; gradient elution: 100:0 CH_2Cl_2/MeOH to 98:2 CH_2Cl_2/MeOH) to yield the title compound as a white powder (213 mg, 97%).

m.p. (CH_2Cl_2): 69 - 70 °C.

R*f*: 0.80 (SiO_2, 90:10 CH_2Cl_2/MeOH).

^{1}H NMR (400 MHz, $CDCl_3$) δ_H: 7.24 (4H, dd, *J* = 8.2, 5.3 Hz, *H*3), 7.04 (4H, t, *J* = 8.2 Hz, *H*2), 5.41 (1H, br t, *J* = 5.0 Hz, N_A*H*), 4.90 (4H, s, *H*5), 3.59 (2H, td, *J* = 7.2, 5.0 Hz, *H*7), 1.40 (2H, quint, *J* = 7.5 Hz, *H*8), 1.13 (2H, sext, *J* = 7.3 Hz, *H*9), 0.82 (3H, t, *J* = 7.3 Hz, *H*10).

^{13}C NMR (101 MHz, $CDCl_3$) δ_C: 183.0 (s, *C*6), 162.5 (d, *J* = 246.7 Hz, *C*1), 131.7 (d, *J* = 3.1 Hz, *C*4), 128.8 (d, *J* = 8.1 Hz, *C*3), 116.1 (d, *J* = 21.6 Hz, *C*2), 53.4 (s, *C*5), 46.4 (s, *C*7), 31.1 (s, *C*8), 20.0 (s, *C*9), 13.8 (s, *C*10).

^{19}F NMR (565 MHz, $CDCl_3$) δ_F: –116.32 (quint, *J* = 7.7 Hz).

IR (film): ν_{MAX} = 3290 (N–H, br), 2958 (C–H, m), 2931 (C–H, m), 2872 (C–H, m), 1725 (C=S, s), 1605 (m), 1527 (N–H, s), 1508 (s), 1223 (C–F, m) cm^{-1}.

HRMS (ESI, positive ion mode): *m/z* calcd for $[C_{19}H_{22}F_2N_2S+H]^+$ = 349.1545. Found 349.1532.

***N*[1],*N*[2]-Bis(4-fluorobenzyl)ethane-1,2-diamine (9)**

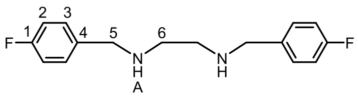

To a stirred solution of ethylene diamine (0.33 mL, 4.9 mmol, 1.0 eq.) in MeOH (100 mL) was added 4-fluorobenzaldehyde (1.07 mL, 10 mmol, 2.0 eq.) under air. The reaction mixture was stirred at r.t. for 24 h. The reaction mixture was then cooled to 0 °C and $NaBH_4$ (416 mg, 11 mmol, 2.2 eq.) was added portionwise. The reaction mixture was warmed to r.t. and stirred for 3 h. The reaction mixture was quenched with aqueous NaOH (1 M) and diluted with 3:1 $CHCl_3$/IPA. The organic layer was separated, and the aqueous layer was extracted three times with 3:1 $CHCl_3$/IPA. The organic extracts were combined, dried over $MgSO_4$, filtered, and concentrated *in vacuo*. The crude residue was purified by flash column chromatography (SiO_2, loading in CH_2Cl_2; gradient elution: 98.8:1.0:0.2 CH_2Cl_2/MeOH/[35% aqueous NH_3] to 90.4:8.0:1.6 CH_2Cl_2/MeOH/[35% aqueous NH_3]) to yield the title compound as a pale yellow liquid (1.07 g, 77%).

Data consistent with literature.[3]

^{1}H NMR (400 MHz, $CDCl_3$) δ_H: 7.26 (4H, dd, *J* = 8.7, 5.3 Hz, *H*3), 6.99 (4H, t, *J* = 8.7 Hz, *H*2), 3.73 (4H, s, *H*5), 2.74 (4H, s, *H*6), 1.56 (2H, s, N_A*H*).

^{13}C NMR (101 MHz, $CDCl_3$) δ_C: 162.0 (d, *J* = 244.3 Hz, *C*1), 136.3 (d, *J* = 3.2 Hz, *C*4), 129.7 (d, *J* = 8.0 Hz, *C*3), 115.3 (d, *J* = 21.2 Hz, *C*2), 53.3 (s, *C*5), 48.9 (s, *C*6).

^{19}F NMR (377 MHz, $CDCl_3$) δ_F: –118.32 (tt, *J* = 8.8, 5.4 Hz).

1,1'-(Ethane-1,2-diyl)bis(3-butyl-1-(4-fluorobenzyl)thiourea) (2)

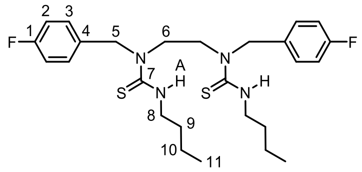

To a stirred solution of **9** (193 mg, 0.70 mmol, 1.0 eq.) in CH_2Cl_2 (22 mL) at 0 °C was added *n*-butyl isothiocyanate (0.18 mL, 1.5 mmol, 2.2 eq.) dropwise under air. The reaction mixture was warmed to r.t. and stirred for 22 h. The reaction mixture was quenched with MeOH, stirred at r.t. for 5 min, and concentrated *in vacuo*. The crude residue was purified by flash column chromatography (SiO_2, loading in CH_2Cl_2; gradient elution: 100:0 CH_2Cl_2/MeOH to 98:2 CH_2Cl_2/MeOH) to yield the title compound as a white powder (393 mg, quant.).

m.p. (CH_2Cl_2): 150 - 151 °C.

R*f*: 0.78 (SiO_2, 90:10 CH_2Cl_2/MeOH).

^{1}H NMR (600 MHz, $CDCl_3$) δ_H: 7.16 (4H, dd, *J* = 8.3, 5.3 Hz, *H*3), 7.02 (4H, t, *J* = 8.4 Hz, *H*2), 6.34 (2H, br s, N_AH) 4.77 (4H, br s, *H*5), 3.75 (4H, br s, *H*6), 3.60 (4H, q, *J* = 6.5 Hz, *H*8), 1.54 (4H, br s, *H*9), 1.27 (4H, sext, *J* = 7.6 Hz, *H*10), 0.89 (6H, t, *J* = 7.4 Hz, *H*11).

^{13}C NMR (151 MHz, $CDCl_3$) δ_C: 182.3 (s, *C*7), 162.6 (d, *J* = 247.1 Hz, *C*1), 131.7 (s, *C*4), 128.7 (br s, *C*3), 116.2 (d, *J* = 21.7 Hz, *C*2), 54.4 (br s, *C*5), 48.1 (br s, *C*6), 46.6 (s, *C*8), 31.2 (s, *C*9), 20.2 (s, *C*10), 13.9 (s, *C*11).

^{19}F NMR (565 MHz, CDCl3) δ_F: –116.01 (br s).

IR (film): v_{MAX} = 3349 (N–H, br), 2930 (C–H, m), 2874 (C–H, m), 1725 (C=S, s), 1510 (N–H, m), 1265 (C–F, s) cm^{-1}.

HRMS (ESI, positive ion mode): *m/z* calcd for $[C_{26}H_{36}F_2N_4S_2+H]^+$ = 507.2422. Found 507.2399.

***N,N'*-(((Butylcarbamothioyl)azanediyl)bis(ethane-2,1-diyl))bis(2,2,2-trifluoroacetamide) (13)**

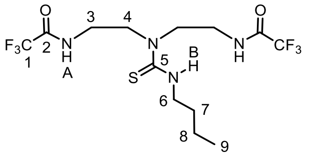

To a stirred solution of diethylenetriamine (0.65 mL, 6.0 mmol, 1.0 eq.) in CH_2Cl_2 (80 mL) at 0 °C was added ethyl trifluoroacetate (1.60 mL, 13 mmol, 2.2 eq.) dropwise under air. The reaction mixture was warmed to r.t. and stirred for 2 h. The reaction mixture was then cooled to 0 °C and *n*-butyl isothiocyanate (0.80 mL, 6.6 mmol, 1.1 eq.) was added dropwise. The reaction mixture was stirred at 0 °C for 5 min, warmed to r.t., and stirred for 21 h. The reaction mixture was quenched with MeOH, stirred at r.t. for 5 min, and concentrated *in vacuo*. The crude residue was purified by flash column chromatography (SiO_2, loading in CH_2Cl_2; gradient elution: 98:2 CH_2Cl_2/MeOH to 95:5 CH_2Cl_2/MeOH) to yield the title compound as a white powder (1.72 g, 70%).

m.p. (CH_2Cl_2): 94 - 95 °C.

R*f*: 0.50 (SiO_2, 90:10 CH_2Cl_2/MeOH).

^{1}H NMR (600 MHz, $CDCl_3$) δ_H: 7.69 (2H, br s, NA*H*), 7.06 (1H, brt, *J* = 5.0 Hz, NB*H*), 3.90 (4H, t, *J* = 6.9 Hz, *H*4), 3.67 (2H, td, *J* = 7.4, 5.0 Hz, *H*6), 3.55 (4H, q, *J* = 6.5 Hz, *H*3), 1.68 (2H, quint, *J* = 7.4 Hz, *H*7), 1.39 (2H, sext, *J* = 7.4 Hz, *H*8), 0.95 (3H, t, *J* = 7.4 Hz, *H*9).

^{13}C NMR (151 MHz, $CDCl_3$) δ_C: 183.0 (s, *C*5), 159.0 (q, *J* = 37.9 Hz, *C*2), 115.7 (q, *J* = 287.0 Hz, *C*1), 48.2 (s, *C*4), 47.2 (s, *C*6), 38.8 (s, *C*3), 30.8 (s, *C*7), 20.3 (s, *C*8), 13.9 (s, *C*9).

^{19}F NMR (565 MHz, $CDCl_3$) δ_F: –75.83 (s).

IR (film): ν_{MAX} = 3312 (N–H, br), 3069 (br), 2937 (C–H, m), 2882 (C–H, m), 1707 (C=O, s), 1546 (N–H, s), 1264 (s), 1179 (C–F, s) cm^{-1}.

HRMS (ESI, positive ion mode): *m/z* calcd for $[C_{13}H_{20}F_6N_4O_2S+H]^+$ = 411.1284. Found 411.1273.

1,1-Bis(2-aminoethyl)-3-butylthiourea (16)

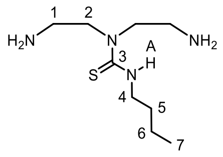

To a stirred solution of **13** (399 mg, 0.97 mmol, 1.0 eq.) in THF (5.0 mL) and EtOH (10 mL) was added NaOH (312 mg, 7.8 mmol, 8.0 eq.) in H_2O (5.0 mL) under air. The reaction mixture was stirred at r.t. for 21 h and then concentrated *in vacuo*. The resulting mixture was diluted with 3:1 $CHCl_3$/IPA and H_2O, and the organic layer was separated. The aqueous layer was extracted three times with 3:1 $CHCl_3$/IPA. The organic extracts were combined, dried over $MgSO_4$, filtered, and concentrated *in vacuo* to yield the title compound as a light yellow oil (200 mg, 94%).

R_f: 0.05 (SiO_2, 90:10 CH_2Cl_2/MeOH).

^{1}H NMR (600 MHz, $CDCl_3$) δ_H: 8.92 (1H, br t, J = 4.7 Hz, N_AH), 3.68 (4H, t, J = 6.0 Hz, *H*2), 3.56 (2H, td, J = 7.2, 4.7 Hz, *H*4), 2.99 (4H, t, J = 6.0 Hz, *H*1), 1.58 (2H, quint, J = 7.2 Hz, *H*5), 1.38 (2H, sext, J = 7.6 Hz, *H*6), 0.93 (3H, t, J = 7.4 Hz, *H*7).

^{13}C NMR (151 MHz, $CDCl_3$) δ_C: 184.8 (s, *C*3), 54.9 (s, *C*2), 46.1 (s, *C*4), 40.7 (s, *C*1), 31.4 (s, *C*5), 20.5 (s, *C*6), 14.0 (s, *C*7).

IR (film): ν_{MAX} = 3309 (N–H, br), 2934 (C–H, m), 2882 (C–H, m), 1726 (C=O, s), 1682 (m), 1543 (N–H, w), 1275 (s) cm^{-1}.

HRMS (ESI, positive ion mode): *m/z* calcd for $[C_9H_{22}N_4S+H]^+$ = 219.1633. Found 219.1638.

3-Butyl-1,1-bis(2-((4-fluorobenzyl)amino)ethyl)thiourea (19)

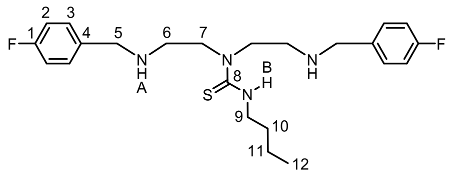

To a stirred solution of **16** (419 mg, 1.9 mmol, 1.0 eq.) in MeOH (38 mL) was added 4-fluorobenzaldehyde (0.41 mL, 3.8 mmol, 2.0 eq.) under air. The reaction mixture was stirred at r.t. for 20 h. The reaction mixture was then cooled to 0 °C and $NaBH_4$ (159 mg, 4.2 mmol, 2.2 eq.) was added portionwise. The reaction mixture was warmed to r.t. and stirred for 5 h. The reaction mixture was quenched with aqueous NaOH (1 M) and diluted with 3:1 $CHCl_3$/IPA. The organic layer was separated, and the aqueous layer was extracted three times with 3:1 $CHCl_3$/IPA. The organic extracts were combined, dried over $MgSO_4$, filtered, and concentrated *in vacuo*. The crude residue was purified by flash column chromatography (SiO_2, loading in CH_2Cl_2; gradient elution: 98.8:1.0:0.2 CH_2Cl_2/MeOH/[35% aqueous NH_3] to 94.0:5.0:1.0 CH_2Cl_2/MeOH/[35% aqueous NH_3]) to yield the title compound as a pale yellow oil (438 mg, 53%).

R*f*: 0.50 (SiO_2, 88:10:2 CH_2Cl_2/MeOH/[35% aqueous NH_3]).

^{1}H NMR (400 MHz, $CDCl_3$) δ_H: 8.63 (1H, br t, *J* = 4.9 Hz, N_BH), 7.21 (4H, dd, *J* = 8.3, 5.3 Hz, *H*3), 7.00 (4H, t, *J* = 8.6 Hz, *H*2), 3.73 (4H, s, *H*5), 3.70 (4H, t, *J* = 6.5 Hz, *H*7), 3.52 (2H, td, *J* = 7.3, 4.8 Hz, *H*9), 2.87 (4H, t, *J* = 5.8 Hz, *H*6), 1.53 – 1.41 (2H, quint, *J* = 7.5 Hz, *H*10), 1.31 (2H, sext, *J* = 7.3 Hz, *H*11), 0.87 (3H, t, *J* = 7.3 Hz, *H*12).

^{13}C NMR (101 MHz, $CDCl_3$) δ_C: 184.3 (s, *C*8), 162.1 (d, *J* = 245.2 Hz, *C*1), 135.4 (d, *J* = 2.7 Hz, *C*4), 129.8 (d, *J* = 7.9 Hz, *C*3), 115.4 (d, *J* = 21.4 Hz, *C*2), 53.3 (s, *C*5), 52.6 (s, *C*7), 47.9 (s, *C*6), 46.0 (s, *C*9), 31.4 (s, *C*10), 20.4 (s, *C*11), 14.0 (s, *C*12).

^{19}F NMR (377 MHz, $CDCl_3$) δ_F: –115.37 (s).

IR (film): ν_{MAX} = 3245 (N–H, br), 2958 (C–H, s), 2930 (C–H, s), 2870 (C–H, m), 1726 (C=S, m), 1509 (N–H, m), 1223 (C–F, s) cm^{-1}.

HRMS (ESI, positive ion mode): *m/z* calcd for $[C_{23}H_{32}F_2N_4S+H]^+$ = 435.2389. Found 435.2408.

3-Butyl-1,1-bis(2-(3-butyl-1-(4-fluorobenzyl)thioureido)ethyl)thiourea (3)

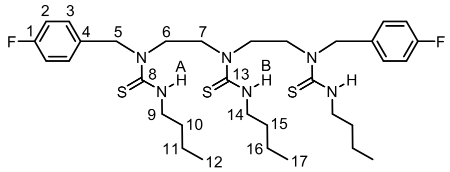

To a stirred solution of **19** (332 mg, 0.76 mmol, 1.0 eq.) in CH_2Cl_2 (24 mL) at 0 °C was added *n*-butyl isothiocyanate (0.20 mL, 1.7 mmol, 2.2 eq.) dropwise under air. The reaction mixture was warmed to r.t. and stirred for 22 h. The reaction mixture was quenched with MeOH, stirred at r.t. for 5 min, and concentrated *in vacuo*. The crude residue was purified by flash column chromatography (SiO_2, loading in CH_2Cl_2; gradient elution: 100:0 CH_2Cl_2/MeOH to 90:10 CH_2Cl_2/MeOH) to yield the title compound as a white powder (359 mg, 71%).

m.p. (CH_2Cl_2): 134 - 135 °C.

R*f*: 0.74 (SiO_2, 90:10 CH_2Cl_2/MeOH).

^{1}H NMR (600 MHz, *d*6-DMSO) δ_H: 7.86 (1H, br t, *J* = 5.2 Hz, N_B*H*), 7.73 (2H, br s, N_A*H*), 7.23 (4H, br s, *H*3), 7.16 (4H, t, *J* = 8.7 Hz, *H*2), 4.89 (4H, br s, *H*5), 3.71 (8H, br s, *H*6 and *H*7), 3.48 (6H, q, *J* = 5.4 Hz, *H*9 and *H*14), 1.51 (6H, quint, *J* = 7.7 Hz, *H*10 and *H*15), 1.24 (2H, sext, *J* = 7.4 Hz, *H*16), 1.20 (4H, br s, *H*11), 0.86 (6H, t, *J* = 7.5 Hz, *H*12), 0.83 (3H, t, *J* = 7.9 Hz, *H*17).

^{13}C NMR (151 MHz, *d*6-DMSO) δ_C: 190.0 (br s, *C*8), 180.6 (s, *C*13), 161.4 (d, *J* = 243.0 Hz, *C*1), 133.2 (br s, *C*4), 128.8 (s, *C*3), 115.2 (d, *J* = 21.2 Hz, *C*2), 52.2 (br s, *C*5), 48.5 (br s, *C*6 and *C*7), 45.3 (s, *C*9), 45.3 (s, *C*14), 30.7 (s, *C*10), 30.7 (s, *C*15), 19.7 (s, *C*16), 19.5 (s, *C*11), 13.8 (s, *C*12 and *C*17).

^{19}F NMR (565 MHz, $CDCl_3$) δ_F: –114.80 (br s), –116.45 (br s).

IR (film): ν_{MAX} = 3245 (N–H, br), 3049 (br), 2962 (C–H, s), 2930 (C–H, s), 2870 (C–H, m), 1726 (C=S, m), 1543 (N–H, s), 1509 (s), 1362 (m), 1223 (C–F, s) cm^{-1}.

HRMS (ESI, positive ion mode): *m/z* calcd for $[C_{33}H_{50}F_2N_6S_3+H]^+$ = 665.3300. Found 665.3281.

***N*,*N'*-((6,11-Dithioxo-5,7,10,12-tetraazahexadecane-7,10-diyl)bis(ethane-2,1-diyl))bis(2,2,2-trifluoroacetamide) (14)**

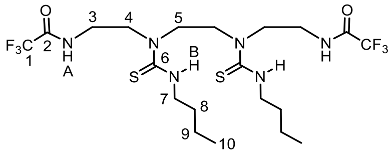

To a stirred solution of triethylenetetramine (0.60 mL, 4.0 mmol, 1.0 eq.) in CH_2Cl_2 (60 mL) at 0 °C was added ethyl trifluoroacetate (1.00 mL, 8.4 mmol, 2.1 eq.) dropwise under air. The reaction mixture was warmed to r.t. and stirred for 19 h. The reaction mixture was then cooled to 0 °C and *n*-butyl isothiocyanate (1.10 mL, 8.8 mmol, 2.2 eq.) was added dropwise. The reaction mixture was stirred at 0 °C for 5 min, warmed to r.t., and stirred for 6 h. The reaction mixture was quenched with MeOH, stirred at r.t. for 5 min, and concentrated *in vacuo*. The crude residue was purified by flash column chromatography (SiO_2, dry-loading on SiO_2; gradient elution: 99:1 CH_2Cl_2/MeOH to 95:5 CH_2Cl_2/MeOH) to yield the title compound as a white foam (1.93 g, 85%).

m.p. (CH_2Cl_2): 130 - 132 °C.

R*f*: 0.37 (SiO_2, 90:10 CH_2Cl_2/MeOH).

^{1}H NMR (400 MHz, $CDCl_3$) δ_H: 7.99 (2H, br s, N_AH), 7.47 (2H, br s, N_BH), 3.78 (8H, br s, *H*4 and *H*5), 3.62 (4H, td, *J* = 7.5, 4.9 Hz, *H*7), 3.52 (4H, q, *J* = 6.5 Hz, *H*3), 1.66 (4H, quint, *J* = 7.7 Hz, *H*8), 1.38 (4H, sext, *J* = 7.4 Hz, *H*9), 0.93 (6H, t, *J* = 7.3 Hz, *H*10).

^{13}C NMR (101 MHz, $CDCl_3$) δ_C: 182.0 (s, *C*6), 159.0 (q, *J* = 36.4 Hz, *C*2), 115.7 (q, *J* = 286.9 Hz, *C*1), 48.4 (br s, *C*4 and *C*5), 46.9 (s, *C*7), 38.8 (br s, *C*3), 30.9 (s, *C*8), 20.3 (s, *C*9), 13.9 (s, *C*10).

^{19}F NMR (377 MHz, $CDCl_3$) δ_F: –75.79 (s).

IR (film): ν_{MAX} = 3234 (N–H, br), 3069 (br), 2965 (C–H, s), 2934 (C–H, s), 2878 (C–H, m), 1707 (C=S and C=O, m), 1551 (N–H, m), 1172 (C–F, s) cm^{-1}.

HRMS (ESI, positive ion mode): *m/z* calcd for $[C_{20}H_{34}F_6N_6O_2S_2+H]^+$ = 569.2162. Found 569.2161.

1,1'-(Ethane-1,2-diyl)bis(1-(2-aminoethyl)-3-butylthiourea) (17)

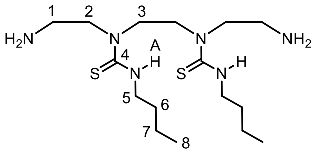

To a stirred solution of **14** (1.63 g, 2.9 mmol, 1.0 eq.) in THF (15 mL) and EtOH (30 mL) was added NaOH (920 mg, 23 mmol, 8.0 eq.) in H_2O (15 mL) under air. The reaction mixture was stirred at r.t. for 21 h and then concentrated *in vacuo*. The resulting mixture was diluted with 3:1 $CHCl_3$/IPA and H_2O, and the organic layer was separated. The aqueous layer was extracted three times with 3:1 $CHCl_3$/IPA. The organic extracts were combined, dried over $MgSO_4$, filtered, and concentrated *in vacuo* to yield the title compound as a yellow powder (822 mg, 75%).

m.p. (CH_2Cl_2): 132 - 134 °C.

R*f*: 0.08 (SiO_2, 90:10 CH_2Cl_2/MeOH).

^{1}H NMR (400 MHz, CD_3OD) δ_H: 3.81 (8H, s, *H*2 and *H*3), 3.59 (4H, t, *J* = 7.2 Hz, *H*5), 3.01 (4H, t, *J* = 6.5 Hz, *H*1), 1.64 (4H, quint, *J* = 7.1 Hz, *H*6), 1.39 (4H, sext, *J* = 7.3 Hz, *H7*), 0.96 (6H, t, *J* = 7.4 Hz, *H*8).

^{13}C NMR (101 MHz, CD_3OD) δ_C: 183.3 (s, *C*4), 52.6 (s, *C*2 and *C*3), 47.0 (s, *C*5), 40.0 (s, *C*1), 32.3 (s, *C*6), 21.3 (s, *C*7), 14.2 (s, *C*8).

IR (film): ν_{MAX} = 3229 (N–H, br), 3057 (br), 2958 (C–H, s), 2934 (C–H, s), 2870 (C–H, m), 1682 (s), 1554 (N–H, m), 1391 (s) cm^{-1}.

HRMS (ESI, positive ion mode): *m/z* calcd for $[C_{16}H_{36}N_6S_2+H]^+$ = 377.2516. Found 377.2532.

1,1'-(Ethane-1,2-diyl)bis(3-butyl-1-(2-((4-fluorobenzyl)amino)ethyl)thiourea) (20)

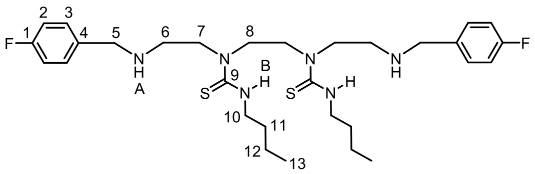

To a stirred solution of **17** (715 mg, 1.9 mmol, 1.0 eq.) in MeOH (38 mL) was added 4-fluorobenzaldehyde (0.41 mL, 3.8 mmol, 2.0 eq.) under air. The reaction mixture was stirred at r.t. for 23 h. The reaction mixture was then cooled to 0 °C and $NaBH_4$ (159 mg, 4.2 mmol, 2.2 eq.) was added portionwise. The reaction mixture was warmed to r.t. and stirred for 2 h. The reaction mixture was quenched with aqueous NaOH (1 M) and diluted with 3:1 $CHCl_3$/IPA. The organic layer was separated, and the aqueous layer was extracted three times with 3:1 $CHCl_3$/IPA. The organic extracts were combined, dried over $MgSO_4$, filtered, and concentrated *in vacuo*. The crude residue was purified by flash column chromatography (SiO_2, loading in CH_2Cl_2; gradient elution: 98.8:1.0:0.2 CH_2Cl_2/MeOH/[35% aqueous NH_3] to 94.0:5.0:1.0 CH_2Cl_2/MeOH/[35% aqueous NH_3]) to yield the title compound as a colourless oil (270 mg, 24%).

R*f*: 0.42 (SiO_2, 88:10:2 CH_2Cl_2/MeOH/[35% aqueous NH_3]).

^{1}H NMR peak for N_B*H* could not be identified.

^{1}H NMR (400 MHz, $CDCl_3$) δ_H: 7.21 (4H, dd, *J* = 8.6, 5.3 Hz, *H*3), 6.99 (4H, t, *J* = 8.6 Hz, *H*2), 3.81 (4H, br s, *H*7), 3.73 (4H, s, *H*5), 3.63 (4H, br s, *H*8), 3.51 (4H, br s, *H*10), 2.85 (4H, br s, *H*6), 1.70 (2H, br s, N_A*H*), 1.50 (4H, br s, *H*11), 1.32 (4H, sext, *J* = 7.3 Hz, *H*12), 0.88 (6H, t, *J* = 7.4 Hz, *H*13).

^{13}C NMR (126 MHz, $CDCl_3$) δ_C: 183.3 (br s, *C*9), 162.2 (d, *J* = 245.5 Hz, *C*1), 135.0 (s, *C*4), 129.8 (d, *J* = 7.9 Hz, *C*3), 115.5 (d, *J* = 21.3 Hz, *C*2), 53.5 (s, *C*5), 52.8 (br s, *C*7 and *C*8), 48.3 (s, *C*6), 46.2 (s, *C*10), 31.3 (s, *C*11), 20.4 (s, *C*12), 14.0 (s, *C*13).

^{19}F NMR (376 MHz, CDCl3) δ_F: –115.00 (br s).

IR (film): ν_{MAX} = 3228 (N–H, br), 3045 (br), 2962 (C–H, s), 2930 (C–H, s), 2862 (C–H, m), 1725 (C=S, w), 1538 (N–H, m), 1509 (s), 1360 (m), 1221 (C–F, s) cm^{-1}.

HRMS (ESI, positive ion mode): *m/z* calcd for $[C_{30}H_{46}F_2N_6S_2+H]^+$ = 593.3266. Found 593.3373.

1,1'-(Ethane-1,2-diyl)bis(3-butyl-1-(2-(3-butyl-1-(4-fluorobenzyl)thioureido)ethyl)thiourea) (4)

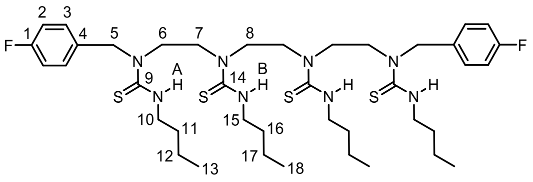

To a stirred solution of **20** (215 mg, 0.36 mmol, 1.0 eq.) in CH_2Cl_2 (11 mL) at 0 °C was added *n*-butyl isothiocyanate (0.10 mL, 0.79 mmol, 2.2 eq.) dropwise under air. The reaction mixture was warmed to r.t. and stirred for 18 h. The reaction mixture was quenched with MeOH, stirred at r.t. for 5 min, and concentrated *in vacuo*. The crude residue was purified by flash column chromatography (SiO_2, loading in CH_2Cl_2; gradient elution: 100:0 CH_2Cl_2/MeOH to 90:10 CH_2Cl_2/MeOH) to yield the title compound as a white wax (155 mg, 52%).

R_f: 0.83 (SiO_2, 90:10 CH_2Cl_2/MeOH).

^{1}H NMR (600 MHz, d_6-DMSO) δ_H: 7.82 (2H, br s, N_AH or N_BH), 7.73 (2H, br s, N_AH or N_BH), 7.22 (4H, br s, *H*3), 7.17 (4H, brt, *J* = 8.7 Hz, *H*2), 4.89 (4H, br s, *H*5), 3.73 (12H, br s, *H*6, *H*7, and *H*8), 3.48 (8H, q, *J* = 5.4 Hz, *H*10 and *H*15), 1.53 (4H, quint, *J* = 7.5 Hz, *H*16), 1.48 (4H, br s, *H*11), 1.29 (4H, sext, *J* = 7.5 Hz, *H*17), 1.19 (4H, br s, *H*12), 0.85 (6H, t, *J* = 7.5 Hz, *H*18), 0.83 (6H, brt, *J* = 7.5 Hz, *H*13).

^{13}C NMR (151 MHz, d_6-DMSO) δ_C: 181.0 (br s, *C*9), 180.6 (s, *C*14), 161.4 (d, *J* = 243.0 Hz, *C*1), 133.1 (br s, *C*4), 128.7 (br s, *C*3), 115.2 (d, *J* = 21.3 Hz, *C*2), 51.8 (br s, *C*5), 48.5 (br s, *C*6 and/or *C*7 and/or *C*8), 46.8 (br s, *C*6 and/or *C*7 and/or *C*8), 45.3 (s, *C*10 and *C*15), 30.7 (s, *C*11 or *C*16), 30.7 (s, *C*11 or *C*16), 19.7 (s, *C*17), 19.5 (br s, *C*12), 13.8 (s, *C*13 and *C*18).

^{19}F NMR (565 MHz, $CDCl_3$) δ_F: –115.17 (br s), –116.45 (br s).

IR (film): ν_{MAX} = 3229 (N–H, br), 3049 (br), 2958 (C–H, s), 2929 (C–H, s), 2866 (C–H, m), 1726 (C=S, s), 1550 (N–H, m), 1264 (C–F, s) cm^{-1}.

HRMS (ESI, positive ion mode): *m/z* calcd for $[C_{40}H_{64}F_2N_8S_4+H]^+$ = 823.4178. Found 823.4162.

3-Butyl-1,1-bis(2-(3-butyl-1-(2-((4-fluorobenzyl)amino)ethyl)thioureido)ethyl)thiourea (21)

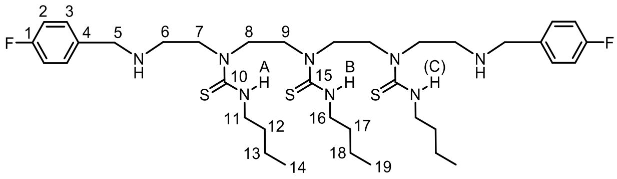

Step 1 *di-trifluoroacetylation and tri-thiourea formation*: To a stirred solution of tetraethylenepentamine (0.38 mL, 2.0 mmol, 1.0 eq.) in CH_2Cl_2 (30 mL) at 0 °C was added ethyl trifluoroacetate (0.52 mL, 4.4 mmol, 2.2 eq.) dropwise under air. The reaction mixture was warmed to r.t. and stirred for 4 h. The reaction mixture was then cooled to 0 °C and *n*-butyl isothiocyanate (0.80 mL, 6.6 mmol, 3.3 eq.) was added dropwise. The reaction mixture was stirred at 0 °C for 5 min, warmed to r.t., and then stirred for 19 h. The reaction mixture was quenched with MeOH, stirred at r.t. for 5 min, and concentrated *in vacuo*. The crude residue was purified by flash column chromatography (SiO_2, loading in CH_2Cl_2; gradient elution: 0:100 MeOH/CH_2Cl_2 to 10:90 MeOH/CH_2Cl_2) to yield the intermediate di-protected tri-thiourea (**15**) as a light yellow foam (1.04 g, 1.4 mmol).

Step 2 *di-trifluoroacetamide deprotection*: To a stirred solution of the material from **step 1** (1.04 g, 1.4 mmol, 1.0 eq.) in THF (8.0 mL) and EtOH (16 mL) was added NaOH (448 mg, 11 mmol, 7.9 eq.) in H_2O (8.0 mL) under air. The reaction mixture was stirred at r.t. for 19 h and then concentrated *in vacuo*. The resulting mixture was diluted with 3:1 $CHCl_3$/IPA and H_2O, and the organic layer was separated. The aqueous layer was extracted three times with 3:1 CHCl3/IPA. The organic extracts were combined, dried over $MgSO_4$, filtered, and concentrated *in vacuo* to yield the intermediate tri-thiourea diamine (**18**) as a yellow oil (616 mg, 1.2 mmol).

Step 3 *installation of capping groups*: To a stirred solution of the material from **step 2** (616 mg, 1.2 mmol, 1.0 eq.) in MeOH (23 mL) was added 4-fluorobenzaldehyde (0.25 mL, 2.3 mmol, 1.9 eq.) under air. The reaction mixture was stirred at r.t. for 21 h. The reaction mixture was then cooled to 0 °C and $NaBH_4$ (96 mg, 2.5 mmol, 2.1 eq.) was added portionwise. The reaction mixture was warmed to r.t. and stirred for 4 h. The reaction mixture was quenched with aqueous NaOH (1 M) and diluted with 3:1 $CHCl_3$/IPA. The organic layer was separated, and the aqueous layer was extracted three times with 3:1 $CHCl_3$/IPA. The organic extracts were combined, dried over $MgSO_4$, filtered, and concentrated *in vacuo*. The crude residue was purified by flash column chromatography (SiO_2, loading in CH_2Cl_2; gradient elution: 98.8:1.0:0.2 CH_2Cl_2/MeOH/[35% aqueous NH_3] to 94.0:5.0:1.0 CH_2Cl_2/MeOH/[35% aqueous NH_3]) to yield the title compound as a colourless oil (107 mg, 7% over three steps).

R_f: 0.50 (SiO_2, 88:10:2 CH_2Cl_2/MeOH/[35% aqueous NH_3]).

N_AH, N_BH, and N_CH are distinguishable in ^1H NMR at 25 °C.

^{1}H NMR (600 MHz, $CDCl_3$) δ_H: 8.98 (1H, br s, N_AH or N_BH), 8.77 (1H, br s, N_AH or N_BH), 7.81 (1H, br s, N_CH), 7.20 (4H, t, *J* = 8.3 Hz, *H*3), 7.02 (4H, t, *J* = 8.5 Hz, *H*2), 4.21 - 3.77 (8H, br m, *H*8 and *H*9), 3.73 (4H, s, *H*5), 3.63 (2H, td, *J* = 7.4, 4.9 Hz, *H*16), 3.49 (8H, br s, *H*7 and *H*11), 2.91 (4H, br t, *J* = 4.5 Hz, *H*6), 1.66 (2H, quint, *J* = 7.1 Hz, *H*17), 1.44 (4H, br s, *H*12), 1.38 (2H, sext, *J* = 7.5 Hz, *H*18), 1.31 (4H, sext, *J* = 7.3 Hz, *H*13), 0.93 (3H, t, *J* = 7.2 Hz, *H*19), 0.88 (6H, t, *J* = 7.3 Hz, *H*14).

^{13}C NMR (151 MHz, $CDCl_3$) δ_C: 184.0 (br s, *C*10), 181.3 (s, *C*15), 162.3 (d, *J* = 246.1 Hz, *C*1), 134.6 (br s, *C*4), 129.9 (d, *J* = 8.0 Hz, *C*3), 115.7 (d, *J* = 21.6 Hz, *C*2), 53.6 (s, *C*5), 52.8 (br s, *C*7), 49.9 (br s, *C*8 or *C*9), 49.6 (br s, *C*8 or *C*9), 48.6 (s, *C*6), 46.6 (s, *C*16), 46.0 (br s, *C*11), 31.4 (br s, *C*12), 31.2 (s, *C*17), 20.5 (s, *C*18), 20.5 (br s, *C*13), 14.0 (s, *C*19), 14.0 (br s, *C*14).

^{19}F NMR (377 MHz, $CDCl_3$) δ_F: –114.93 (br s), –114.71 (br s).

IR (film): ν_{MAX} = 3232 (N–H, br), 2958 (C–H, s), 2930 (C–H, s), 2874 (C–H, m), 1726 (C=S, m), 1555 (N–H, m), 1510 (s), 1224 (C–F, s) cm^{-1}.

HRMS (ESI, positive ion mode): *m/z* calcd for $[C_{37}H_{60}F_2N_8S_3+H]^+$ = 751.4144. Found 751.4115.

1-(3,6-Bis(butylcarbamothioyl)-9-(4-fluorobenzyl)-10-thioxo-3,6,9,11-tetraazapentadecyl)-3-butyl-1-(2-(3-butyl-1-(4-fluorobenzyl)thioureido)ethyl)thiourea (5)

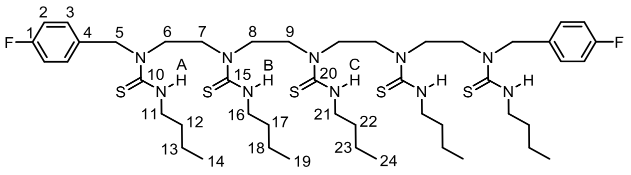

To a stirred solution of **21** (95 mg, 0.13 mmol, 1.0 eq.) in CH_2Cl_2 (15 mL) at 0 °C was added *n*-butyl isothiocyanate (35 µL, 0.29 mmol, 2.2 eq.) dropwise under air. The reaction mixture was warmed to r.t. and stirred for 4 h. The reaction mixture was quenched with MeOH, stirred at r.t. for 5 min, and concentrated *in vacuo*. The crude residue was purified by flash column chromatography (SiO_2, loading in CH_2Cl_2; gradient elution: 100:0 CH_2Cl_2/MeOH to 90:10 CH_2Cl_2/MeOH) to yield the title compound as a white powder (130 mg, 98%).

m.p. (CH_2Cl_2): 100 - 102 °C.

R_f: 0.68 (SiO_2, 90:10 CH_2Cl_2/MeOH).

^{1}H NMR (600 MHz, d_6-DMSO) δ_H: 7.85 (3H, br s, N_BH and N_CH), 7.73 (4H, br s, *H*3), 7.21 (2H, br s, N_AH), 7.16 (4H, t, *J* = 8.6 Hz, *H*2), 4.88 (4H, br s, *H*5), 3.74 (16H, br s, *H*6, *H*7, *H*8, and *H*9), 3.52 – 3.43 (10H, m, *H*11, *H*16, and *H*21), 1.53 (6H, quint, *J* = 7.3 Hz, *H*17 and *H*22), 1.48 (4H, br s, *H*12), 1.28 (6H, sext, *J* = 7.2 Hz, *H*18 and *H*23), 1.18 (4H, br s, *H*13), 0.89 (3H, t, *J* = 7.0 Hz, *H*24), 0.87 (6H, t, *J* = 7.3 Hz, *H*19), 0.83 (6H, br t, *J* = 6.9 Hz, *H*14).

^{13}C NMR (151 MHz, d_6-DMSO) δ_C: 180.9 (br s, *C*10), 180.6 (s, *C*15 and *C*20), 161.4 (d, *J* = 243.0 Hz, *C*1), 133.1 (br s, *C*4), 128.7 (br s, *C*3), 115.2 (d, *J* = 21.7 Hz, *C*2), 52.5 (br s, *C*5), 49.0 (br s, *C*6, *C*7, *C*8, and *C*9), 45.4 (s, *C*11), 45.3 (s, *C*16 and *C*21), 30.7 (s, *C*12 or *C*17), 30.7 (s, *C*12 or *C*17), 30.6 (s, *C*22), 19.7 (s, *C*23), 19.7 (s, *C*18), 19.5 (br s, *C*13), 13.8 (s, *C*19 and *C*24), 13.7 (s, *C*14).

^{19}F NMR (565 MHz, $CDCl_3$) δ_F: –115.12 (br s), –115.52 (br s), –116.08 (br s).

IR (film): ν_{MAX} = 3228 (N–H, br), 3049 (br), 2957 (C–H, s), 2934 (C–H, s), 2870 (C–H, m), 1726 (C=S, m), 1551 (N–H, m), 1509 (s), 1224 (C–F, s) cm^{-1}.

HRMS (ESI, positive ion mode): *m/z* calcd for $[C_{47}H_{78}F_2N_{10}S_5+H]^+$ = 981.5055. Found 981.5027.

N^1,N^2-Bis(2-fluorobenzyl)ethane-1,2-diamine (28)

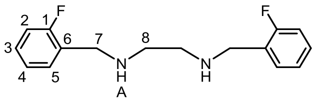

To a stirred solution of ethylene diamine (0.33 mL, 4.9 mmol, 1.0 eq.) in MeOH (100 mL) was added 2-fluorobenzaldehyde (1.05 mL, 10 mmol, 2.0 eq.) under air. The reaction mixture was stirred at r.t. for 24 h. The reaction mixture was then cooled to 0 °C and $NaBH_4$ (416 mg, 11 mmol, 2.2 eq.) was added portionwise. The reaction mixture was warmed to r.t. and stirred for 3 h. The reaction mixture was quenched with aqueous NaOH (1 M) and diluted with 3:1 $CHCl_3$/IPA. The organic layer was separated, and the aqueous layer was extracted three times with 3:1 $CHCl_3$/IPA. The organic extracts were combined, dried over $MgSO_4$, filtered, and concentrated *in vacuo*. The crude residue was purified by flash column chromatography (SiO_2, loading in CH_2Cl_2; gradient elution: 98.8:1.0:0.2 CH2Cl_2/MeOH/[35% aqueous NH_3] to 90.4:8.0:1.6 CH_2Cl_2/MeOH/[35% aqueous NH3]) to yield the title compound as a pale yellow liquid (807 mg, 58%).

Data consistent with literature.[3]

^{1}H NMR (400 MHz, $CDCl_3$) δ_H: 7.32 (2H, td, *J* = 7.5, 1.8 Hz, *H*3), 7.22 (2H, tdd, *J* = 7.5, 5.2, 1.9 Hz, *H*2), 7.09 (2H, td, *J* = 7.5, 1.2 Hz, *H*4), 7.02 (2H, ddd, *J* = 10.2, 8.1, 1.2 Hz, *H*5), 3.83 (4H, s, *H*7), 2.75 (4H, s, *H*8), 1.69 (2H, s, N_A*H*).

^{13}C NMR (101 MHz, $CDCl_3$) δ_C: 161.3 (d, *J* = 245.4 Hz, *C*1), 130.5 (d, *J* = 5.0 Hz, *C*3), 128.7 (d, *J* = 8.2 Hz, *C*2), 127.4 (d, *J* = 15.0 Hz, *C*6), 124.2 (d, *J* = 3.7 Hz, *C*4), 115.4 (d, *J* = 22.0 Hz, *C*5), 48.6 (s, *C*8), 47.3 (d, *J* = 3.0 Hz, *C*7).

^{19}F NMR (377 MHz, $CDCl_3$) δ_F: –121.61- –121.67 (m).

1,1'-(Ethane-1,2-diyl)bis(3-butyl-1-(2-fluorobenzyl)thiourea) (22)

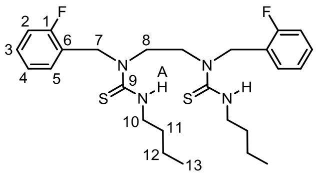

To a stirred solution of **28** (193 mg, 0.70 mmol, 1.0 eq.) in CH_2Cl_2 (22 mL) at 0 °C was added *n*-butyl isothiocyanate (0.18 mL, 1.5 mmol, 2.2 eq.) dropwise under air. The reaction mixture was warmed to r.t. and stirred for 21 h. The reaction mixture was quenched with MeOH, stirred at r.t. for 5 min, and concentrated *in vacuo*. The crude residue was purified by flash column chromatography (SiO_2, loading in CH_2Cl_2; gradient elution: 100:0 CH_2Cl_2/MeOH to 98:2 CH_2Cl_2/MeOH) to yield the title compound as a white powder (321 mg, 90%).

m.p. (CH_2Cl_2): 134 - 136 °C.

R*f*: 0.79 (SiO_2, 90:10 CH_2Cl_2/MeOH).

^{1}H NMR peak for N_AH could not be identified.

^{1}H NMR (600 MHz, $CDCl_3$) δ_H: 7.30 - 7.23 (4H, m, *H*2 and *H*3), 7.11 (2H, td, *J* = 7.5, 1.1 Hz, *H*5), 7.05 (2H, t, *J* = 9.3 Hz, *H*4), 4.87 (4H, br s, *H*7), 3.78 (4H, br s, *H*8), 3.62 (4H, br q, *J* = 6.6 Hz, *H*10), 1.58 (4H, br quint, *J* = 7.8 Hz, *H*11), 1.31 (4H, br sext, *J* = 7.4 Hz, *H*12), 0.90 (6H, t, *J* = 7.4 Hz, *H*13).

^{13}C NMR (151 MHz, $CDCl_3$) δ_C: 182.3 (s, *C*9), 160.7 (d, *J* = 245.3 Hz, *C*1), 130.0 (br s, *C*3), 129.5 (br s, *C*6), 125.0 (d, *J* = 3.5 Hz, *C*5), 123.1 (br s, *C*4), 115.7 (d, *J* = 21.7 Hz, *C*2), 48.3 (br s, *C*7 and *C*8), 46.7 (s, *C*10), 31.2 (s, *C*11), 20.3 (s, *C*12), 13.9 (s, *C*13).

^{19}F NMR (565 MHz, $CDCl_3$) δ_F: –119.45 (br s), –122.06 (br s).

IR (film): ν_{MAX} = 3245 (N–H, br), 3057 (br), 2956 (C–H, m), 2931 (C–H, m), 2870 (C–H, m), 1725 (C=S, s), 1546 (N–H, m), 1265 (C–F, s) cm^{-1}.

HRMS (ESI, positive ion mode): *m/z* calcd for $[C_{26}H_{36}F_2N_4S_2+H]^+$ = 507.2422. Found 507.2401.

N^1,N^2-Bis(3-fluorobenzyl)ethane-1,2-diamine (29)

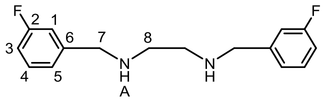

To a stirred solution of ethylene diamine (0.33 mL, 4.9 mmol, 1.0 eq.) in MeOH (100 mL) was added 3-fluorobenzaldehyde (1.06 mL, 10 mmol, 2.0 eq.) under air. The reaction mixture was stirred at r.t. for 22 h. The reaction mixture was then cooled to 0 °C and $NaBH_4$ (416 mg, 11 mmol, 2.2 eq.) was added portionwise. The reaction mixture was warmed to r.t. and stirred for 4 h. The reaction mixture was quenched with aqueous NaOH (1 M) and diluted with 3:1 $CHCl_3$/IPA. The organic layer was separated, and the aqueous layer was extracted three times with 3:1 $CHCl_3$/IPA. The organic extracts were combined, dried over $MgSO_4$, filtered, and concentrated *in vacuo*. The crude residue was purified by flash column chromatography (SiO_2, loading in CH_2Cl_2; gradient elution: 98.8:1.0:0.2 CH_2Cl_2/MeOH/[35% aqueous NH_3] to 94.0:5.0:1.0 CH_2Cl_2/MeOH/[35% aqueous NH_3]) to yield the title compound as a pale yellow liquid (915 mg, 66%).

Data consistent with literature.[3]

^{1}H NMR (400 MHz, $CDCl_3$) δ_H: 7.27 (2H, td, *J* = 7.8, 5.9 Hz, *H*4), 7.10 – 7.01 (4H, m, *H*3 and *H*5), 6.93 (2H, td, *J* = 8.5, 2.7 Hz, *H*1), 3.77 (4H, s, *H*7), 2.74 (4H, s, *H*8), 1.66 (2H, br s, N_A*H*).

^{13}C NMR (101 MHz, $CDCl_3$) δ_C: 163.1 (d, *J* = 245.6 Hz, *C*2), 143.3 (d, *J* = 6.8 Hz, *C*6), 129.9 (d, *J* = 8.2 Hz, *C*4), 123.7 (d, *J* = 2.8 Hz, *C*5), 114.9 (d, *J* = 21.2 Hz, *C*3), 113.9 (d, *J* = 21.1 Hz, *C*1), 53.5 (d, *J* = 1.9 Hz, *C*7), 48.8 (s, *C*8).

^{19}F NMR (377 MHz, $CDCl_3$) δ_F: –115.75 (ddd, *J* = 9.8, 8.7, 5.9 Hz).

1,1'-(Ethane-1,2-diyl)bis(3-butyl-1-(3-fluorobenzyl)thiourea) (23)

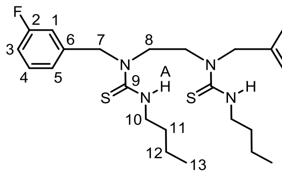

To a stirred solution of **29** (193 mg, 0.70 mmol, 1.0 eq.) in CH_2Cl_2 (22 mL) at 0 °C was added *n*-butyl isothiocyanate (0.18 mL, 1.5 mmol, 2.2 eq.) dropwise under air. The reaction mixture was warmed to r.t. and stirred for 24 h. The reaction mixture was quenched with MeOH, stirred at r.t. for 5 min, and concentrated *in vacuo*. The crude residue was purified by flash column chromatography (SiO_2, loading in CH_2Cl_2; gradient elution: 100:0 CH_2Cl_2/MeOH to 98:2 CH_2Cl_2/MeOH) to yield the title compound as a white powder (346 mg, 98%).

m.p. (CH_2Cl_2): 90 - 91 °C.

$\mathbf{R}_{\mathbf{f}}$: 0.65 (SiO_2, 90:10 CH_2Cl_2/MeOH).

^{1}H NMR peak for N_AH could not be identified.

^{1}H NMR (600 MHz, $CDCl_3$) δ_H: 7.30 (2H, q, *J* = 7.2 Hz, *H*4), 7.00 - 6.93 (4H, m, *H*3 and *H*5), 6.90 (2H, d, *J* = 9.4 Hz, *H*1), 4.81 (4H, br s, *H*7), 3.78 (4H, br s, *H*8), 3.61 (4H, br q, *J* = 6.5 Hz, *H*10), 1.59 - 1.50 (4H, m, *H*11), 1.32 - 1.21 (4H, m, *H*12), 0.89 (6H, t, *J* = 7.4 Hz, *H*13).

^{13}C NMR (151 MHz, $CDCl_3$) δ_C: 182.3 (s, *C*9), 163.3 (d, *J* = 248.1 Hz, *C*2), 138.6 (br s, *C*6), 130.9 (d, *J* = 8.3 Hz, *C*4), 122.4 (s, *C*5), 115.2 (d, *J* = 21.1 Hz, *C*3), 113.9 (d, *J* = 21.7 Hz, *C*1), 54.5 (br s, *C*7), 48.2 (br s, *C*8), 46.6 (s, *C*10), 31.1 (s, *C*11), 20.2 (s, *C*12), 13.9 (s, *C*13).

^{19}F NMR (565 MHz, $CDCl_3$) δ_F: –113.58 (br s).

IR (film): ν_{MAX} = 3242 (N–H, br), 3056 (br), 2957 (C–H, m), 2931 (C–H, m), 2872 (C–H, m), 1726 (C=S, m), 1591 (N–H, m), 1249 (C–F, s) cm^{-1}.

HRMS (ESI, positive ion mode): *m/z* calcd for $[C_{26}H_{36}F_2N_4S_2+H]^+$ = 507.2422. Found 507.2412.

***N*[1]-(4-(Trifluoromethyl)benzyl)-*N*2-(2-((4-(trifluoromethyl)benzyl)amino)ethyl)ethane-1,2-diamine (30)**

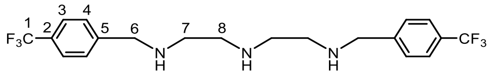

To a stirred solution of diethylenetriamine (0.43 mL, 4.0 mmol, 1.0 eq.) in MeOH (50 mL) was added 4-(trifluoromethyl)benzaldehyde (1.10 mL, 8.1 mmol, 2.0 eq.) under air. The reaction mixture was stirred at r.t. for 22 h. The reaction mixture was then cooled to 0 °C and $NaBH_4$ (333 mg, 8.8 mmol, 2.2 eq.) was added portionwise. The reaction mixture was warmed to r.t. and stirred for 4 h. The reaction mixture was quenched with aqueous NaOH (1 M) and diluted with 3:1 $CHCl_3$/IPA. The organic layer was separated, and the aqueous layer was extracted three times with 3:1 $CHCl_3$/IPA. The organic extracts were combined, dried over $MgSO_4$, filtered, and concentrated *in vacuo*. The crude residue was purified by flash column chromatography (SiO_2, loading in CH_2Cl_2; gradient elution: 98.8:1.0:0.2 CH_2Cl_2/MeOH/[35% aqueous NH_3] to 95.2:4.0:0.8 CH_2Cl_2/MeOH/[35% aqueous NH_3]) to yield the title compound as a pale yellow liquid (1.71 g, quant.).

R*f*: 0.33 (SiO_2, 88:10:2 CH_2Cl_2/MeOH/[35% aqueous NH_3]).

^{1}H NMR (400 MHz, $CDCl_3$) δ_H: 7.56 (4H, d, *J* = 8.0 Hz, *H*3), 7.43 (4H, d, *J* = 8.0 Hz, *H*4), 3.85 (4H, s, *H*6), 2.74 (8H, s, *H*7 and *H*8).

^{13}C NMR (151 MHz, $CDCl_3$) δ_C: 144.6 (s, *C*5), 129.4 (q, *J* = 32.3 Hz, *C*2), 128.4 (s, *C*4), 125.4 (q, *J* = 3.8 Hz, *C*3), 124.4 (q, *J* = 271.8 Hz, *C*1), 53.5 (s, *C*6), 49.3 (s, *C*8), 48.9 (s, *C*7).

^{19}F NMR (565 MHz, $CDCl_3$) δ_F: –62.41 (s).

IR (film): ν_{MAX} = 2932 (C–H, m), 2813 (C–H, m), 1618 (N–H, m), 1325 (s), 1162 (C–F, m) cm^{-1}.

HRMS (ESI, positive ion mode): *m/z* calcd for $[C_{20}H_{23}F_6N_3+H]^+$ = 420.1869. Found 420.1854.

3-Butyl-1,1-bis(2-(3-butyl-1-(4-(trifluoromethyl)benzyl)thioureido)ethyl)thiourea (24)

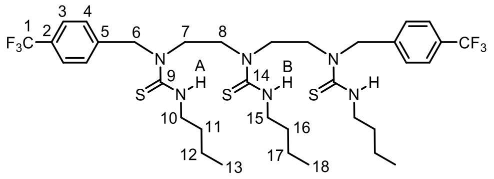

To a stirred solution of **30** (210 mg, 0.50 mmol, 1.0 eq.) in CH_2Cl_2 (10 mL) at 0 °C was added *n*-butyl isothiocyanate (0.21 mL, 1.7 mmol, 3.3 eq.) dropwise under air. The reaction mixture was warmed to r.t. and stirred for 19 h. The reaction mixture was quenched with MeOH, stirred at r.t. for 5 min, and concentrated *in vacuo*. The crude residue was purified by flash column chromatography (SiO_2, loading in CH_2Cl_2; gradient elution: 100:0 CH_2Cl_2/MeOH to 95:5 CH_2Cl_2/MeOH) to yield the title compound as a white powder (328 mg, 86%).

m.p. (CH_2Cl_2): 115 - 117 °C.

R*f*: 0.71 (SiO_2, 90:10 CH_2Cl_2/MeOH).

^{1}H NMR (500 MHz, *d*$_6$-DMSO) δ_H: 7.86 (1H, br t, *J* = 5.1 Hz, N$_B$*H*), 7.77 (2H, br s, N$_A$*H*), 7.69 (4H, d, *J* = 8.0 Hz, *H*3), 7.36 (4H, d, *J* = 8.0 Hz, *H*4), 5.02 (4H, br s, *H*6), 3.76 (8H, br s, *H*7 and *H*8), 3.48 (6H, q, *J* = 7.2 Hz, *H*10 and *H*15), 1.51 (2H, quint, *J* = 6.0 Hz, *H*16), 1.47 (4H, br s, *H*11), 1.26 (2H, sext, *J* = 7.4 Hz, *H*17), 1.17 (4H, br s, *H*12), 0.84 (3H, t, *J* = 7.4 Hz, *H*18), 0.82 (6H, brt, *J* = 7.5 Hz, *H*13).

^{13}C NMR (126 MHz, *d*$_6$-DMSO) δ_C: 181.2 (br s, *C*9), 180.7 (s, *C*14), 142.2 (br s, *C*5), 127.8 (q, *J* = 31.8 Hz, *C*2), 127.3 (br s, *C*4), 125.5 – 125.2 (m, *C*3), 124.3 (q, *J* = 271.9 Hz, *C*1), 52.8 (br s, *C*6), 47.8 (br s, *C*7 and *C*8), 45.4 (s, *C*10), 45.3 (s, *C*15), 30.7 (s, *C*16), 30.7 (s, *C*11), 19.7 (s, *C*17), 19.5 (s, *C*12), 13.7 (s, *C*13 and *C*18).

^{19}F NMR (565 MHz, $CDCl_3$) δ_F: –64.65 (br s), –64.93 (br s).

IR (film): ν_{MAX} = 3233 (N–H, br), 3053 (br), 2958 (C–H, m), 2932 (C–H, m), 2873 (C–H, m), 1724 (C=S, w), 1546 (N–H, s), 1324 (s), 1167 (C–F, s) cm^{-1}.

HRMS (ESI, positive ion mode): *m/z* calcd for $[C_{35}H_{50}F_6N_6S_3+H]^+$ = 765.3236. Found 765.3238.

***N*¹-(4-Fluorobenzyl)-*N*2-(2-((4-fluorobenzyl)amino)ethyl)ethane-1,2-diamine (31)**

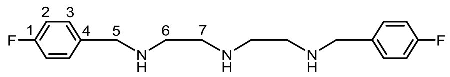

To a stirred solution of diethylenetriamine (0.43 mL, 4.0 mmol, 1.0 eq.) in MeOH (50 mL) was added 4-fluorobenzaldehyde (0.86 mL, 8.1 mmol, 2.0 eq.) under air. The reaction mixture was stirred at r.t. for 23 h. The reaction mixture was then cooled to 0 °C and $NaBH_4$ (333 mg, 8.8 mmol, 2.2 eq.) was added portionwise. The reaction mixture was warmed to r.t. and stirred for 3 h. The reaction mixture was quenched with aqueous NaOH (1 M) and diluted with 3:1 $CHCl_3$/IPA. The organic layer was separated, and the aqueous layer was extracted three times with 3:1 $CHCl_3$/IPA. The organic extracts were combined, dried over $MgSO_4$, filtered, and concentrated *in vacuo*. The crude residue was purified by flash column chromatography (SiO_2, loading in CH_2Cl_2; gradient elution: 98.8:1.0:0.2 CH_2Cl_2/MeOH/[35% aqueous NH_3] to 94.0:5.0:1.0 CH_2Cl_2/MeOH/[35% aqueous NH_3]) to yield the title compound as a colourless oil (1.09 g, 85%).

R*f*: 0.32 (SiO_2, 88:10:2 CH_2Cl_2/MeOH/[35% aqueous NH_3]).

^{1}H NMR (500 MHz, $CDCl_3$) δ_H: 7.27 (4H, dd, *J* = 8.6, 5.3 Hz, *H*3), 6.98 (4H, t, *J* = 8.6 Hz, *H*2), 3.75 (4H, s, *H*5), 2.72 (8H, s, *H*6 and *H*7).

^{13}C NMR (126 MHz, $CDCl_3$) δ_C: 162.0 (d, *J* = 244.6 Hz, *C*1), 136.3 (d, *J* = 3.1 Hz, *C*4), 129.8 (d, *J* = 8.0 Hz, *C*3), 115.3 (d, *J* = 21.2 Hz, *C*2), 53.3 (s, *C*5), 49.4 (s, *C*7), 48.9 (s, *C*6).

^{19}F NMR (377 MHz, $CDCl_3$) δ_F: –116.11 (tt, *J* = 8.8, 5.4 Hz).

IR (film): ν_{MAX} = 3440 (N–H, br), 2938 (C–H, m), 2812 (C–H, m), 1730 (m), 1509 (N–H, s), 1221 (C–F, s) cm^{-1}.

HRMS (ESI, positive ion mode): *m/z* calcd for $[C_{18}H_{23}F_2N_3+H]^+$ = 320.1933. Found 320.1925.

1,1-Bis(2-(1-(4-fluorobenzyl)-3-phenylthioureido)ethyl)-3-phenylthiourea (25)

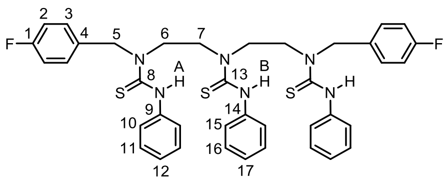

To a stirred solution of **31** (160 mg, 0.50 mmol, 1.0 eq.) in CH_2Cl_2 (10 mL) at 0 °C was added phenyl isothiocyanate (0.20 mL, 1.7 mmol, 3.3 eq.) dropwise under air. The reaction mixture was warmed to r.t. and stirred for 22 h. The reaction mixture was quenched with MeOH, stirred at r.t. for 5 min, and concentrated *in vacuo*. The crude residue was purified by flash column chromatography (SiO_2, loading in CH_2Cl_2; gradient elution: 100:0 CH_2Cl_2/MeOH to 98:2 CH_2Cl_2/MeOH) to yield the title compound as a white powder (300 mg, 83%).

m.p. (CH_2Cl_2): 134 - 135 °C.

R_f: 0.15 (SiO_2, CH_2Cl_2).

^{1}H NMR (600 MHz, d_6-DMSO) δ_H: 9.54 (1H, br s, N_BH), 9.49 (2H, br s, N_AH), 7.36 (4H, br s, *H*3), 7.33 - 7.26 (12H, m, *H*10, *H*11, *H*15, and *H*16), 7.22 (4H, t, *J* = 8.6 Hz, *H*2), 7.19 - 7.13 (3H, m, *H*12 and *H*17), 5.13 (4H, br s, *H*5), 4.03 (8H, br s, *H*6 and *H*7).

^{13}C NMR (151 MHz, d_6-DMSO) δ_C: 181.5 (s, *C*8), 180.8 (s, *C*13), 161.4 (d, *J* = 243.1 Hz, *C*1), 140.4 (s, *C*9), 140.0 (s, *C*14), 133.1 (br s, *C*4), 129.0 (br s, *C*3), 128.1 (s, *C*16), 128.0 (s, *C*11), 126.6 (br s, *C*10), 126.4 (s, *C*15), 125.3 (s, *C*12 and *C*17), 115.4 (d, *J* = 21.5 Hz, *C*2), 52.8 (br s, *C*5), 48.8 (br s, *C*6 and *C*7).

^{19}F NMR (565 MHz, $CDCl_3$) δ_F: –114.05 (br s), –115.77 (br s).

IR (film): ν_{MAX} = 3209 (N–H, br), 3029 (br), 2938 (C–H, m), 1733 (C=S, w), 1542 (N–H, m), 1508 (s), 1355 (s), 1209 (C–F, s) cm^{-1}.

HRMS (ESI, positive ion mode): *m/z* calcd for $[C_{39}H_{38}F_2N_6S_3+H]^+$ = 725.2361. Found 725.2338.

1,1-Bis(2-(1-(2-aminoethyl)-3-butylureido)ethyl)-3-butylurea (33)

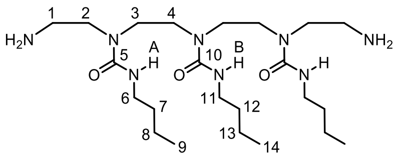

Step 1 *di-trifluoroacetylation and tri-urea formation*: To a stirred solution of tetraethylenepentamine (1.90 mL, 10 mmol, 1.0 eq.) in CH_2Cl_2 (150 mL) at 0 °C was added ethyl trifluoroacetate (4.20 mL, 35 mmol, 3.5 eq.) dropwise under air. The reaction mixture was warmed to r.t. and stirred for 23 h. The reaction mixture was then cooled to 0 °C and *n*-butyl isocyanate (3.70 mL, 33 mmol, 3.3 eq.) was added dropwise. The reaction mixture was stirred at 0 °C for 5 min and then stirred at r.t. for 7 h. The reaction mixture was quenched with MeOH, stirred at r.t. for 5 min, and concentrated *in vacuo*. The crude residue was purified by flash column chromatography (SiO_2, loading in CH_2Cl_2; gradient elution: 2:98 MeOH/CH_2Cl_2 to 8:92 MeOH/CH_2Cl_2) to yield the intermediate di-protected tri-urea (**32**) as a white powder (3.79 g, 5.6 mmol).

Step 2 *di-trifluoroacetamide deprotection*: To a stirred solution of the material from **step 1** (3.79 g, 5.6 mmol, 1.0 eq.) in THF (29 mL) and EtOH (58 mL) was added NaOH (1.76 g, 44 mmol, 7.9 eq.) in H_2O (29 mL) under air. The reaction mixture was stirred at r.t. for 5 h and then concentrated *in vacuo*. The resulting mixture was diluted with 3:1 $CHCl_3$/IPA and H_2O, and the organic layer was separated. The aqueous layer was extracted three times with 3:1 $CHCl_3$/IPA. The organic extracts were combined, dried over $MgSO_4$, filtered, and concentrated *in vacuo* to yield the title compound as a colourless oil (2.19 g, 45% over two steps).

R*f*: 0.07 (SiO_2, 90:10 CH_2Cl_2/MeOH).

^{1}H NMR (600 MHz, $CDCl_3$) δ_H: 6.67 (1H, t, *J* = 5.2 Hz, N_BH), 6.64 (2H, t, *J* = 5.5 Hz, N_AH), 3.32 - 3.26 (8H, m, *H*2 and *H*3), 3.23 (4H, t, *J* = 5.7 Hz, *H*4), 3.13 (6H, q, *J* = 5.9 Hz, *H*6 and *H*11), 2.80 (4H, t, *J* = 5.2 Hz, *H*1), 1.44 (6H, quint, *J* = 5.5 Hz, *H7* and *H*12), 1.31 (6H, sext, *J* = 5.5 Hz, *H*8 and *H*13), 0.88 (3H, t, *J* = 7.3 Hz, *H*14), 0.86 (6H, t, *J* = 7.5 Hz, *H*9).

^{13}C NMR (151 MHz, $CDCl_3$) δ_C: 160.1 (s, *C*10), 158.8 (s, *C*5), 51.6 (s, *C*2), 47.0 (s, *C*3 and *C*4), 41.4 (s, *C*1), 40.6 (s, *C*6 and *C*11), 32.3 (s, *C*7 and *C*12), 20.3 (s, *C*8 and *C*13), 13.9 (s, *C*9 and *C*14).

IR (film): ν_{MAX} = 3299 (N–H, br), 3076 (br), 2940 (C–H, m), 1708 (m, C=O), 1538 (N–H, m), 1513 (s) cm^{-1}.

HRMS (ESI, positive ion mode): *m/z* calcd for $[C_{23}H_{50}N_8O_3+H]^+$ = 487.4079. Found 487.4083.

3-Butyl-1,1-bis(2-(3-butyl-1-(2-((4-fluorobenzyl)amino)ethyl)ureido)ethyl)urea (34)

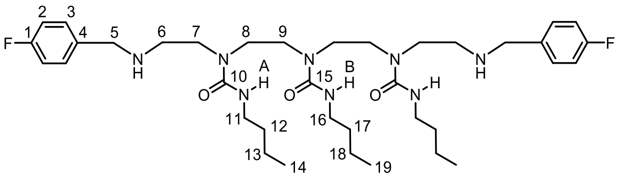

To a stirred solution of **33** (386 mg, 0.79 mmol, 1.0 eq.) in MeOH (15 mL) was added 4-fluorobenzaldehyde (0.17 mL, 1.6 mmol, 2.0 eq.) under air. The reaction mixture was stirred at r.t. for 23 h. The reaction mixture was then cooled to 0 °C and $NaBH_4$ (68 mg, 1.8 mmol, 2.2 eq.) was added portionwise. The reaction mixture was warmed to r.t. and stirred for 3 h. The reaction mixture was quenched with aqueous NaOH (1 M) and diluted with 3:1 $CHCl_3$/IPA. The organic layer was separated, and the aqueous layer was extracted three times with 3:1 $CHCl_3$/IPA. The organic extracts were combined, dried over $MgSO_4$, filtered, and concentrated *in vacuo*. The crude residue was purified by flash column chromatography (SiO_2, loading in CH_2Cl_2; gradient elution: 98:2 CH_2Cl_2/MeOH to 85:15 CH_2Cl_2/MeOH) to yield the title compound as a colourless oil (141 mg, 25%).

R_f: 0.23 (SiO_2, 90:10 CH_2Cl_2/MeOH).

^{1}H NMR (400 MHz, $CDCl_3$) δ_H: 7.29 (4H, dd, *J* = 8.4, 5.3 Hz, *H*3), 6.99 (4H, t, *J* = 8.4 Hz, *H*2), 6.68 (3H, brt, *J* = 5.7 Hz, N_AH and N_BH), 3.79 (4H, s, *H*5), 3.36 (4H, t, *J* = 5.7 Hz, *H*7), 3.25 (8H, t, *J* = 4.8 Hz, *H*8 and *H*9), 3.11 (6H, td, *J* = 7.2, 5.5 Hz, *H*11 and *H*16), 2.81 (4H, t, *J* = 5.3 Hz, *H*6), 1.48 (2H, quint, *J* = 7.2 Hz, *H*17), 1.40 (4H, quint, *J* = 7.0 Hz, *H*12), 1.28 (6H, sext, *J* = 7.2 Hz, *H*13 and *H*18), 0.90 (3H, t, *J* = 7.4 Hz, *H*19), 0.86 (6H, t, *J* = 7.4 Hz, *H*14).

^{13}C NMR (151 MHz, $CDCl_3$) δ_C: 162.8 (d, *J* = 245.2 Hz, *C*1), 159.8 (s, *C*15), 158.9 (s, *C*10), 133.6 (s, *C*4), 130.3 (d, *J* = 4.7 Hz, *C*3), 115.5 (d, *J* = 21.4 Hz, *C*2), 52.9 (s, *C*5), 48.5 (s, *C*6 and *C*7), 47.2 (s, *C*8 or *C*9), 46.9 (s, *C*8 or *C*9), 40.8 (s, *C*16), 40.7 (s, *C*11), 32.3 (s, *C*12), 32.2 (s, *C*17), 20.3 (s, *C*13), 20.2 (s, *C*18), 13.9 (s, *C*19), 13.9 (s, *C*14).

^{19}F NMR (377 MHz, $CDCl_3$) δ_F: –117.68 (br s), –117.87 (br s).

IR (film): ν_{MAX} = 3297 (N–H, br), 2962 (C–H, m), 2931 (C–H, m), 2874 (C–H, w), 1726 (m, C=O), 1629 (s), 1542 (N–H, m), 1513 (s), 1225 (C–F, s) cm^{-1}.

HRMS (ESI, positive ion mode): *m/z* calcd for $[C_{37}H_{60}F_2N_8O_3+H]^+$ = 703.4829. Found 703.4810.

3-Butyl-1,1-bis(2-(3-butyl-1-(2-(3-butyl-1-(4-fluorobenzyl)thioureido)ethyl)ureido)ethyl)urea (26)

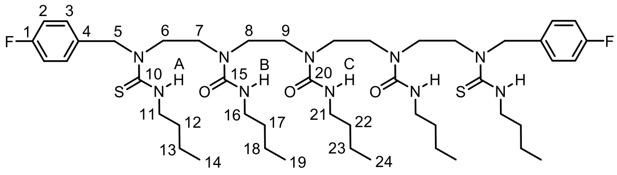

To a stirred solution of **34** (226 mg, 0.32 mmol, 1.0 eq.) in CH_2Cl_2 (12 mL) at 0 °C was added *n*-butyl isothiocyanate (0.10 mL, 0.83 mmol, 2.6 eq.) dropwise under air. The reaction mixture was warmed to r.t. and stirred for 4 h. The reaction mixture was quenched with MeOH, stirred at r.t. for 5 min, and concentrated *in vacuo*. The crude residue was purified by flash column chromatography (SiO_2, loading in CH_2Cl_2; gradient elution: 100:0 CH_2Cl_2/MeOH to 95:5 CH_2Cl_2/MeOH) to yield the title compound as a colourless oil (135 mg, 45%).

R*f*: 0.40 (SiO_2, 90:10 CH_2Cl_2/MeOH).

^{1}H NMR peak for N_AH could not be identified.

^{1}H NMR (600 MHz, $CDCl_3$) δ_H: 7.18 (4H, dd, *J* = 8.4, 5.3 Hz, *H*3), 7.04 (4H, t, *J* = 8.4 Hz, *H*2), 6.54 (3H, br s, N_BH and N_CH), 4.78 (4H, br s, *H*5), 3.80 (4H, br s, *H*6), 3.52 (4H, br q, *J* = 6.5 Hz, *H*11), 3.41 (4H, br s, *H*7), 3.23 (8H, br s, *H*8 and *H*9), 3.19 (6H, q, *J* = 6.5 Hz, *H*16 and *H*21), 1.53 (6H, quint, *J* = 7.4 Hz, *H*17 and *H*22), 1.45 (4H, br s, *H*12), 1.35 (6H, sext, *J* = 7.2 Hz, *H*18 and *H*23), 1.18 (4H, br s, *H*13), 0.92 (6H, t, *J* = 7.4 Hz, *H*19), 0.90 (3H, t, *J* = 7.3 Hz, *H*24), 0.85 (6H, brt, *J* = 7.4 Hz, *H*14).

^{13}C NMR (151 MHz, $CDCl_3$) δ_C: 182.1 (s, *C*10), 162.6 (d, *J* = 246.8 Hz, *C*1), 158.9 (s, *C*20), 158.8 (s, *C*15), 131.0 (br s, *C*4), 128.2 (s, *C*3), 116.3 (s, *C*2), 53.9 (br s, *C*5), 47.8 (s, *C*6), 47.5 (s, *C*7), 46.4 (s, *C*8 and *C*9), 46.2 (s, *C*11), 40.8 (s, *C*16 and *C*21), 32.2 (s, *C*17 and *C*22), 31.0 (s, *C*12), 20.4 (s, *C*18), 20.3 (s, *C*23), 20.1 (s, *C*13), 14.0 (s, *C*24), 14.0 (s, *C*19), 13.8 (s, *C*14).

^{19}F NMR (565 MHz, CD_2Cl_2) δ_F: –116.55 (br s), –118.39 (br s).

IR (film): ν_{MAX} = 3276 (N–H, br), 2958 (C–H, m), 2938 (C–H, m), 2870 (C–H, m), 1726 (C=O, s), 1630 (s), 1546 (N–H, s), 1265 (C–F, s) cm^{-1}.

HRMS (ESI, positive ion mode): *m/z* calcd for $[C_{47}H_{78}F_2N_{10}O_3S_2+H]^+$ = 933.5741. Found 933.5734.

1-(3,6-Bis(butylcarbamoyl)-9-(4-fluorobenzyl)-10-oxo-3,6,9,11-tetraazapentadecyl)-3-butyl-1-(2-(3-butyl-1-(4-fluorobenzyl)ureido)ethyl)urea (27)

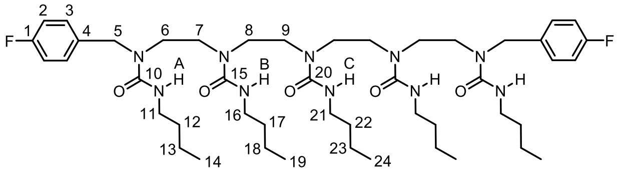

To a stirred solution of **34** (107 mg, 0.15 mmol, 1.0 eq.) in CH_2Cl_2 (5.0 mL) at 0 °C was added *n*-butyl isocyanate (37 µL, 0.33 mmol, 2.2 eq.) dropwise under air. The reaction mixture was warmed to r.t. and stirred for 19 h. The reaction mixture was quenched with MeOH, stirred at r.t. for 5 min, and concentrated *in vacuo*. The crude residue was purified by flash column chromatography (SiO_2, loading in CH_2Cl_2; gradient elution: 99:1 CH_2Cl_2/MeOH to 95:5 CH_2Cl_2/MeOH) to yield the title compound as a colourless oil (65 mg, 48%).

R*f*: 0.38 (SiO_2, 90:10 CH_2Cl_2/MeOH).

^{1}H NMR (400 MHz, $CDCl_3$) δ_H: 7.18 (4H, dd, *J* = 8.4, 5.3 Hz, *H*3), 7.02 (4H, t, *J* = 8.4 Hz, *H*2), 6.64 (5H, br s, N_AH, N_BH and N_CH), 4.40 (4H, s, *H*5), 3.35 (4H, t, *J* = 7.2 Hz, *H*6), 3.25 (4H, br s, *H*7), 3.22 – 3.13 (18H, m, *H*8, *H*9, *H*11, *H*16 and *H*21), 1.58 – 1.45 (6H, m, *H*17 and *H*22), 1.44 – 1.29 (10H, m, *H*12, *H*18, and *H*23), 1.22 (4H, sext, *J* = 7.3 Hz, *H*13), 0.91 (6H, t, *J* = 7.3 Hz, *H*19), 0.89 (3H, t, *J* = 7.3 Hz, *H*24), 0.86 (6H, t, *J* = 7.3 Hz, *H*14).

^{13}C NMR (151 MHz, $CDCl_3$) δ_C: 162.3 (d, *J* = 246.3 Hz, *C*1), 158.9 (s, *C*20), 158.8 (s, *C*15), 158.7 (s, *C*10), 133.4 (s, *C*4), 128.4 (s, *C*3), 116.0 (d, *J* = 22.6 Hz, *C*2), 51.6 (br s, *C*5), 47.7 (s, *C*8 or *C*9), 47.6 (s, *C*8 or *C*9), 47.4 (s, *C*6 and *C*7), 40.8 (s, *C*16 and *C*21), 40.8 (s, *C*11), 32.2 (s, *C*12 or *C*17), 32.2 (s, *C*22, and *C*12 or *C*17), 20.3 (s, *C*18 and *C*23), 20.1 (s, *C*13), 14.0 (s, *C*19 and *C*24), 13.9 (s, *C*14).

^{19}F NMR (565 MHz, CD_2Cl_2) δ_F: –117.85 (br s).

IR (film): ν_{MAX} = 3304 (N–H, br), 2958 (C–H, m), 2931 (C–H, m), 2870 (C–H, w), 1726 (m, C=O), 1628 (s), 1541 (N–H, m), 1269 (C–F, s) cm^{-1}.

HRMS (ESI, positive ion mode): *m/z* calcd for $[C_{47}H_{78}F_2N_{10}O_5+H]^+$ = 901.6197. Found 901.6179.

S3 Membrane Preparation

S3.1 General procedure for vesicle preparation

To an Eppendorf tube the following solutions were added: a solution of the lipid 1,2-dioleoyl-*sn*-glycero-3-phosphocholine (DOPC) or 1,2-dioleoyl-*sn*-glycero-3-phosphate sodium salt (DOPA) in $CDCl_3$ to obtain a final 10 mM or 100 mM concentration of lipids, respectively. The solvent was removed by flushing with nitrogen and the lipid film was dried under high vacuum for 16 hours. The lipid film was then rehydrated with MOPS (20 mM, pH 7.4), NaCl (100 mM), KF (0.5 mM for samples of DOPC vesicles or 0.05 mM for samples of DOPA vesicles). The chemical shift of the $F^-_{(aq)}$ peak was set to −125.3 ppm to reference the ^{19}F-NMR spectra in D_2O. In addition, the buffer was filtered through a Whatman® Anotop® 10 syringe filter with 0.02 μm pores. The suspension was then sonicated for one minute, followed by five cycles of freeze-thaw using liquid nitrogen and a water bath at 40 °C. The size reduction of the formed vesicles was carried out with a step-down extrusion using an Avestin Lipofast apparatus.[4] The suspension of DOPC vesicles was extruded 20 times through a polycarbonate filter with 800 nm, 100 nm, 50 nm and 19 times through a filter with 30 nm pores. The foldamers **26**, **27** or **5** (0.5 mM, dissolved in MeOD) was then added to the vesicles sample and analysed with ^{1}H and ^{19}F NMR spectroscopy.

S3.2 General procedure for micelle preparation

Sodium dodecyl sulfate (SDS, to achieve a final 200 mM concentration of detergent) in an Eppendorf tube was suspended in MOPS (20 mM, pH 7.4), NaCl (100 mM), KF (0.05 mM) and then subjected to five cycles of vortexing and heating in a water bath at 70 °C to obtain a transparent suspension. The foldamer (1 mM, dissolved in 9:1 MeOD/d_6-DMSO) was then added to the micelles sample and analysed with ^{1}H and ^{19}F NMR spectroscopy.

S3.3 General procedure for bicelle preparation

To an Eppendorf tube the following solutions were added: a solution of the lipid 1,2-dilauroyl-*sn*-glycero-3-phosphocholine (DLPC) in $CDCl_3$ and a solution of the detergent 1,2-dihexanoyl-*sn*-glycero-3-phosphocholine (DHPC) in $CDCl_3$ to obtain a final 300 mM concentration of lipid and detergent with a q of 0.5). The solvent was removed by flushing with nitrogen and the lipid film was dried under high vacuum for 16 hours. The lipid film was then rehydrated with MOPS (20 mM, pH 7.4), NaCl (100 mM), KF (0.05 mM) and subjected to five cycles of vortexing and heating in a water bath at 50 °C to obtain a transparent suspension. The foldamer (1 mM, dissolved in in 9:1 MeOD/d_6-DMSO) was then added to the bicelles sample and analysed with ^{1}H and ^{19}F NMR spectroscopy.

S3.4 Dynamic Light Scattering

The samples of vesicles, micelles and bicelles were analysed by Dynamic Light Scattering (DLS) before and after the addition of the foldamers (Tables S1-S3), and at temperatures between 5 °C and 45 °C (Table S4).

Lipid	Foldamer	Diameter of vesicles	Diameter of vesicles with the foldamer
DOPC (10 mM)	**26** (0.5 mM)	73.6 ± 1.1 nm	155.8 ± 4.4 nm
DOPC (10 mM)	**27** (0.5 mM)	73.3 ± 0.3 nm	138.3 ± 2.2 nm

Table S1. Average hydrodynamic diameters of the vesicles samples before and after the addition of the foldamers at 25 °C.

Detergent	Foldamer	Diameter of micelles	Detergent	Foldamer	Diameter of micelles
SDS (200 mM)	-	5.9 ± 0.1 nm	SDS (200 mM)	**5** (1 mM)	5.9 ± 0.6 nm
SDS (200 mM)	**1** (1 mM)	4.8 ± 0.2 nm	SDS (200 mM)	**24** (1 mM)	4.7 ± 0.2 nm
SDS (200 mM)	**2** (1 mM)	5.3 ± 0.3 nm	SDS (200 mM)	**25** (1 mM)	7.5 ± 0.2 nm
SDS (200 mM)	**3** (1 mM)	5.9 ± 0.3 nm	SDS (200 mM)	**26** (1 mM)	5.2 ± 0.3 nm
SDS (200 mM)	**4** (1 mM)	5.0 ± 0.5 nm	SDS (200 mM)	**27** (1 mM)	6.6 ± 0.1 nm

Table S2. Average hydrodynamic diameters of the micelles samples before and after the addition of the foldamers at 25 °C.

Lipid:Detergent	Foldamer	Diameter of bicelles	Detergent	Foldamer	Diameter of bicelles
DLPC:DHPC (300 mM)	-	6.9 ± 0.3 nm	DLPC:DHPC (300 mM)	**5** (1 mM)	7.9 ± 0.1 nm
DLPC:DHPC (300 mM)	**1** (1 mM)	7.9 ± 0.1 nm	DLPC:DHPC (300 mM)	**24** (1 mM)	8.5 ± 0.3 nm
DLPC:DHPC (300 mM)	**2** (1 mM)	7.6 ± 0.2 nm	DLPC:DHPC (300 mM)	**25** (1 mM)	7.4 ± 0.1 nm
DLPC:DHPC (300 mM)	**3** (1 mM)	7.5 ± 0.2 nm	DLPC:DHPC (300 mM)	**26** (1 mM)	7.9 ± 0.3 nm
DLPC:DHPC (300 mM)	**4** (1 mM)	7.4 ± 0.3 nm	DLPC:DHPC (300 mM)	**27** (1 mM)	7.4 ± 0.1 nm

Table S3. Average hydrodynamic diameters of the bicelles samples (q = 0.5) before and after the addition of the foldamers at 25 °C.

T / °C	Micelles	Bicelles
45 °C	4.4 ± 0.1 nm	4.4 ± 0.3 nm
35 °C	5.2 ± 0.3 nm	5.0 ± 0.1 nm
25 °C	5.9 ± 0.1 nm	6.2 ± 0.2 nm
15 °C	8.9 ± 0.2 nm	7.4 ± 0.1 nm
5 °C	11.1 ± 0.6 nm	10.2 ± 0.7 nm

Table S4. Average hydrodynamic diameters of the micelles (SDS, 200 mM), bicelles (DLPC:DHPC, 300 mM, q = 0.5) samples at 5 °C, 15 °C, 25 °C, 35 °C and 45 °C.

DLS of the bicelle suspensions showed that their size became about 1 nm bigger upon addition of the compounds. Temperature variations between 278 K and 318 K led to an increase in diameter of up to 10 nm at low temperature and a decrease to 4 nm at high temperature (see Section S3.4 in the Supporting Information). Such temperature-dependent size changes are typical for DHPC:long-chain phosphatidylcholine bicelles and reflect shifts in lipid mixing that modulate bicelle morphology and local membrane environments.[5]

S4 NMR Studies

S4.1 ^{19}F Referencing

^{19}F NMR spectra of samples in organic solvents were referenced through use of a sealed glass capillary containing C_6F_6 (δ_F = – 164.90 ppm, 8.5 mM) in CD_2Cl_2 placed inside the NMR tube. Figure S2 reports an example of a referenced spectrum.

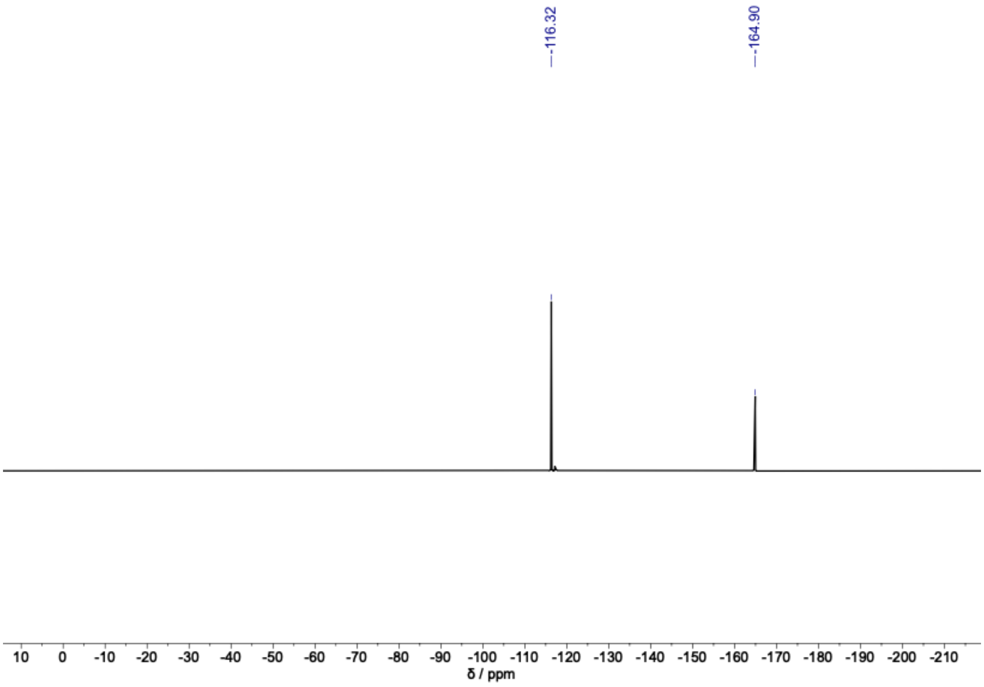

Figure S2. 565 MHz ^{19}F NMR spectrum of **1** (8 mM) in $CDCl_3$ at 298 K.

In the case of aqueous samples, ^{19}F NMR chemical shifts were referenced using the signal of $F^-_{(aq)}$ (δ_F = – 125.30 ppm, 0.05 mM). Figure S3 reports an example of a referenced spectrum.

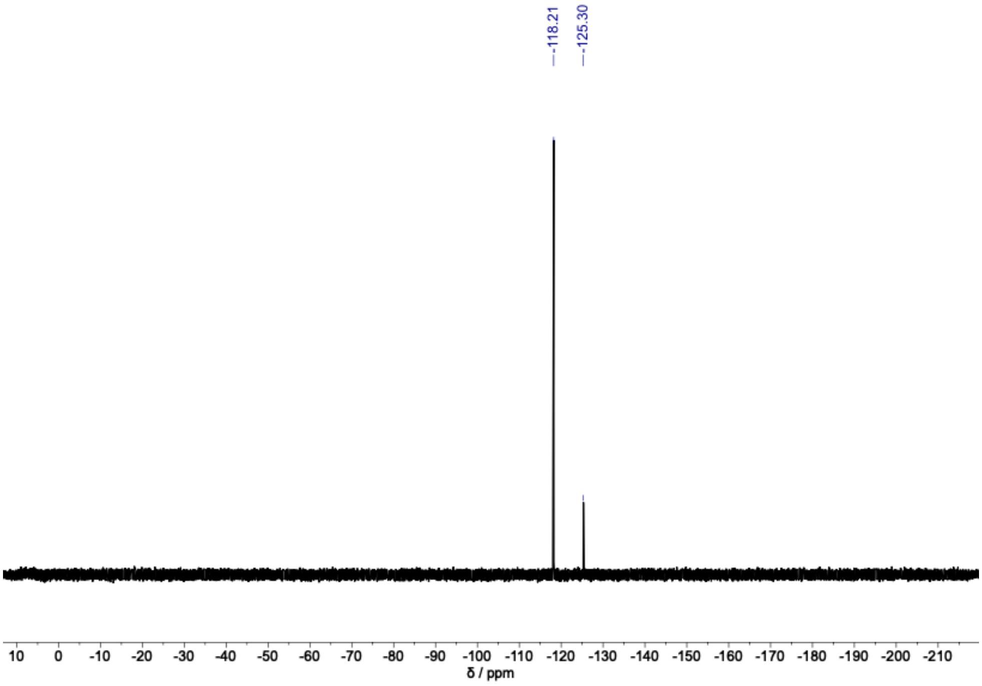

Figure S3. 565 MHz ^{19}F VT-NMR spectra **1** (1 mM) embedded in micelles (SDS, 200 mM) in MOPS (20 mM, pH 7.4), NaCl (100 mM), KF (0.05 mM) at 298 K.

S4.2 VT NMR in $CDCl_3$

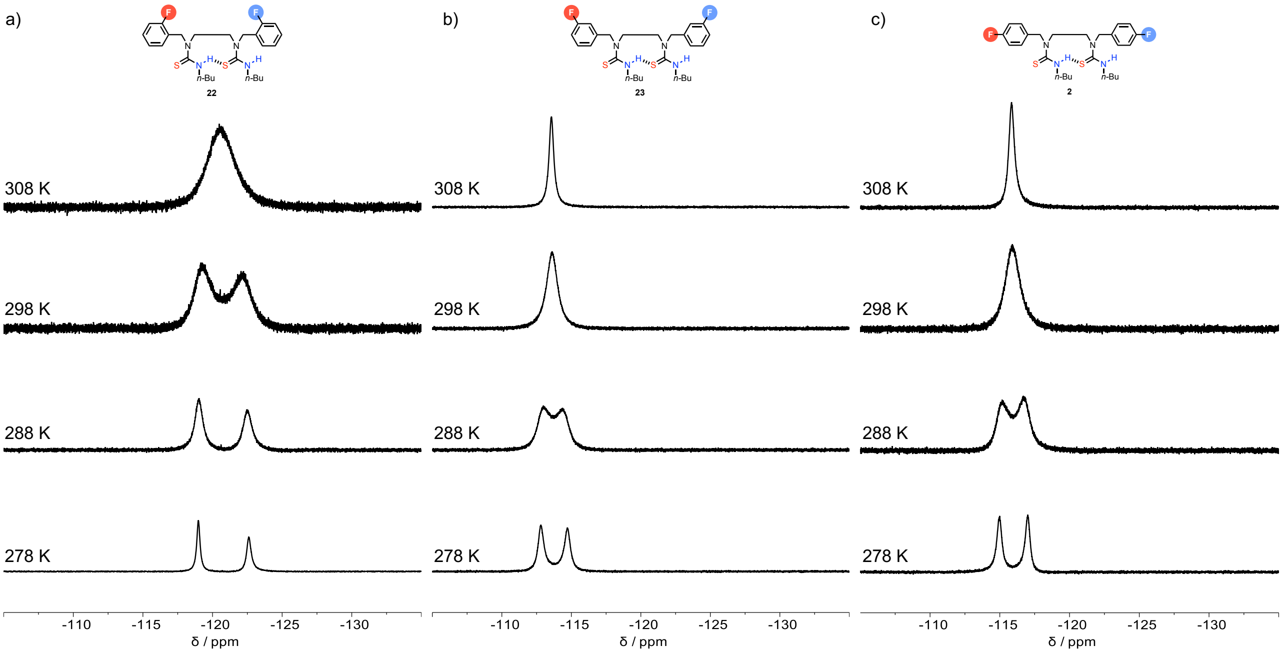

Figure S4. 565 MHz ^{19}F VT-NMR spectra of foldamers **22** (8 mM), **23** (8 mM), **2** (8 mM) in $CDCl_3$.

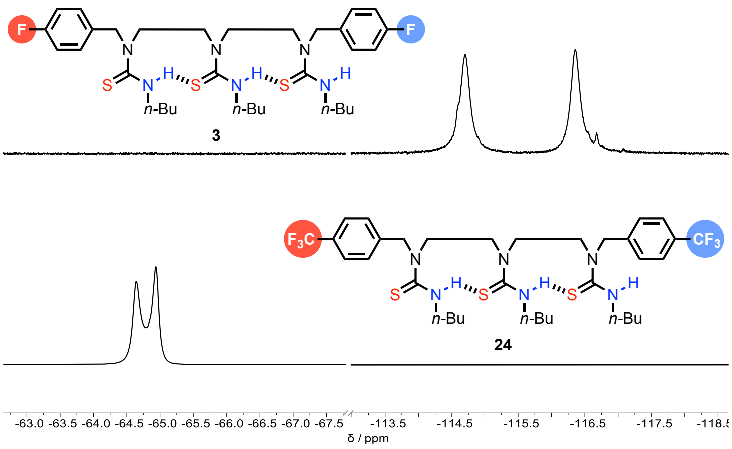

Figure S5. 565 MHz ^{19}F NMR spectra of foldamers **3** (8 mM) and **24** (8 mM) at 298 K in $CDCl_3$.

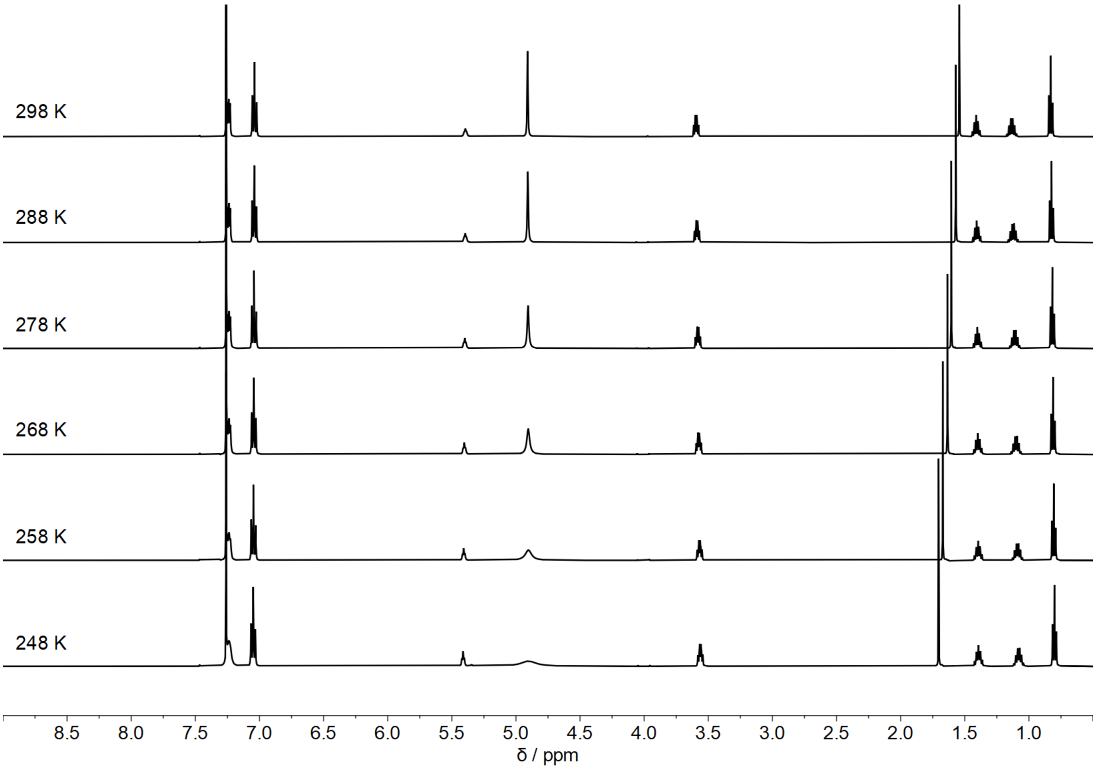

Figure S6. 500 MHz ^{1}H VT NMR of **1** (8 mM) in $CDCl_3$.

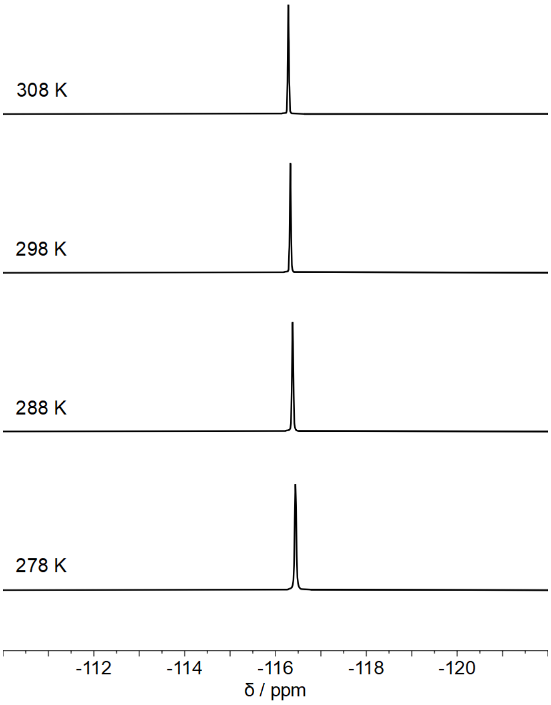

Figure S7. 565 MHz ^{19}F VT NMR of **1** (8 mM) in $CDCl_3$.

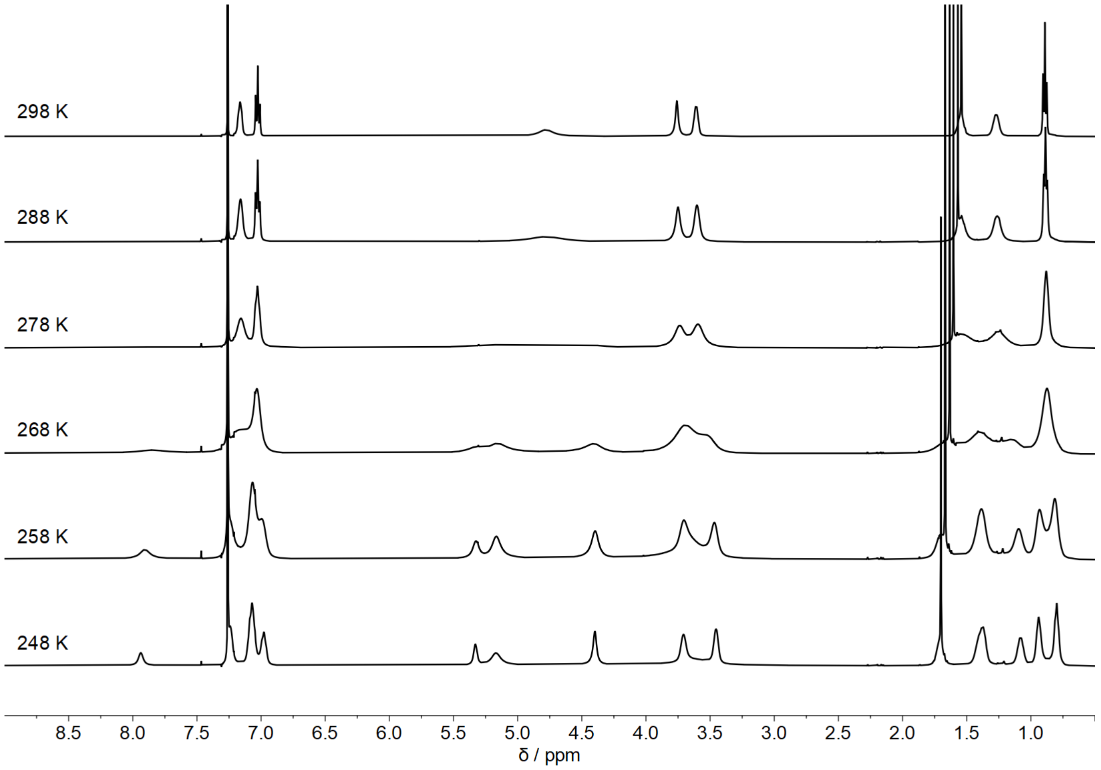

Figure S8. 500 MHz ¹H VT NMR of **2** (8 mM) in $CDCl_3$.

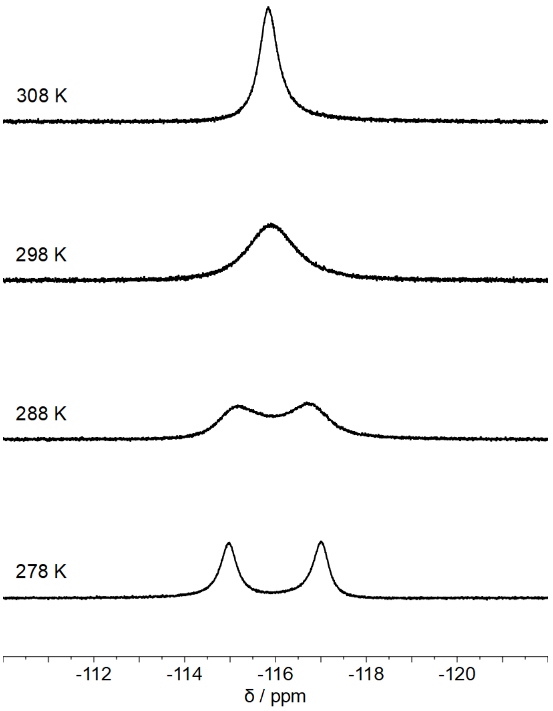

Figure S9. 565 MHz ¹⁹F VT NMR of **2** (8 mM) in $CDCl_3$.

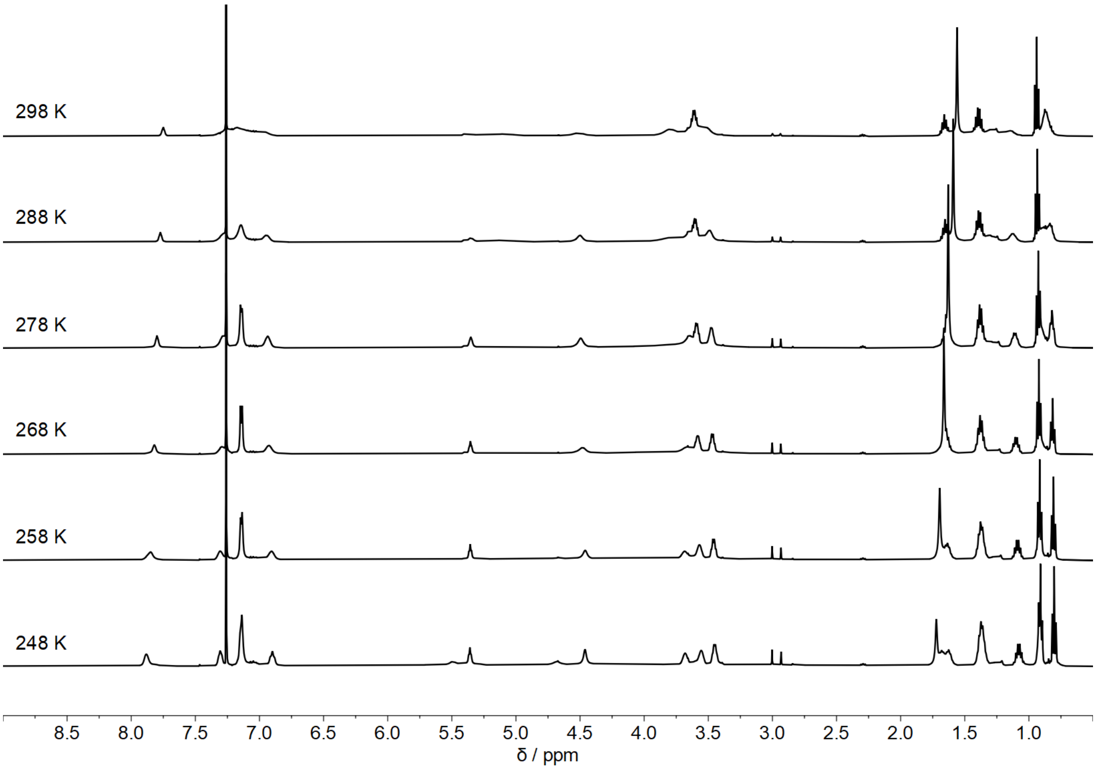

Figure S10. 500 MHz ^{1}H VT NMR of **3** (8 mM) in $CDCl_3$.

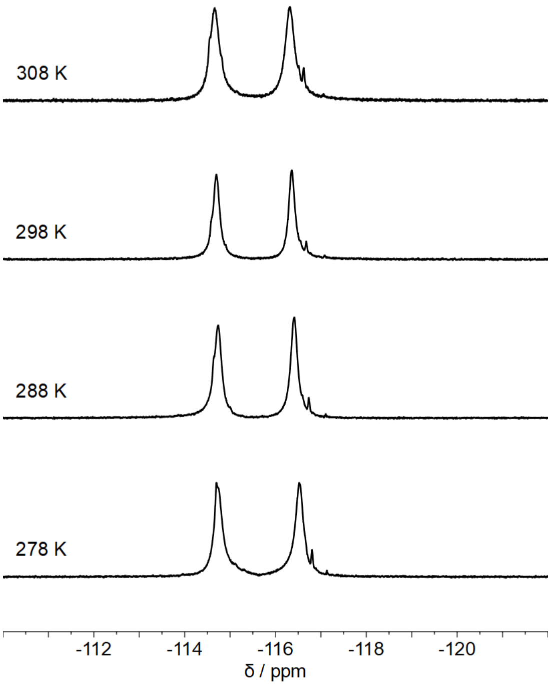

Figure S11. 565 MHz ^{19}F VT NMR of **3** (8 mM) in $CDCl_3$.

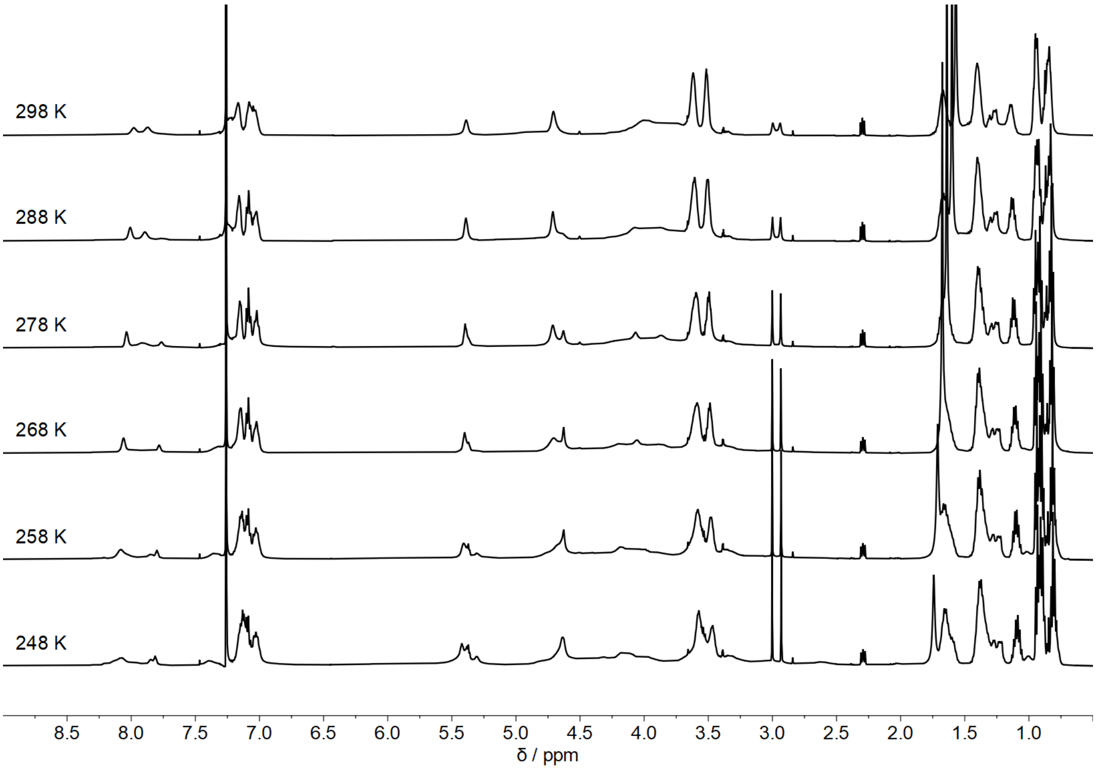

Figure S12. 500 MHz ^{1}H VT NMR of **4** (8 mM) in $CDCl_3$.

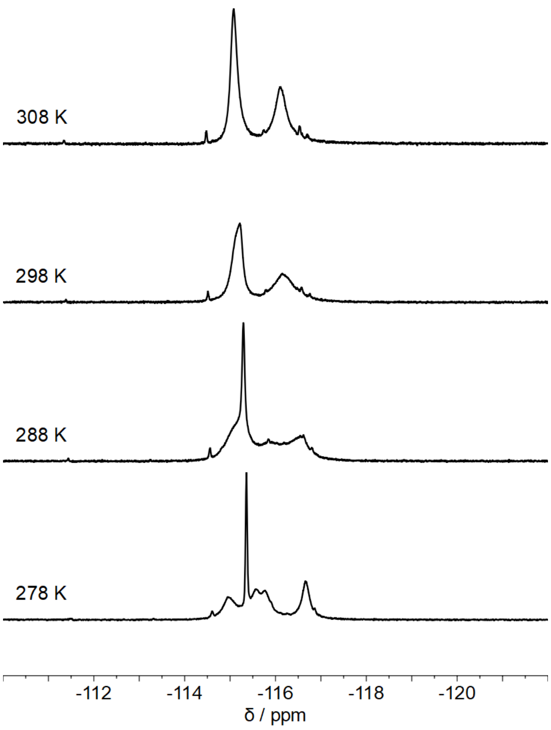

Figure S13. 565 MHz ^{19}F VT NMR of **4** (8 mM) in $CDCl_3$.

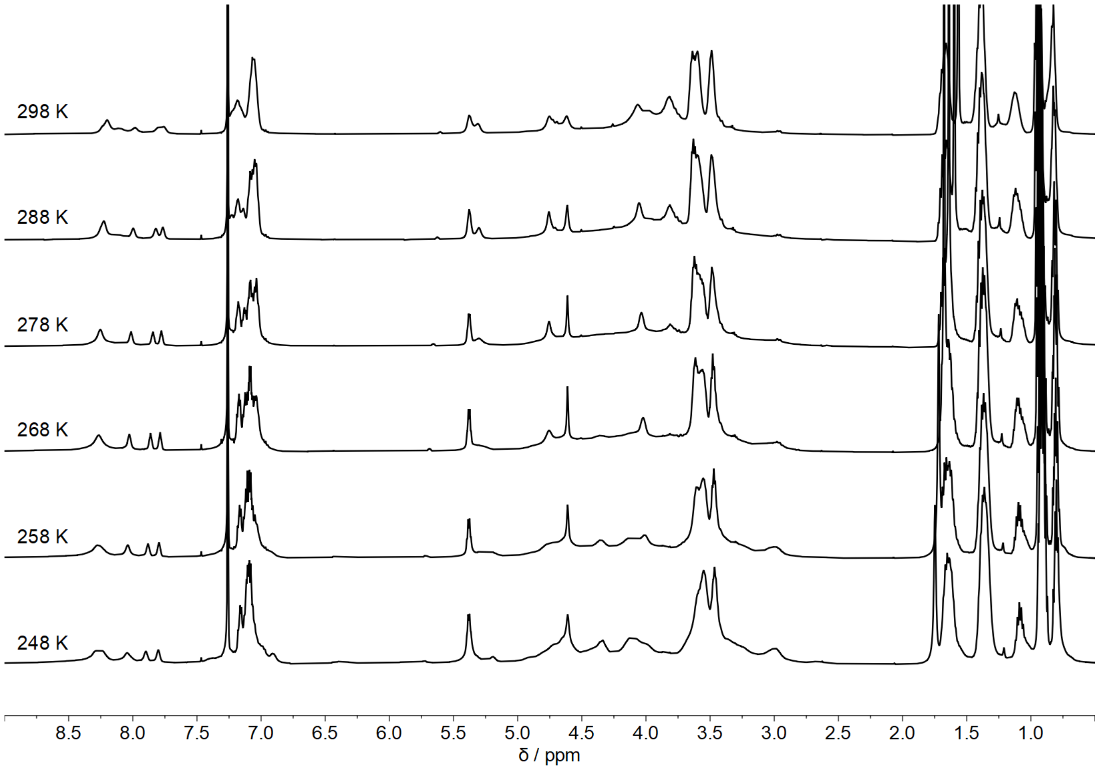

Figure S14. 500 MHz ^{1}H VT NMR of **5** (8 mM) in $CDCl_3$.

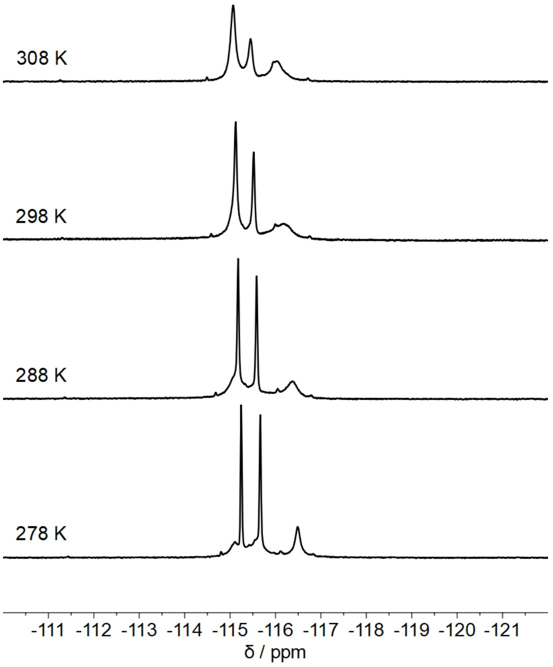

Figure S15. 565 MHz ^{19}F VT NMR of **5** (8 mM) in $CDCl_3$.

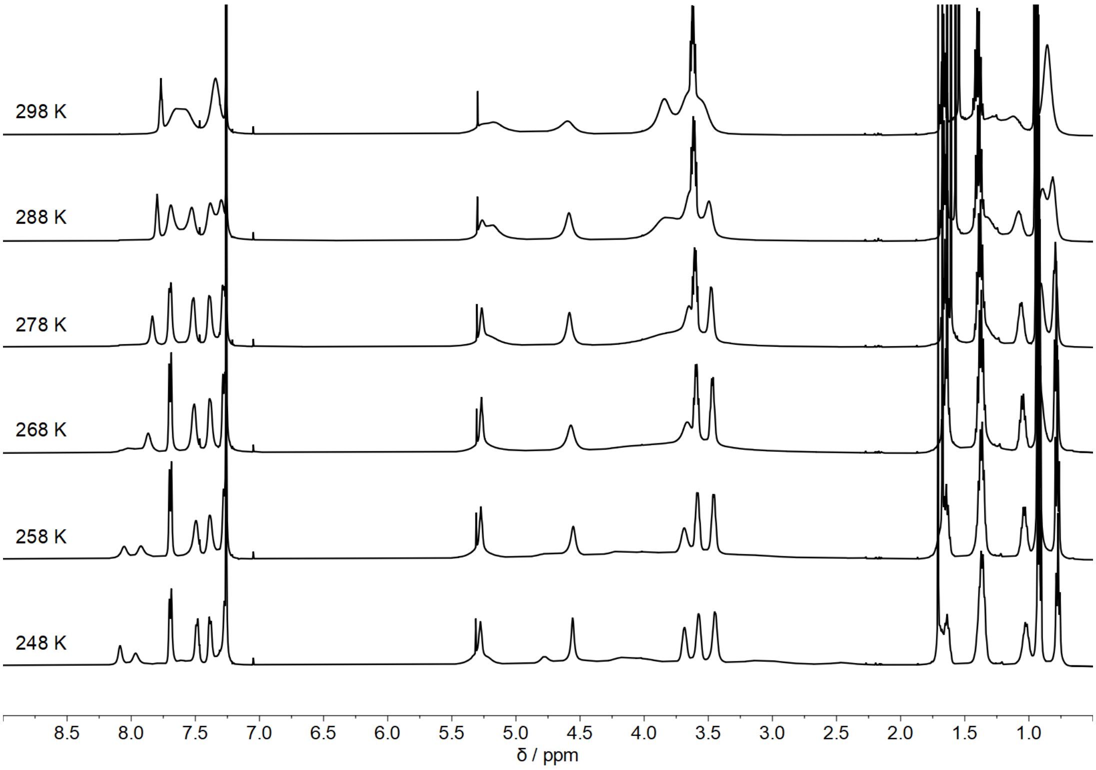

Figure S16. 500 MHz ^{1}H VT NMR of **24** (8 mM) in $CDCl_3$.

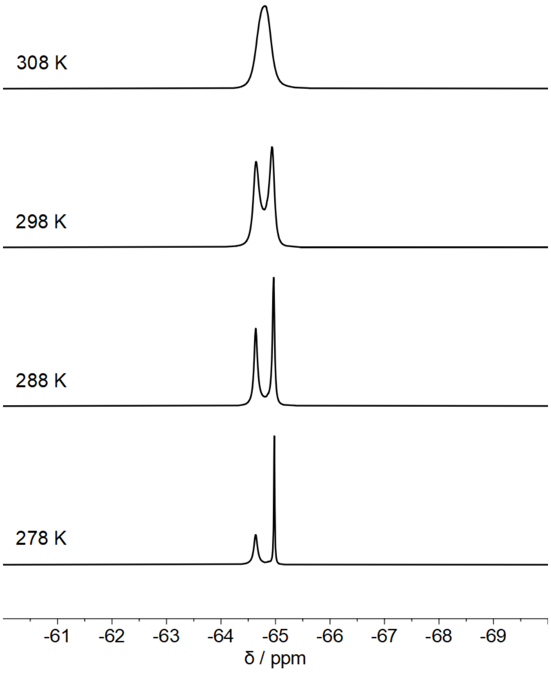

Figure S17. 565 MHz ^{19}F VT NMR of **24** (8 mM) in $CDCl_3$.

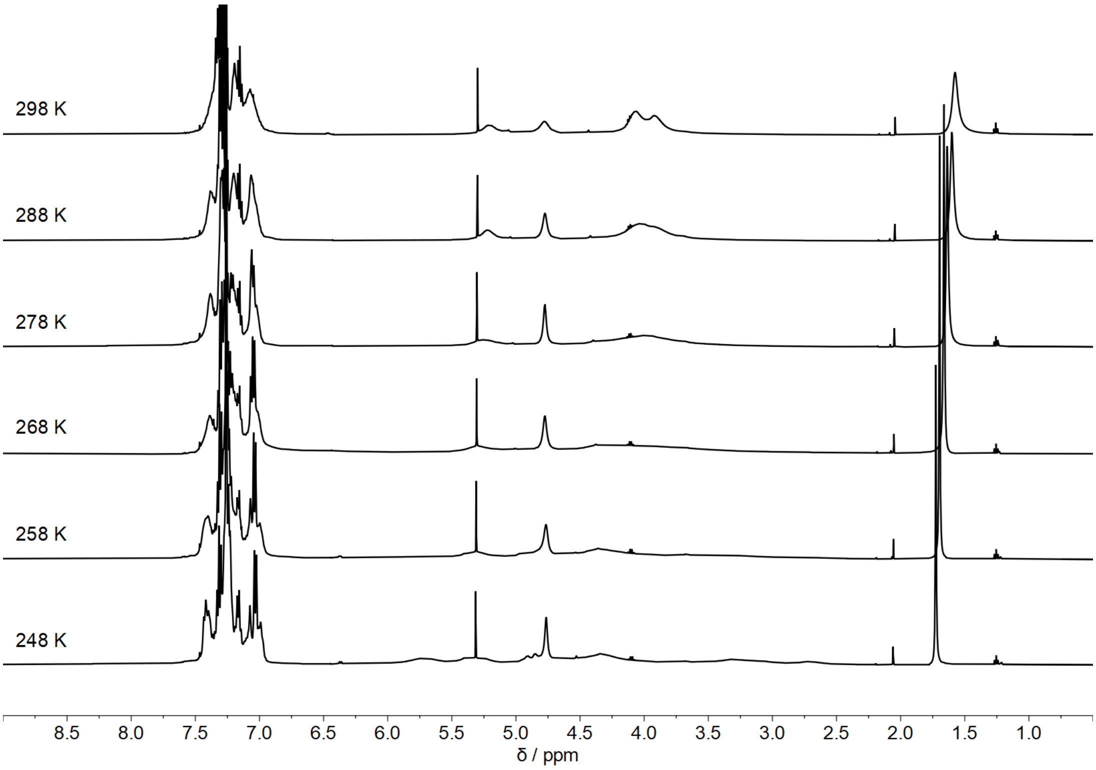

Figure S18. 500 MHz ^{1}H VT NMR of **25** (8 mM) in $CDCl_3$.

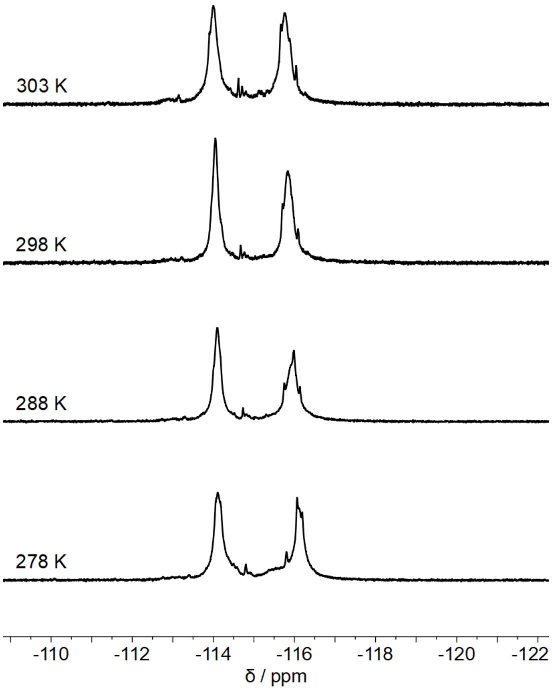

Figure S19. 565 MHz ^{19}F VT NMR of **25** (8 mM) in $CDCl_3$.

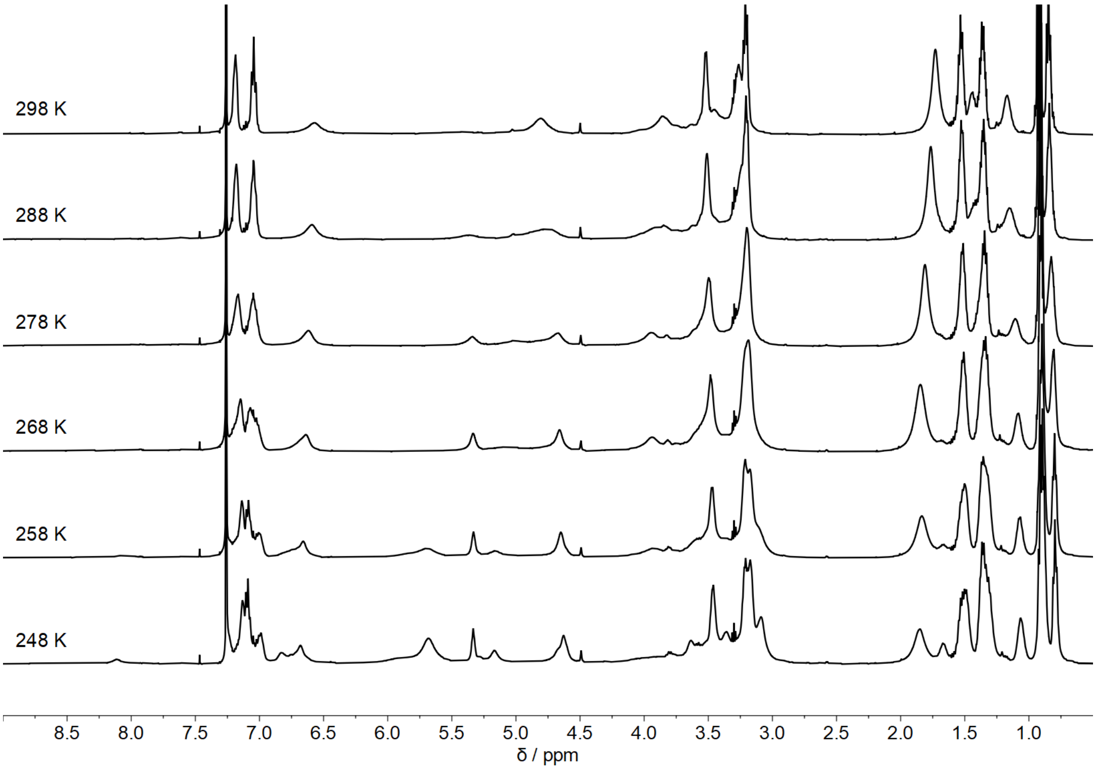

Figure S20. 500 MHz ^{1}H VT NMR of **26** (8 mM) in $CDCl_3$.

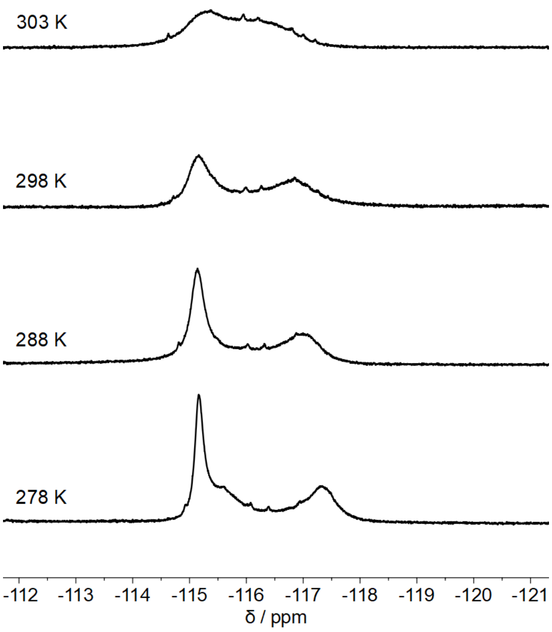

Figure S21. 565 MHz ^{19}F VT NMR of **26** (8 mM) in $CDCl_3$.

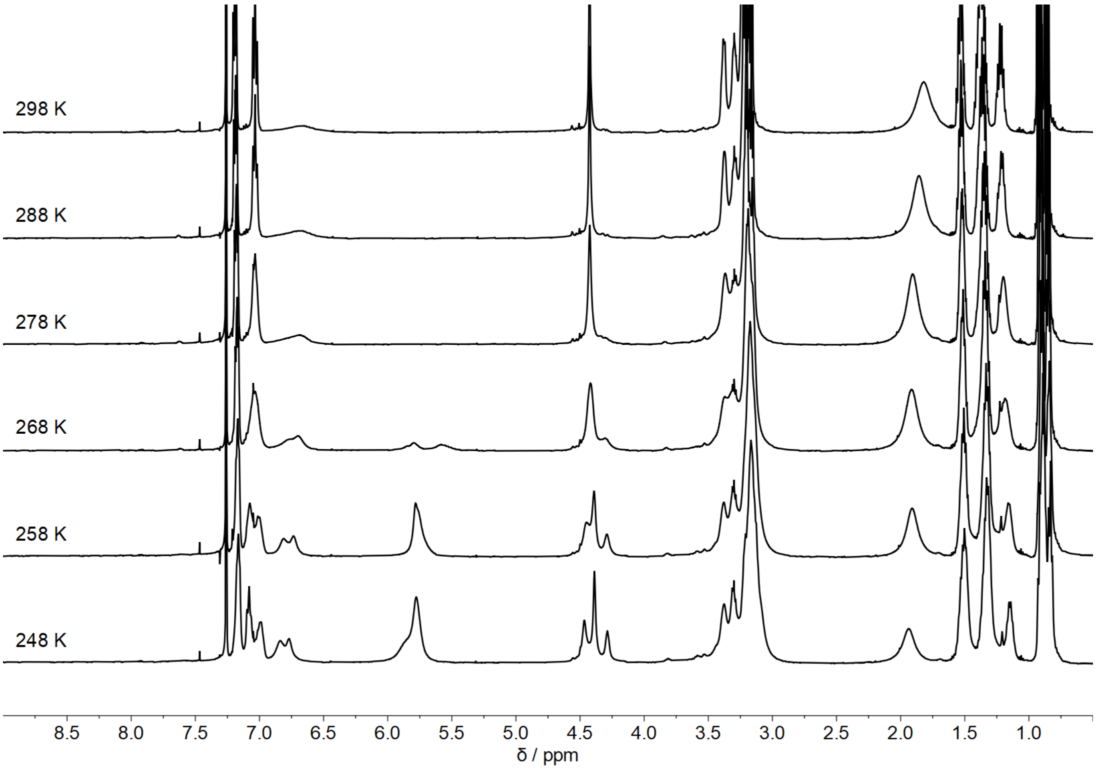

Figure S22. 500 MHz ¹H VT NMR of **27** (8 mM) in $CDCl_3$.

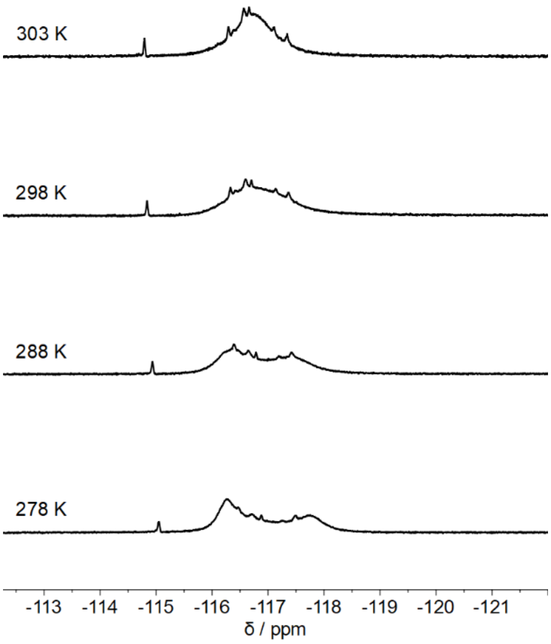

Figure S23. 565 MHz ¹⁹F VT NMR of **27** (8 mM) in $CDCl_3$.

S4.3 2D NMR in $CDCl_3$ at 248 K

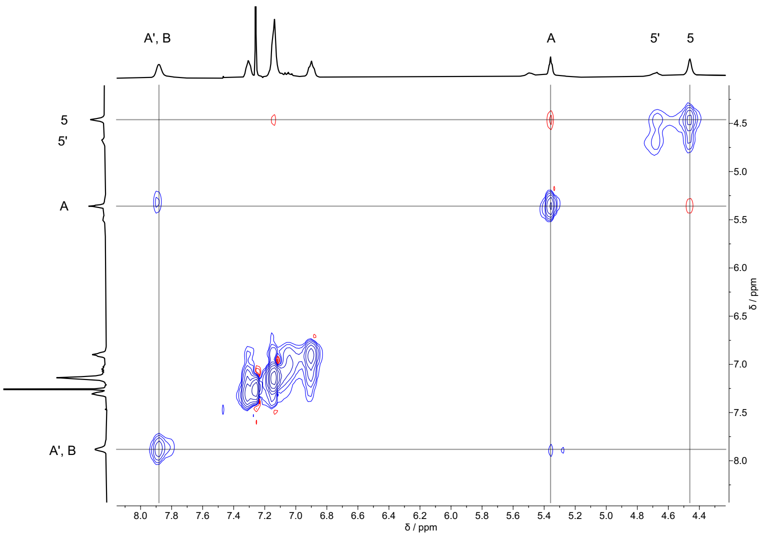

Figure S24. 500 MHz ^1H-^1H ROESY of **3** in $CDCl_3$ at 248 K.

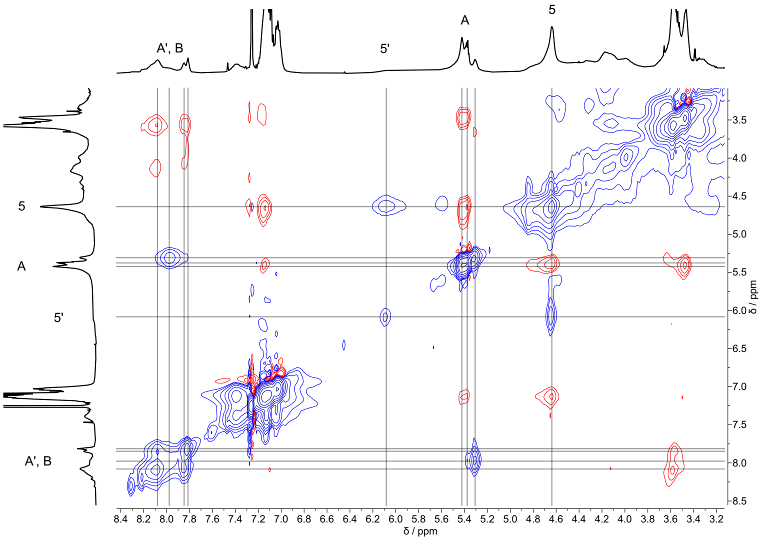

Figure S25. 500 MHz ^1H-^1H ROESY of **4** in $CDCl_3$ at 248 K.

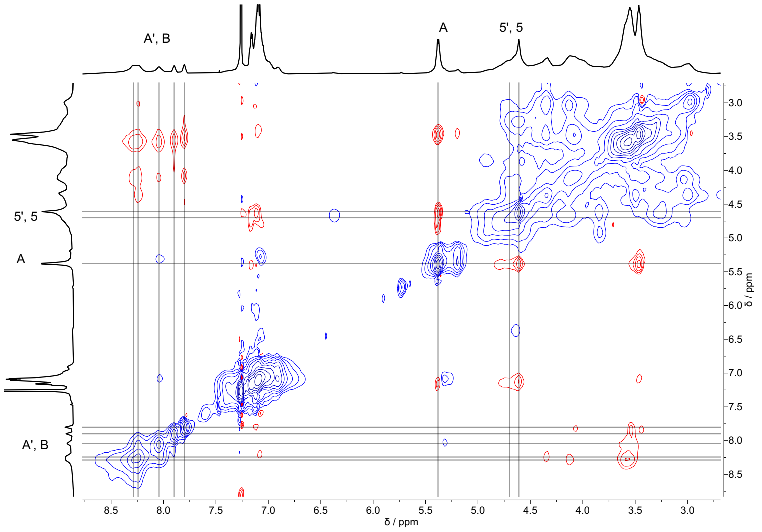

Figure S26. 500 MHz ^1H-^1H ROESY of **5** in $CDCl_3$ at 248 K.

S4.4 Assignment of ^{19}F NMR Spectrum of 3

Compound **3** exists in equilibrium between two conformers with opposite H-bond directionality (Figure S27). The following NMR spectra in Figures S28-S34 were used to assign the ^{1}H signals of the NH moieties and of the capping groups at the *N*- and *C*-terminus, and the ^{19}F peaks at the *N*- and *C*-terminus.

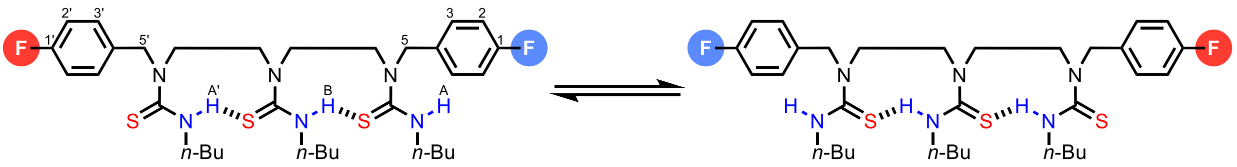

Figure S27. Equilibrium between the two conformers of **3** with labels for NMR assignments.

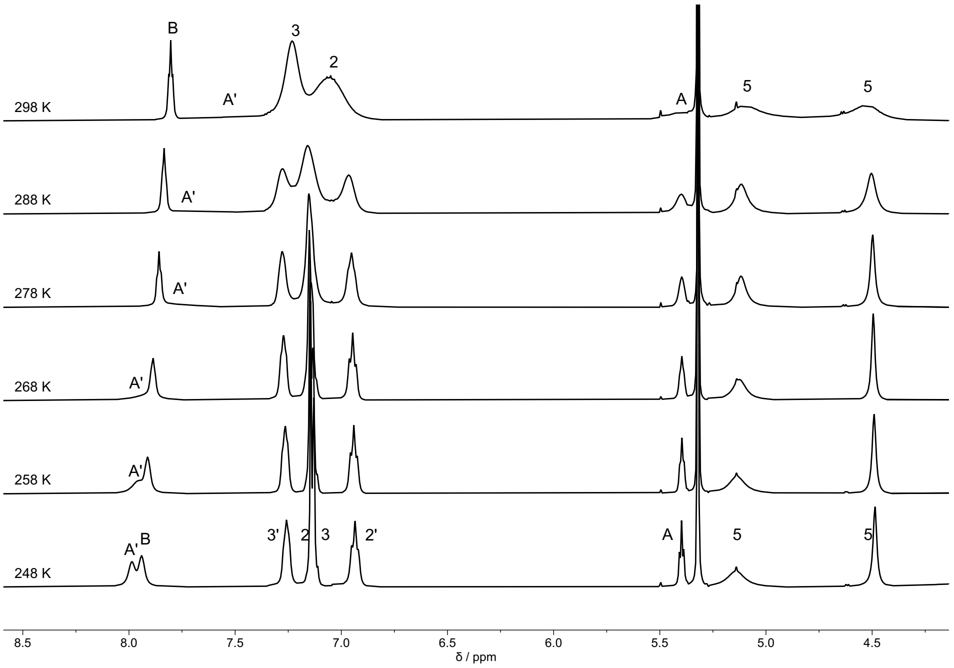

Figure S28. 500 MHz ^{1}H VT NMR of **3** (10 mM) in CD_2Cl_2 from 298 K to 248 K.

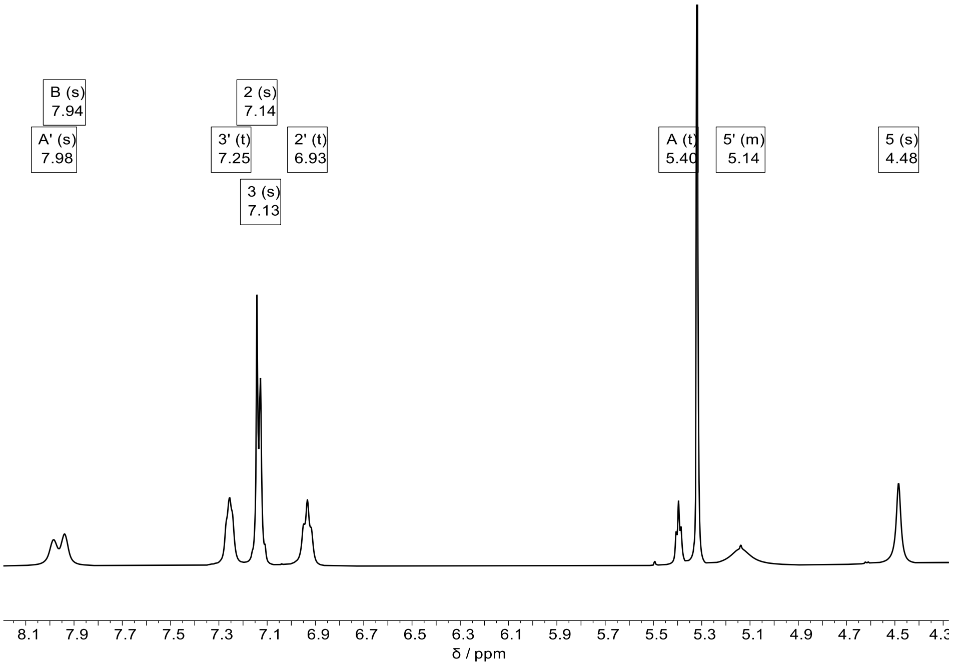

Figure S29. 500 MHz ^{1}H NMR of **3** (10 mM) in CD_2Cl_2 at 248 K.

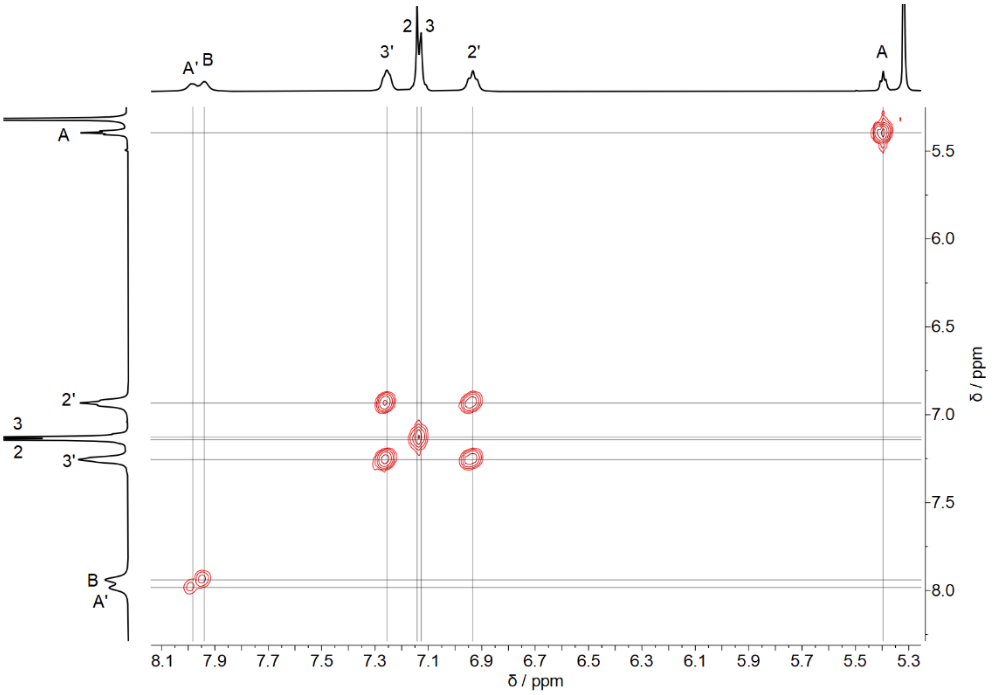

Figure S30. 500 MHz ^{1}H-^{1}H COSY spectrum of **3** (10 mM) in CD_2Cl_2 at 248 K.

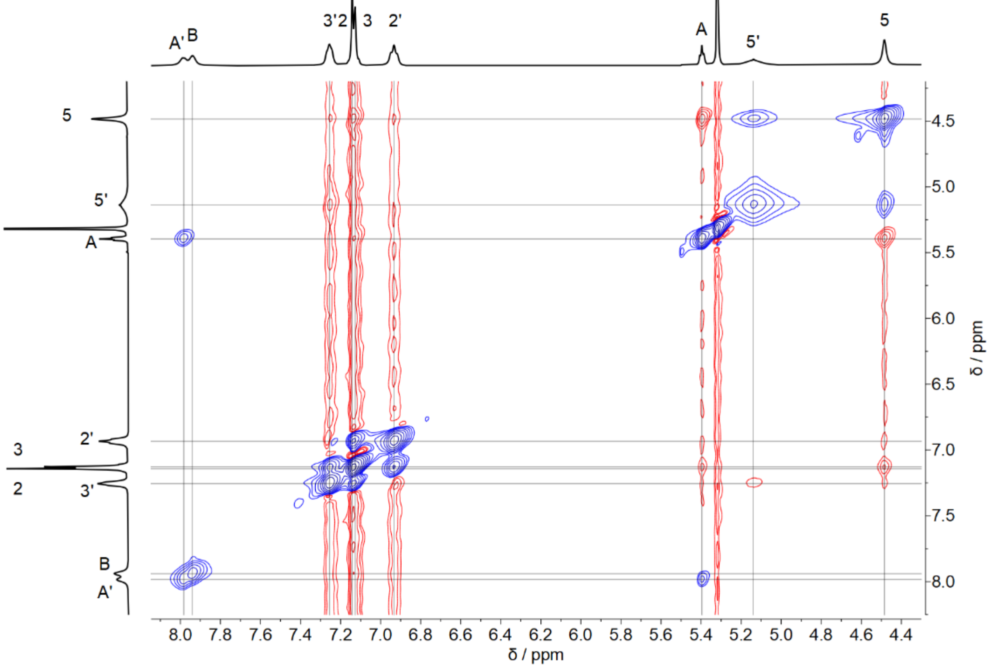

Figure S31. 500 MHz ^{1}H-1H ROESY spectrum of **3** (10 mM) in CD_2Cl_2 at 248 K.

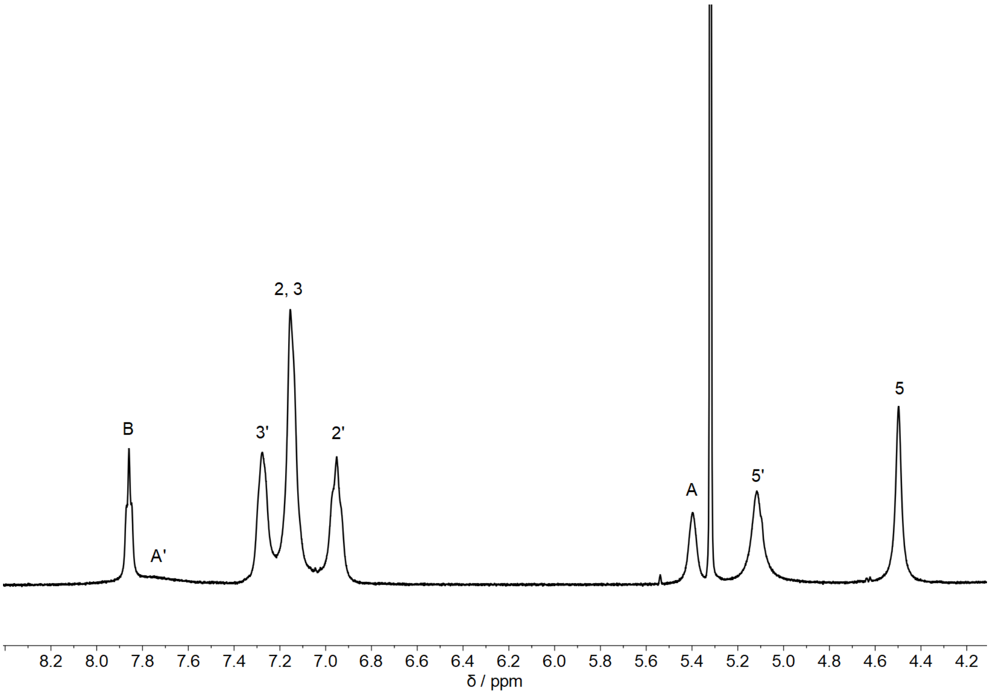

Figure S32. 400 MHz ^{1}H NMR of **3** (10 mM) in CD_2Cl_2 at 278 K.

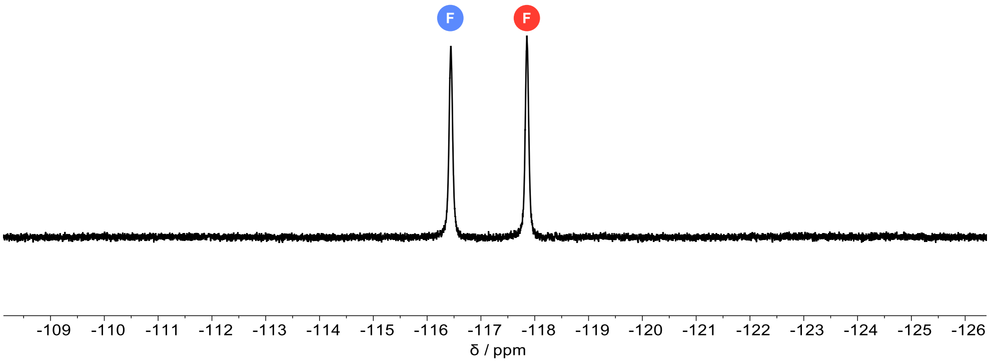

Figure S33. 376 MHz ^{19}F NMR of **3** (10 mM) in CD_2Cl_2 at 278 K.

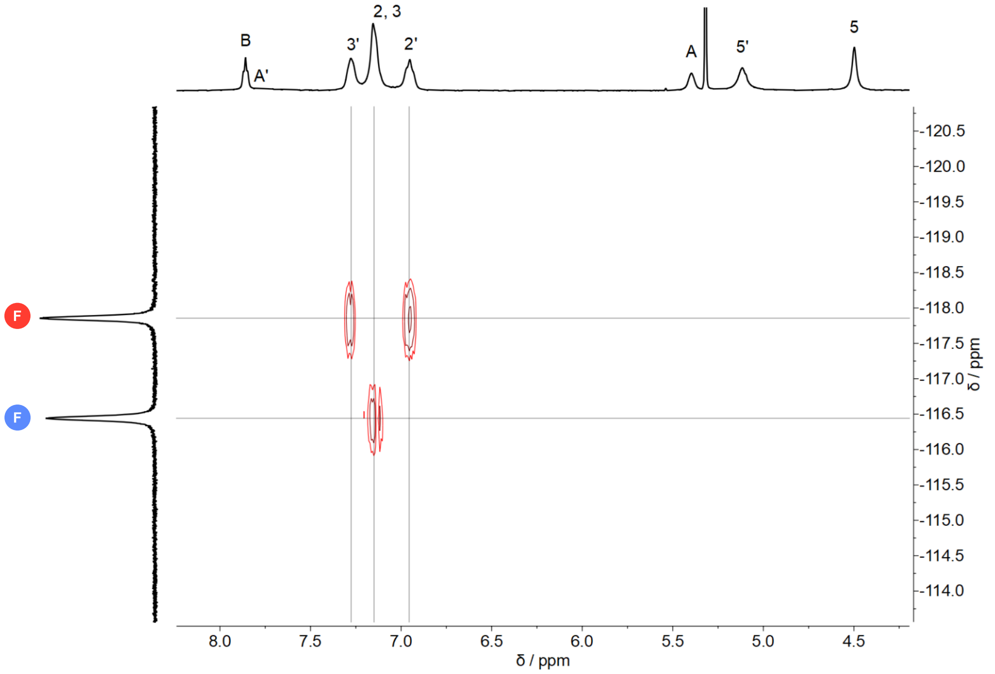

Figure S34. 400 MHz ^{1}H-^{19}F HMBC of **3** (10 mM) in CD_2Cl_2 at 278 K.

S4.5 VT NMR in micelles

Note: At 278 K a precipitate formed for sample of foldamers incorporated in SDS micelles.

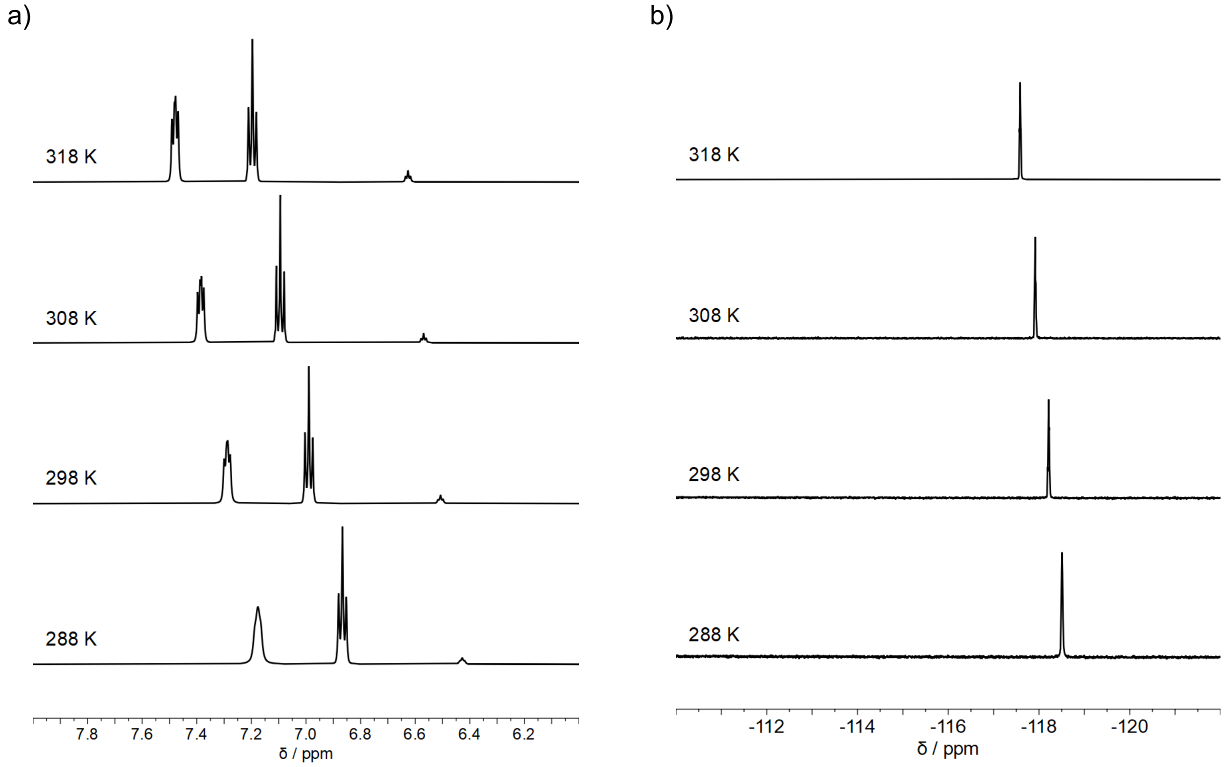

Figure S35. (a) 600 MHz ^{1}H (with presaturation[6] for the suppression of the water signal) and (b) 565 MHz ^{19}F VT NMR of **1** (1 mM) embedded in micelles (SDS, 200 mM) in MOPS (20 mM, pH 7.4), NaCl (100 mM), KF (0.05 mM).

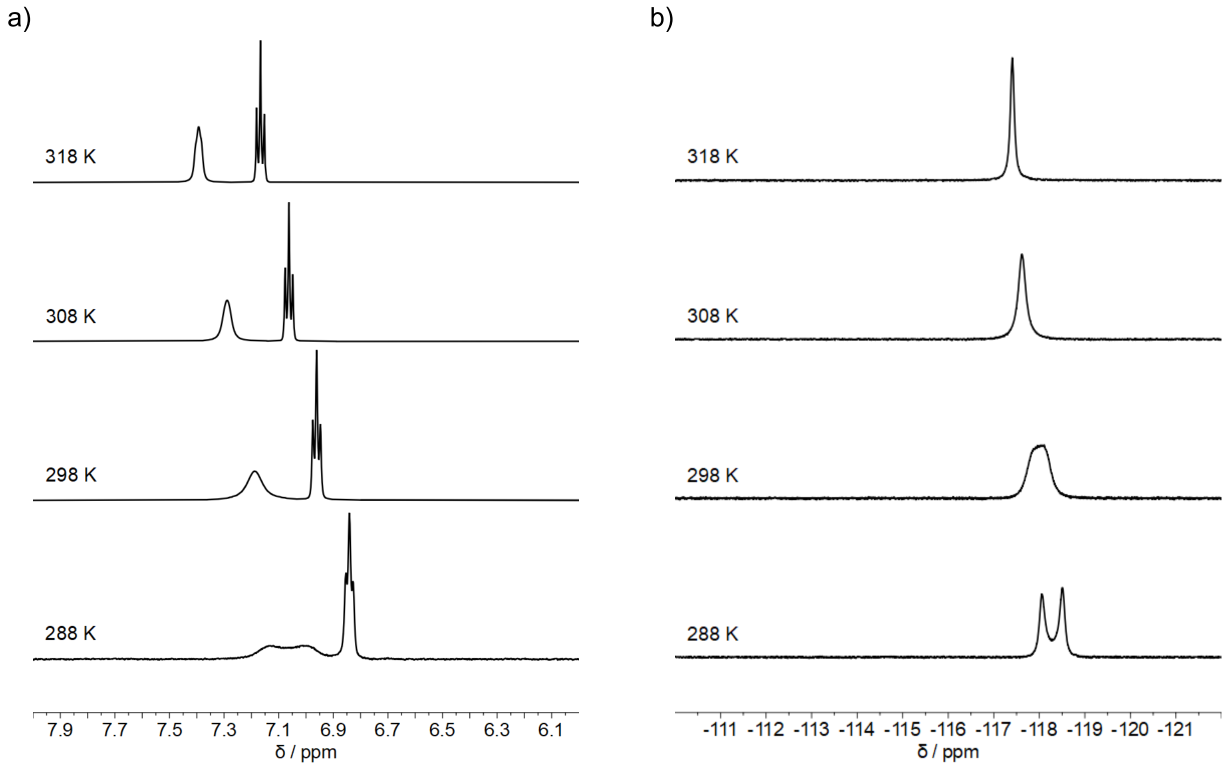

Figure S36. (a) 600 MHz ^{1}H (with presaturation[6] for the suppression of the water signal) and (b) 565 MHz ^{19}F VT NMR of **2** (1 mM) embedded in micelles (SDS, 200 mM) in MOPS (20 mM, pH 7.4), NaCl (100 mM), KF (0.05 mM).

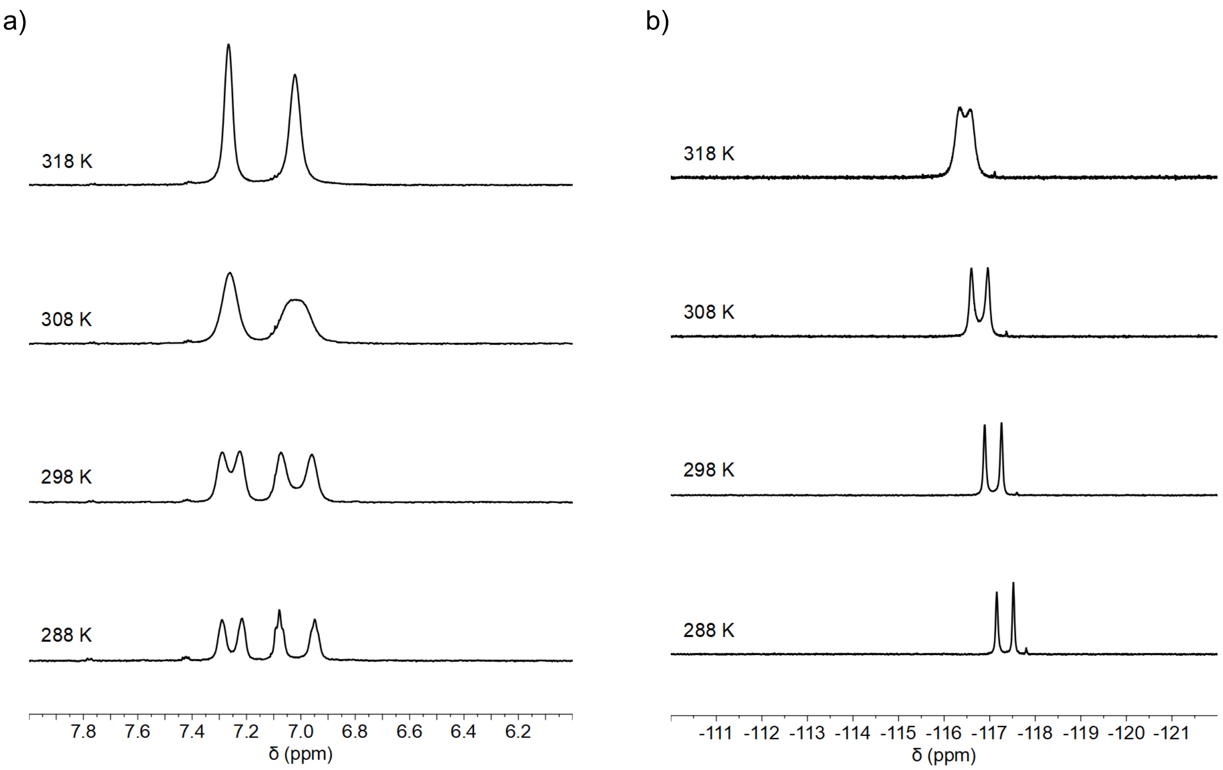

Figure S37. (a) 600 MHz ^{1}H (with presaturation[6] for the suppression of the water signal) and (b) 565 MHz ^{19}F VT NMR of **3** (1 mM) embedded in micelles (SDS, 200 mM) in MOPS (20 mM, pH 7.4), NaCl (100 mM), KF (0.05 mM).

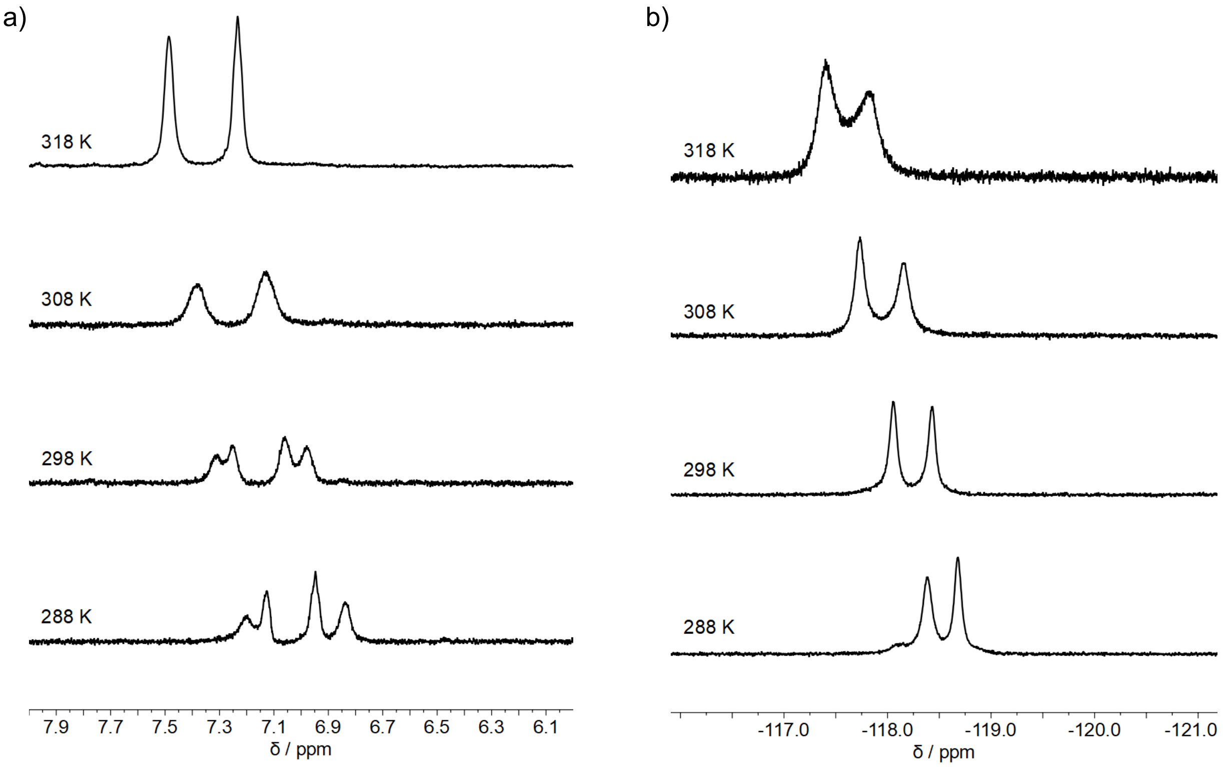

Figure S38. (a) 600 MHz ^{1}H (with presaturation[6] for the suppression of the water signal) and (b) 565 MHz ^{19}F VT NMR of **4** (1 mM) embedded in micelles (SDS, 200 mM) in MOPS (20 mM, pH 7.4), NaCl (100 mM), KF (0.05 mM).

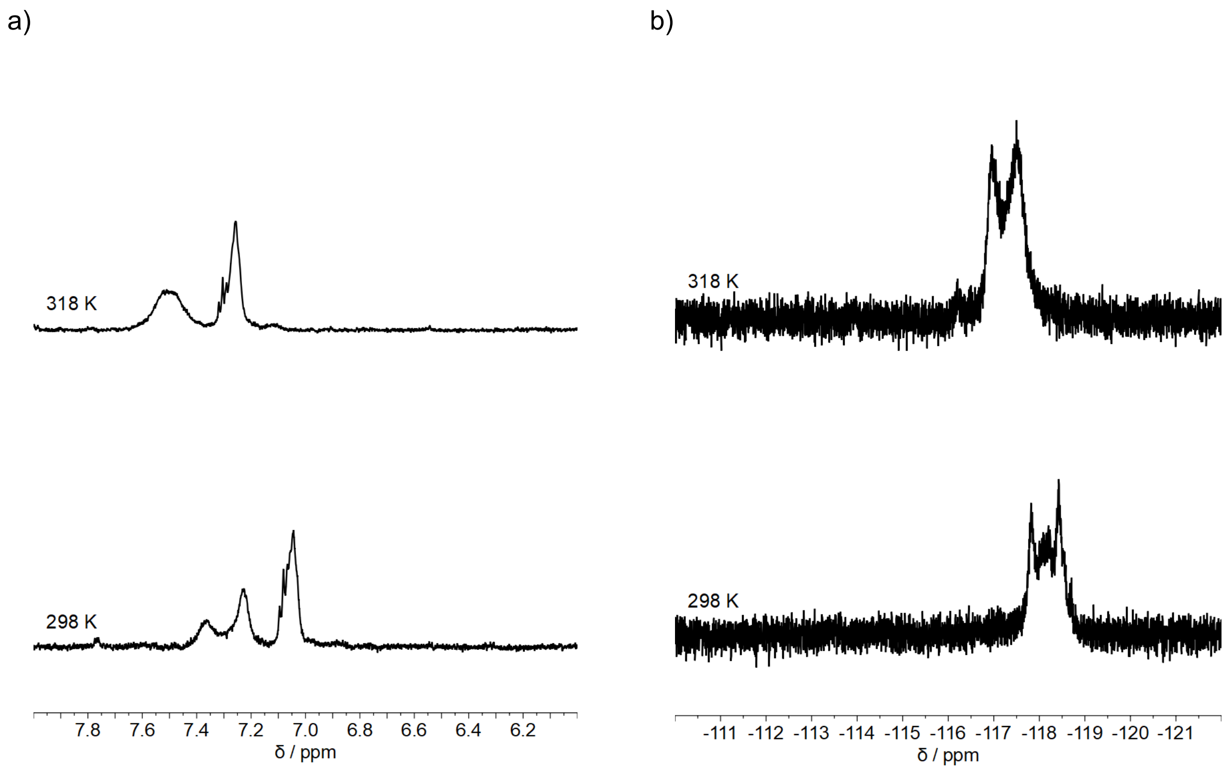

Figure S39. (a) 600 MHz ^{1}H (with presaturation[6] for the suppression of the water signal) and (b) 565 MHz ^{19}F VT NMR of **5** (1 mM) embedded in micelles (SDS, 200 mM) in MOPS (20 mM, pH 7.4), NaCl (100 mM), KF (0.05 mM).

S4.6 VT NMR in bicelles

Note: The temperature-dependent changes in chemical shift and line width observed for compounds **3-5** in bicelles are likely due to gradual changes in the local membrane environment, leading to slight broadening and peak shifts, rather than exchange-driven coalescence.

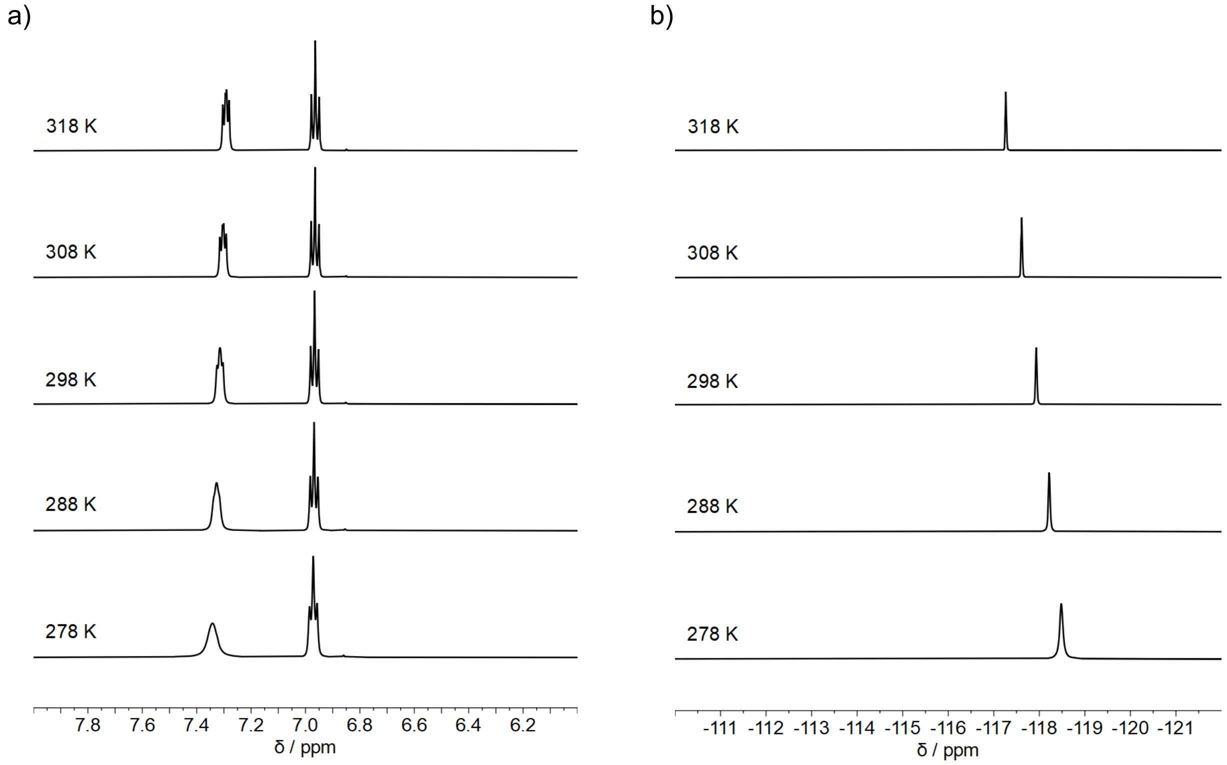

Figure S40. (a) 600 MHz ^{1}H (with presaturation[6] for the suppression of the water signal) and (b) 565 MHz ^{19}F VT NMR of **1** (1 mM) embedded in bicelles (DLPC:DHPC, 300 mM, q = 0.5) in MOPS (20 mM, pH 7.4), NaCl (100 mM), KF (0.05 mM).

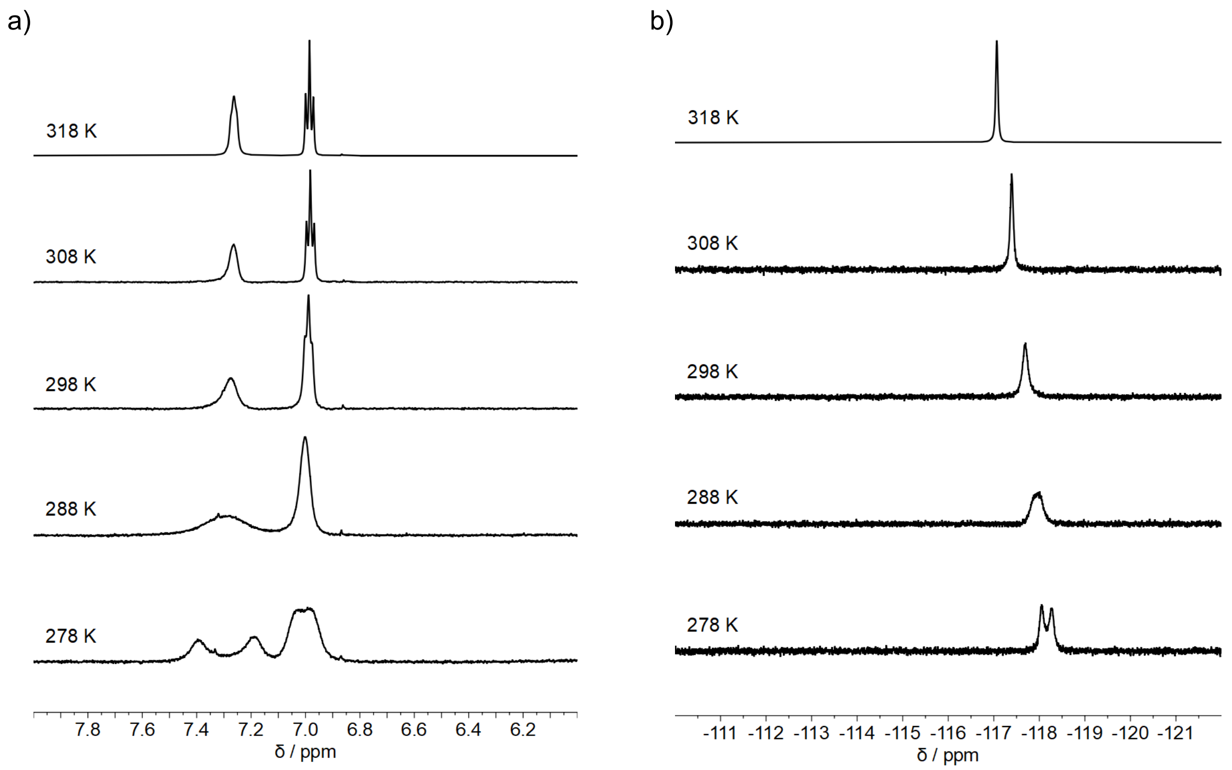

Figure S41. (a) 600 MHz ^{1}H (with presaturation[6] for the suppression of the water signal) and (b) 565 MHz ^{19}F VT NMR of **2** (1 mM) embedded in bicelles (DLPC:DHPC, 300 mM, q = 0.5) in MOPS (20 mM, pH 7.4), NaCl (100 mM), KF (0.05 mM).

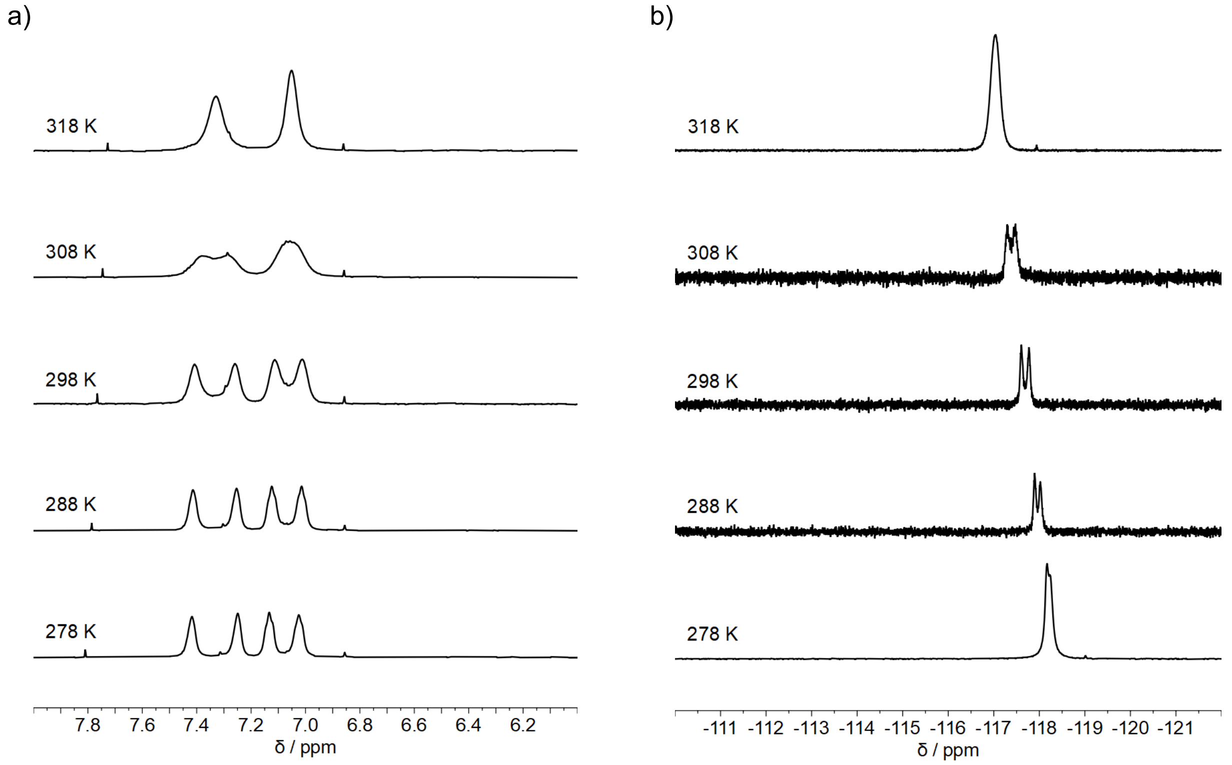

Figure S42. (a) 600 MHz ^{1}H (with presaturation[6] for the suppression of the water signal) and (b) 565 MHz ^{19}F VT NMR of **3** (1 mM) embedded in bicelles (DLPC:DHPC, 300 mM, q = 0.5) in MOPS (20 mM, pH 7.4), NaCl (100 mM), KF (0.05 mM).

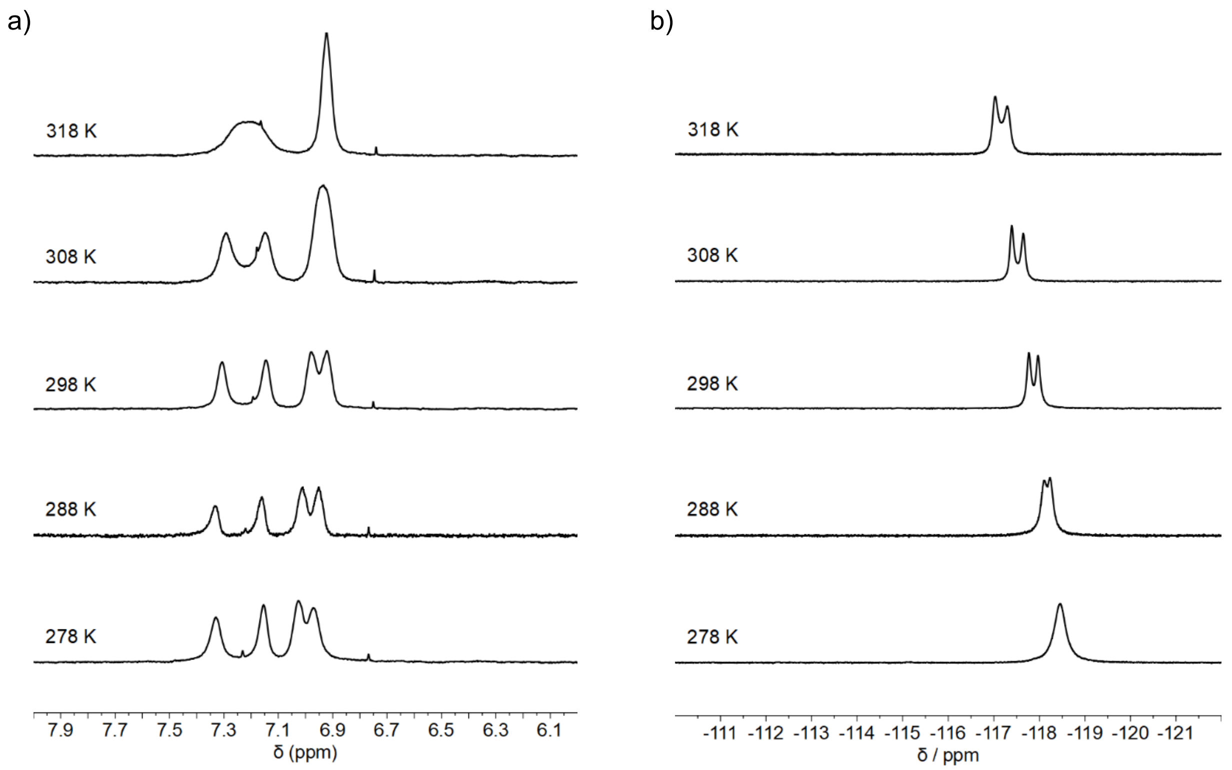

Figure S43. (a) 600 MHz ^{1}H (with presaturation[6] for the suppression of the water signal) and (b) 565 MHz ^{19}F VT NMR of **4** (1 mM) embedded in bicelles (DLPC:DHPC, 300 mM, q = 0.5) in MOPS (20 mM, pH 7.4), NaCl (100 mM), KF (0.05 mM).

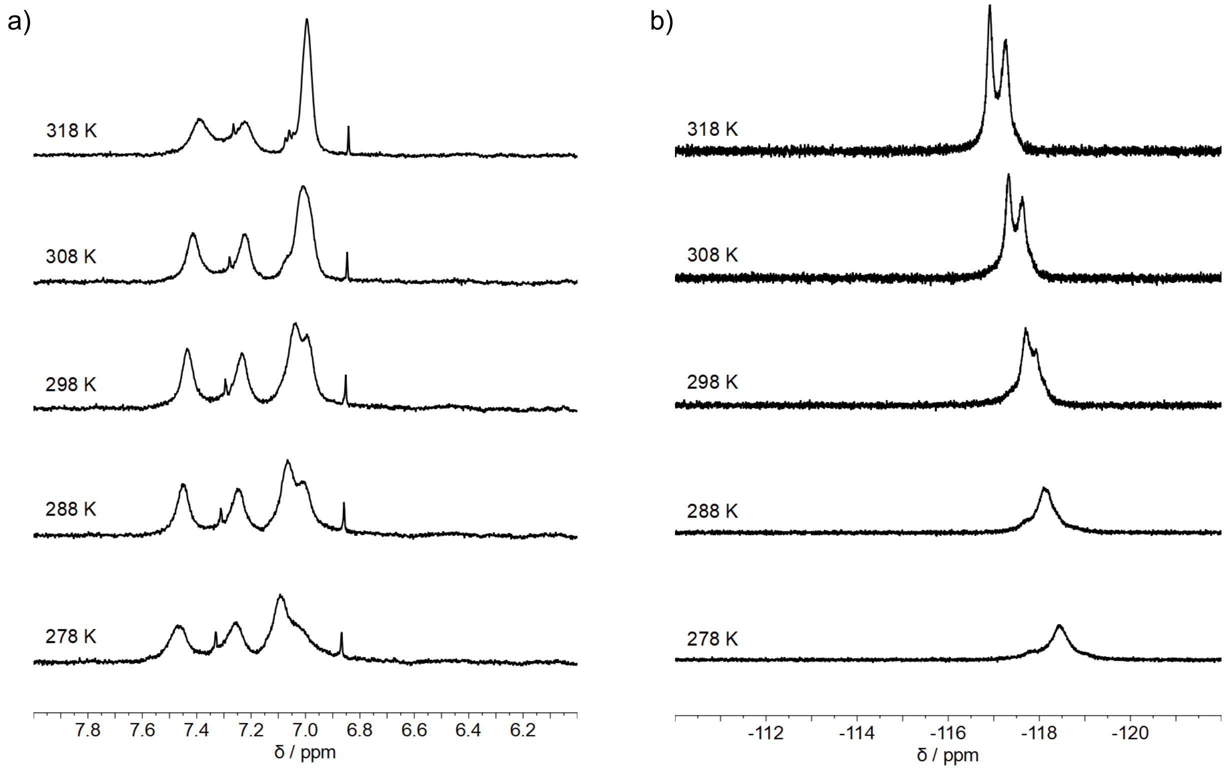

Figure S44. (a) 600 MHz ^{1}H (with presaturation[6] for the suppression of the water signal) and (b) 565 MHz ^{19}F VT NMR of **5** (1 mM) embedded in bicelles (DLPC:DHPC, 300 mM, q = 0.5) in MOPS (20 mM, pH 7.4), NaCl (100 mM), KF (0.05 mM).

S4.7 Lineshape Analysis of VT NMR of 2 and 3 in CD_2Cl_2, micelles and bicelles

^{19}F NMR spectra of **2** in CD_2Cl_2, in micelles and in bicelles were acquired around the respective decoalescence temperature at 2 K intervals (Figures S45, S47, S49). Line shape fitting of the experimental spectra at different temperatures using the Topspin 4.3.0 Dynamic NMR Models tool was used to calculate the rate constants of H-bond directionality reversal at each temperature (Figures S46, S48, S50). The Eyring-Polanyi equation was rearranged to give the equation of a straight line:

$$\ln\left(\frac{k}{T}\right) = -\frac{\Delta H^{\ddagger}}{RT} + \frac{\Delta S^{\ddagger}}{R} + \ln\left(\frac{k_B}{h}\right) \tag{1}$$

with

$$slope = -\frac{\Delta H^{\ddagger}}{R} \tag{2}$$

and

$$intercept = \frac{\Delta S^{\ddagger}}{R} + \ln\left(\frac{k_B}{h}\right) \tag{3}$$

where k is the exchange rate constant, T is the temperature in Kelvin, $\Delta H^{\ddagger}$ and $\Delta S^{\ddagger}$ are respectively the enthalpy and the entropy associated with the barrier to the H-bond polarity reversal, R is the gas constant (8.3145 J mol^{-1} K^{-1}), k_B is the Boltzmann constant (1.380649 × 10^{-23} m^2 kg s^{-2} K^{-1}) and h is the Planck constant (6.62607015 × 10^{-34} m^2 kg s^{-1}).[7] Uncertainty of $\Delta H^{\ddagger}$ and $\Delta S^{\ddagger}$ are the standard error from the Eyring plot uncertainty in slope and intercept.

The $\Delta H^{\ddagger}$ and $\Delta S^{\ddagger}$ values obtained from the fittings were used in the following equation

$$\Delta G^{\ddagger} = \Delta H^{\ddagger} - T\Delta S^{\ddagger} \tag{4}$$

to calculate the Gibbs free energy $\Delta G^{\ddagger}$ at 298 K.[7] $\Delta G^{\ddagger}$ uncertainty was calculated through error propagation.

The experimentally obtained values for $\Delta H^{\ddagger}, \Delta S^{\ddagger}$ and $\Delta G^{\ddagger}$ with the corresponding errors are summarised in Table S5.

**2** in	$\Delta H^{\ddagger}$ / kJ mol^{-1}	$\Delta S^{\ddagger}$ / J mol^{-1} K^{-1}	$\Delta G^{\ddagger}$ / kJ mol^{-1}
CD_2Cl_2	68.7 ± 3.0	58 ± 11	51.5 ± 4.3
micelles	83.4 ± 4.9	89 ± 17	56.7 ± 6.9
bicelles	76.5 ± 3.3	69 ± 11	55.9 ± 4.8

Table S5. $\Delta H^{\ddagger}, \Delta S^{\ddagger}$ and $\Delta G^{\ddagger}$ obtained from lineshape analysis and fitting with the Eyring-Polanyi equation for compound **2** in CD_2Cl_2, micelles and bicelles.

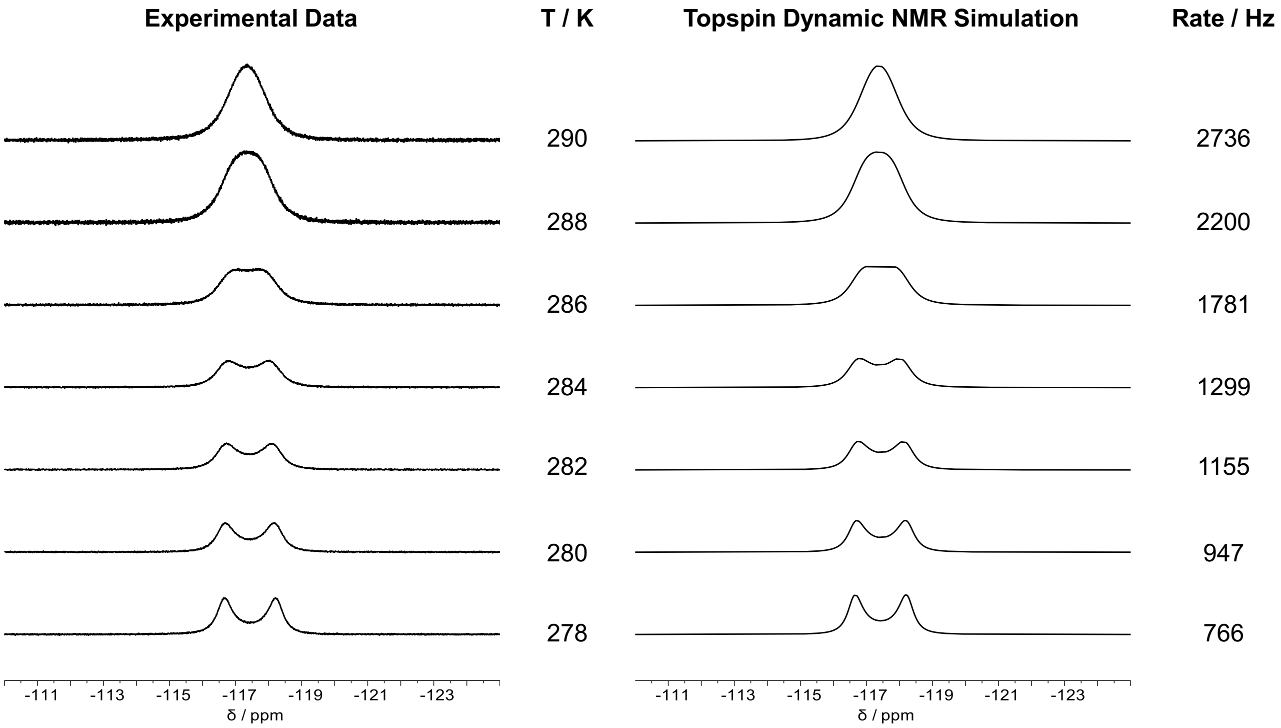

Figure S45. Experimental (on the left) and simulated (on the right) ^{19}F VT NMR spectra of **2** in CD_2Cl_2.

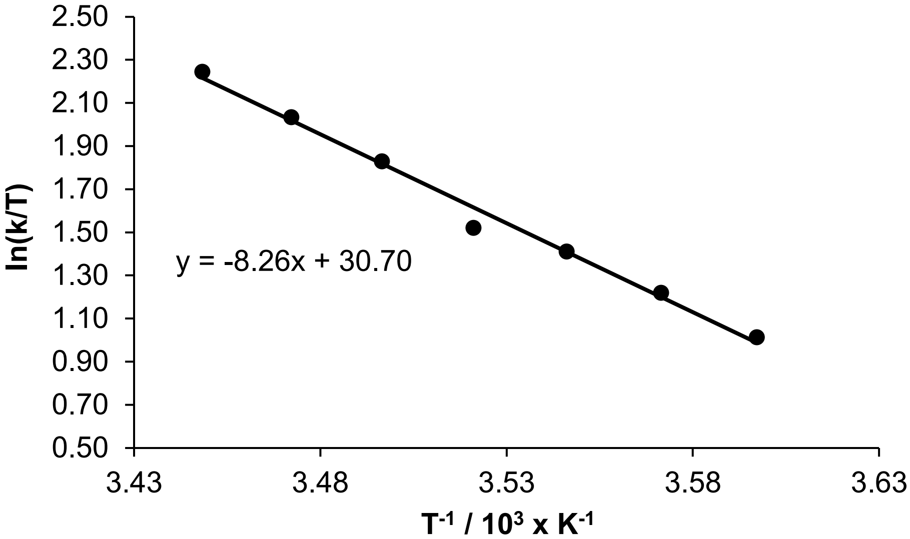

Figure S46. Eyring plot of **2** in CD_2Cl_2 ($\Delta G^{\ddagger}_{298\,K}$ = 51.5 ± 4.3 kJ mol^{-1}).

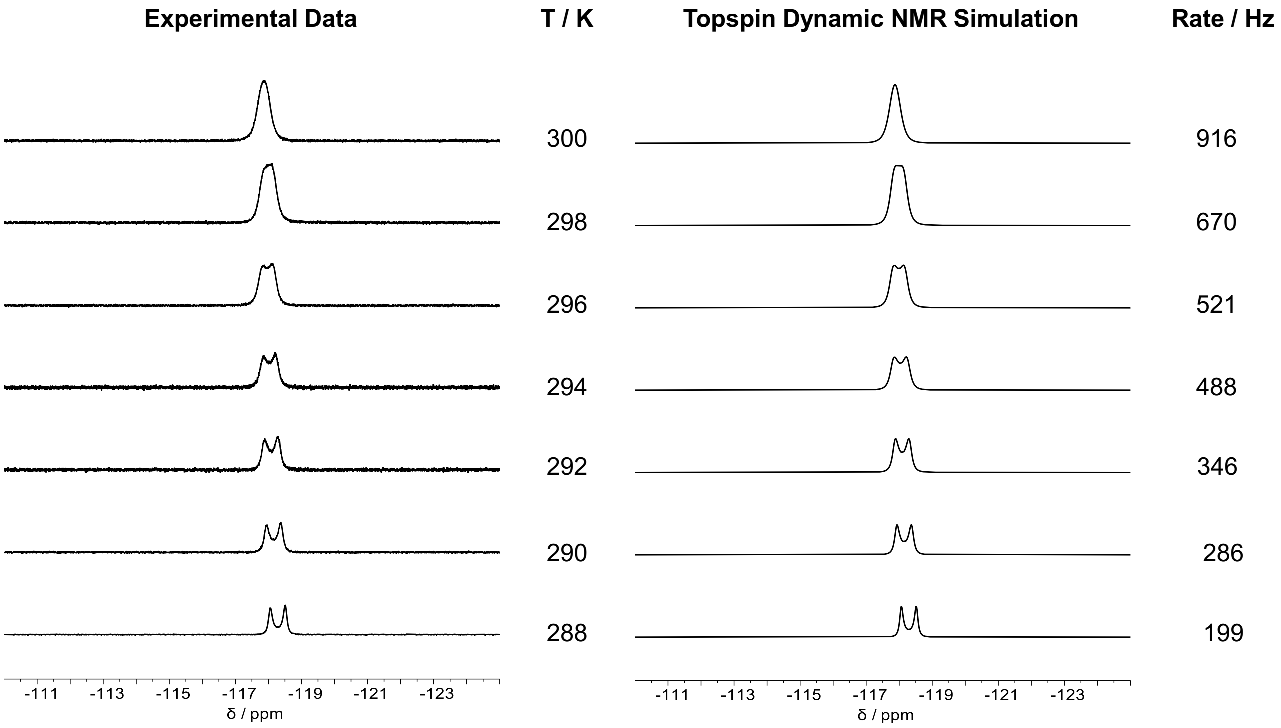

Figure S47. Experimental (on the left) and simulated (on the right) ^{19}F VT NMR spectra of **2** (1 mM) embedded in micelles (SDS, 200 mM) in MOPS (20 mM, pH 7.4), NaCl (100 mM), KF (0.05 mM).

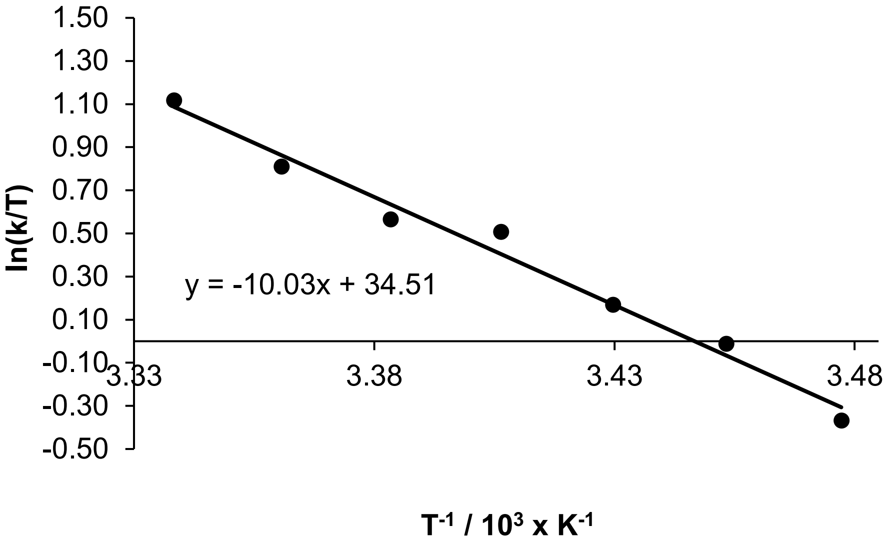

Figure S48. Eyring plot of **2** in micelles ($\Delta G^{\ddagger}_{298\,K}$ = 56.7 ± 6.9 kJ mol^{-1}).

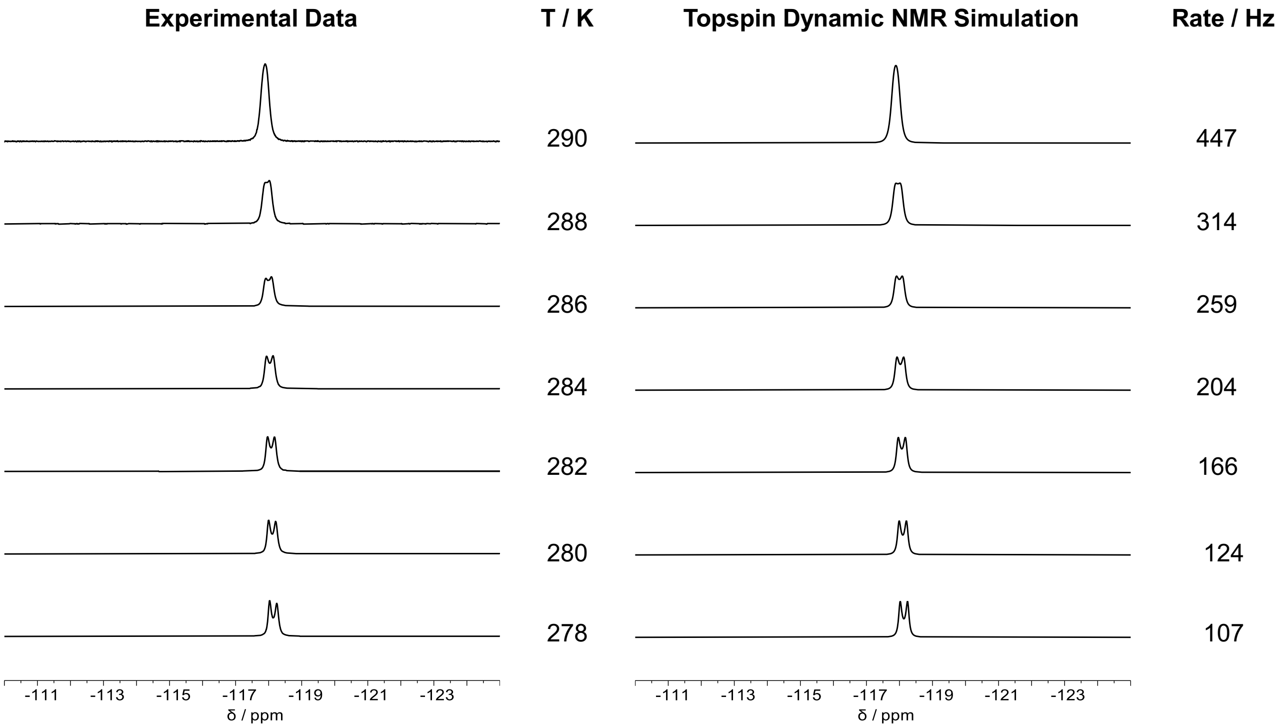

Figure S49. Experimental (on the left) and simulated (on the right) ^{19}F VT NMR spectra of **2** (1 mM) embedded in bicelles (DLPC:DHPC, 300 mM, q = 0.5) in MOPS (20 mM, pH 7.4), NaCl (100 mM), KF (0.05 mM).

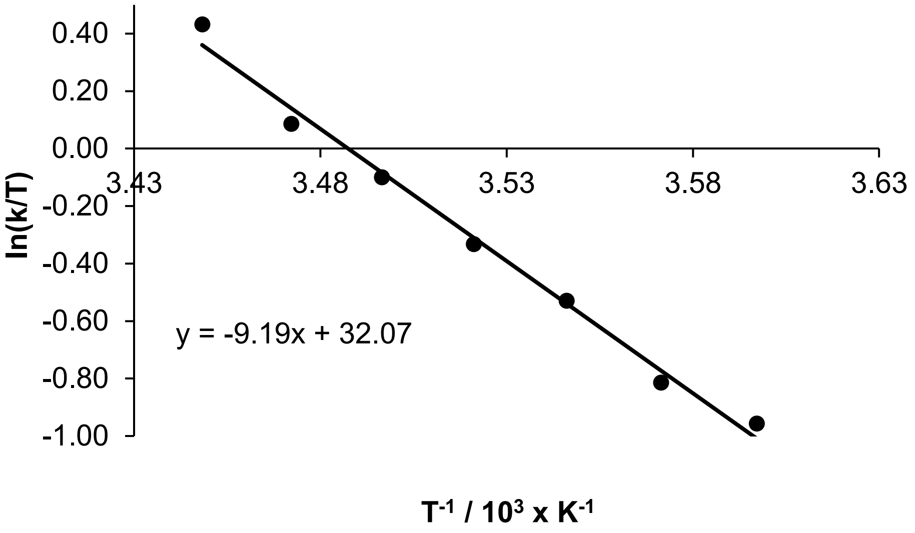

Figure S50. Eyring plot of **2** in bicelles ($\Delta G^{\ddagger}_{298\ K}$ = 55.9 ± 4.8 kJ mol^{-1}).

S4.8 Insolubility of Foldamers in Aqueous Buffer

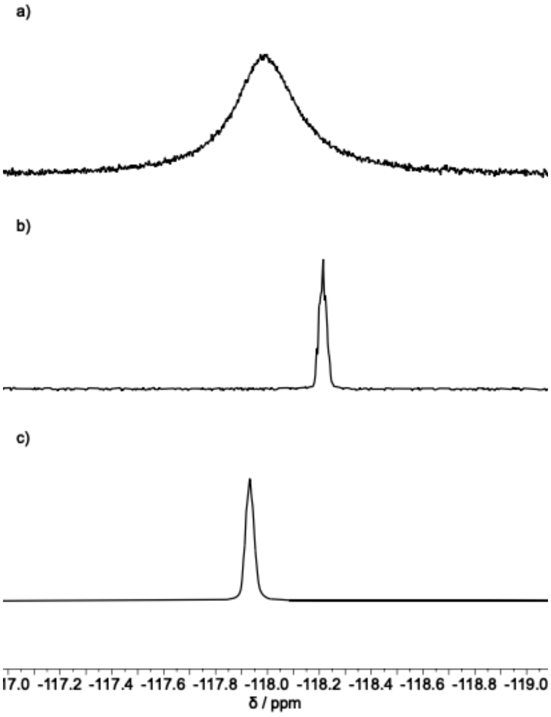

Figure S51. ^{19}F NMR spectra of **1** (1 mM, in 9:1 MeOD/d_6-DMSO) added to (a) an aqueous solution of MOPS (20 mM, pH 7.4), NaCl (100 mM), KF (0.05 mM), (b) micelles (SDS, 200 mM) in MOPS (20 mM, pH 7.4), NaCl (100 mM), KF (0.05 mM), and (c) bicelles (DLPC:DHPC, 300 mM, q = 0.5) in MOPS (20 mM, pH 7.4), NaCl (100 mM), KF (0.05 mM).

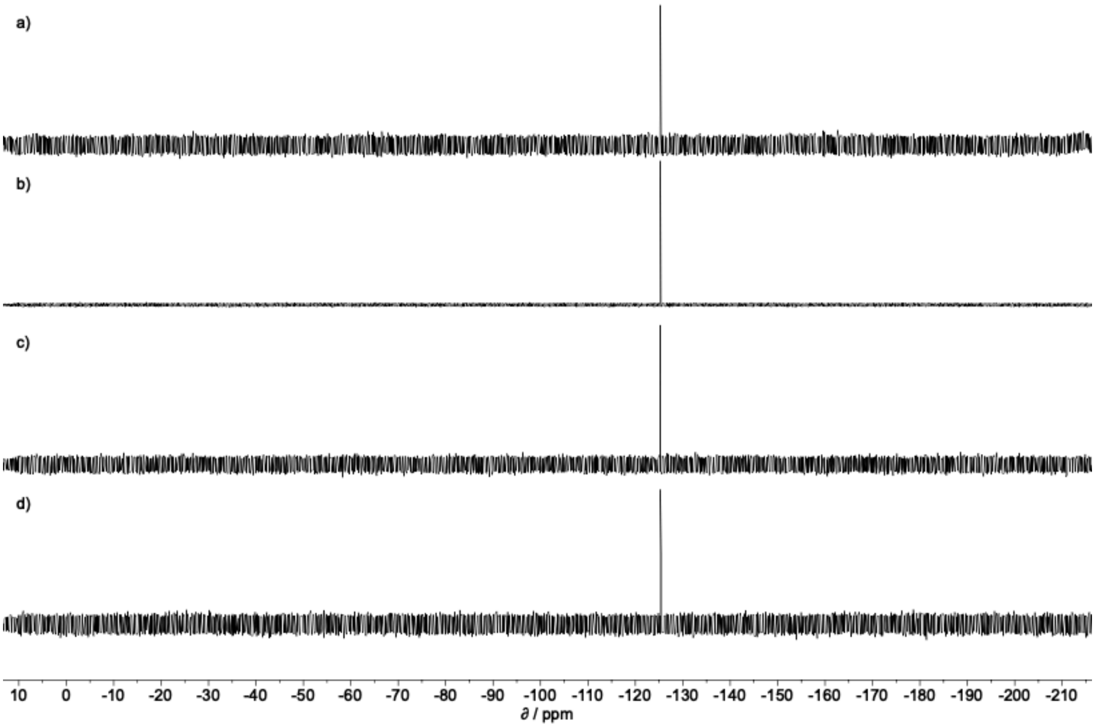

Figure S52. ^{19}F NMR spectra of (a) **2**, (b) **3**, (c) **4**, (d) **5** (1 mM, in 9:1 MeOD/d_6-DMSO) added to an aqueous solution of MOPS (20 mM, pH 7.4), NaCl (100 mM), KF (0.05 mM). The peak at -123.3 ppm is of F^-.

S4.9 Foldamers 24-27 in $CDCl_3$, vesicles, micelles and bicelles

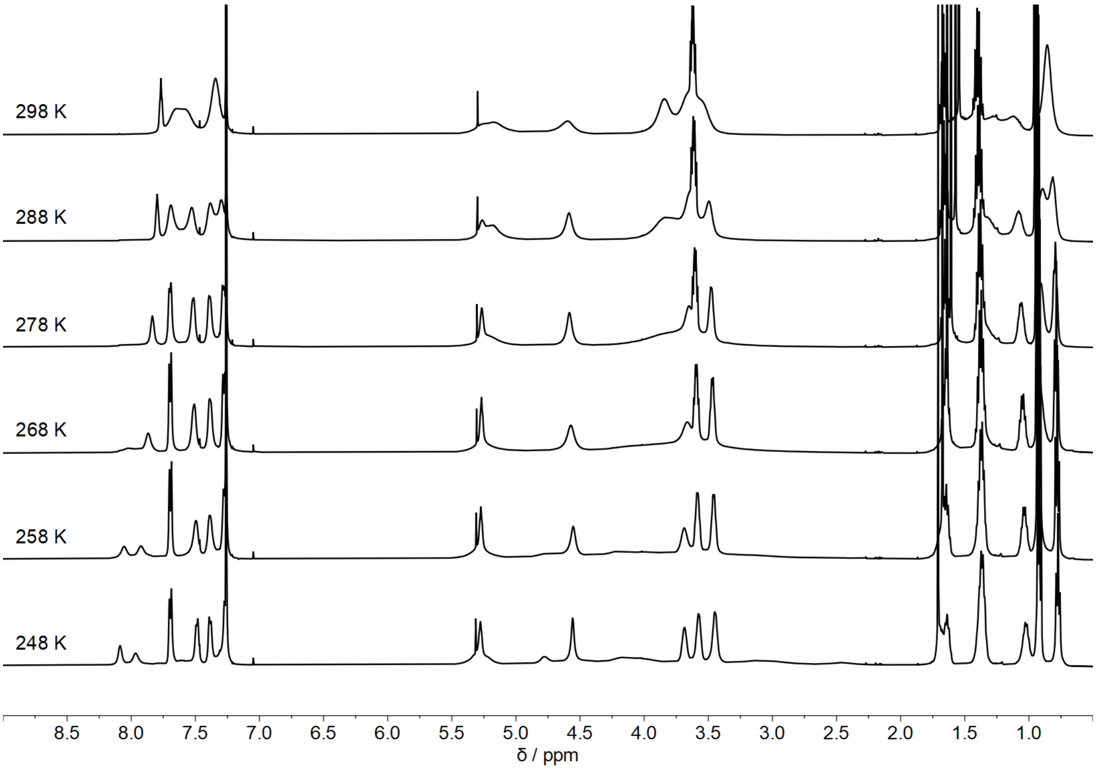

Figure S53. 500 MHz ^{1}H VT NMR of **24** (8 mM) in $CDCl_3$.

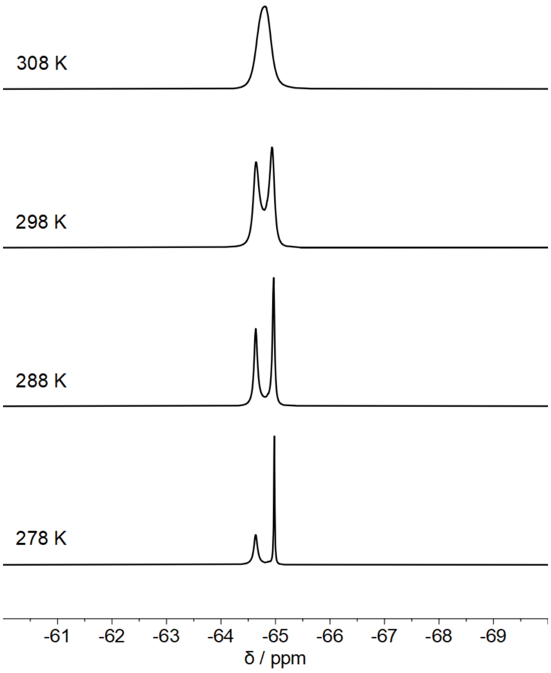

Figure S54. 565 MHz ^{19}F VT NMR of **24** (8 mM) in $CDCl_3$.

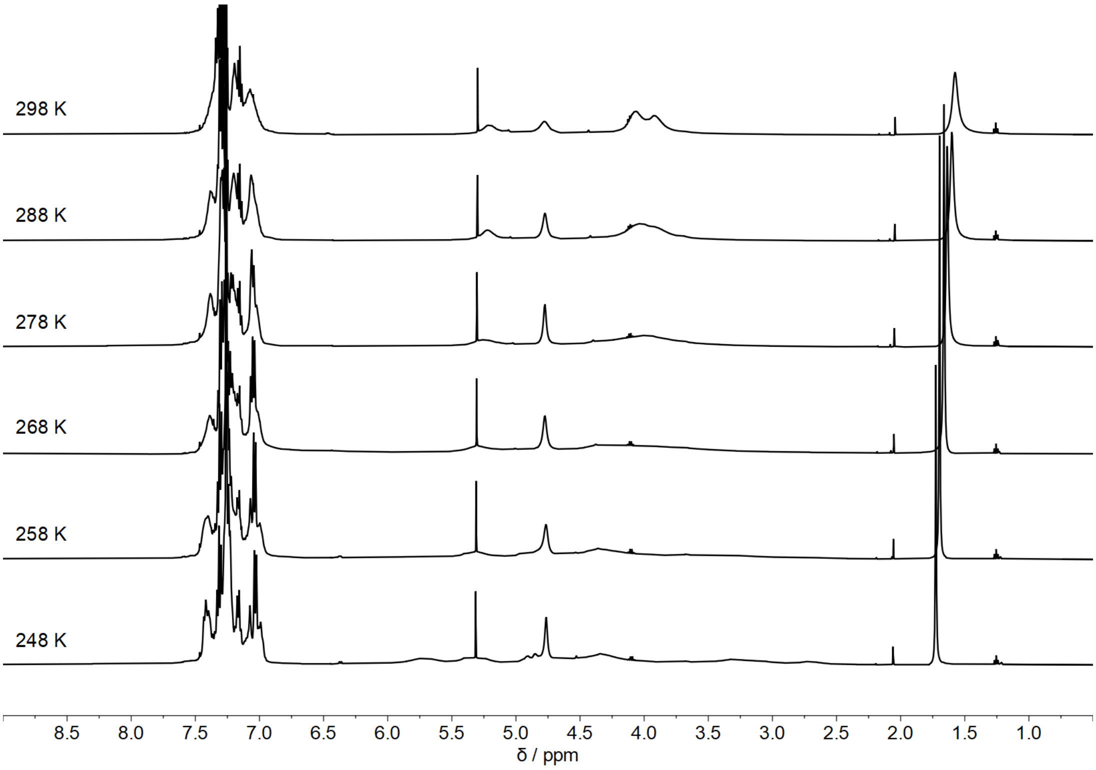

Figure S55. 500 MHz ^{1}H VT NMR of **25** (8 mM) in $CDCl_3$.

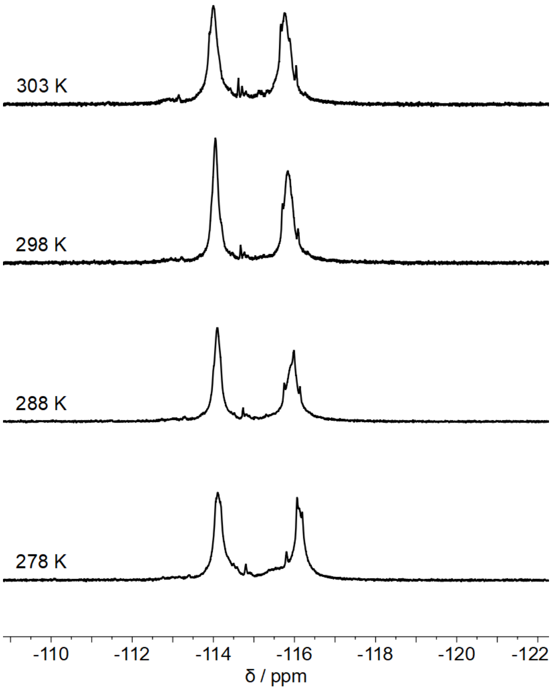

Figure S56. 565 MHz ^{19}F VT NMR of **25** (8 mM) in $CDCl_3$.

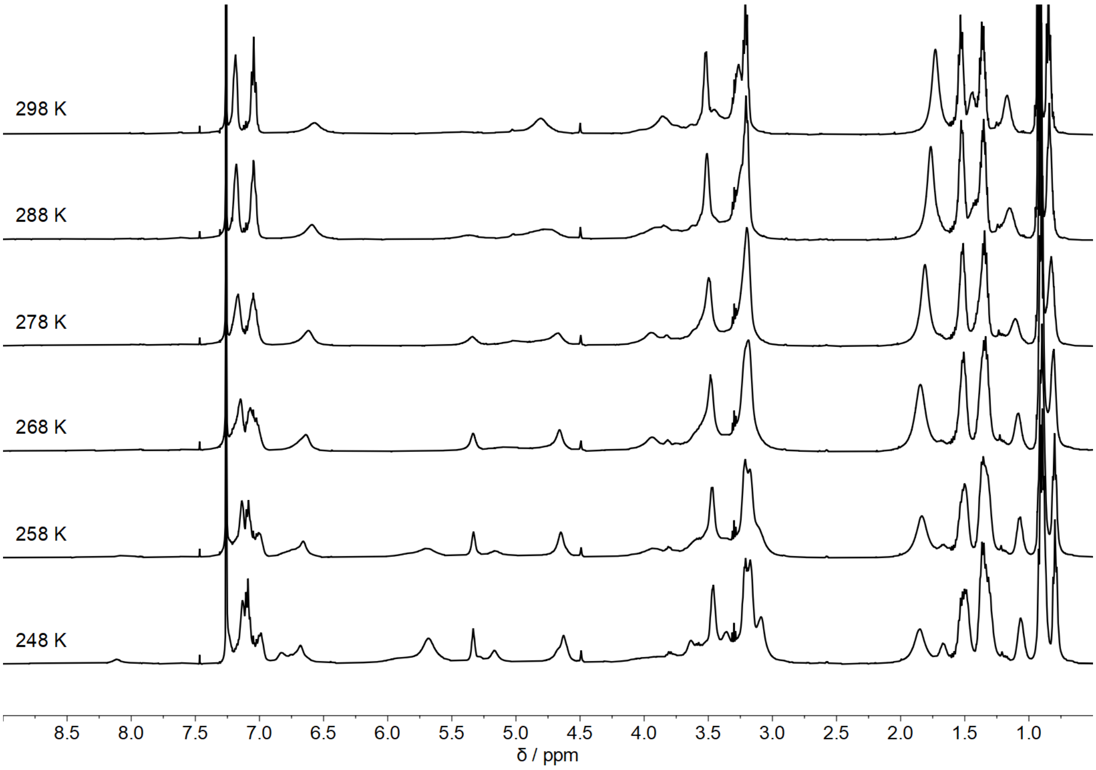

Figure S57. 500 MHz ^{1}H VT NMR of **26** (8 mM) in $CDCl_3$.

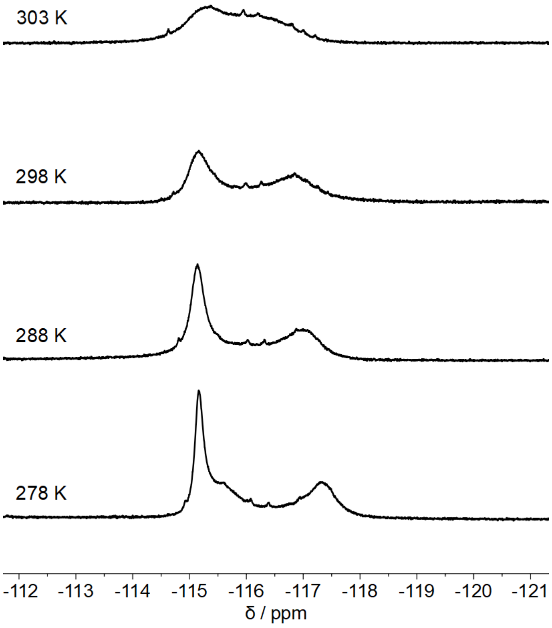

Figure S58. 565 MHz ^{19}F VT NMR of **26** (8 mM) in $CDCl_3$.

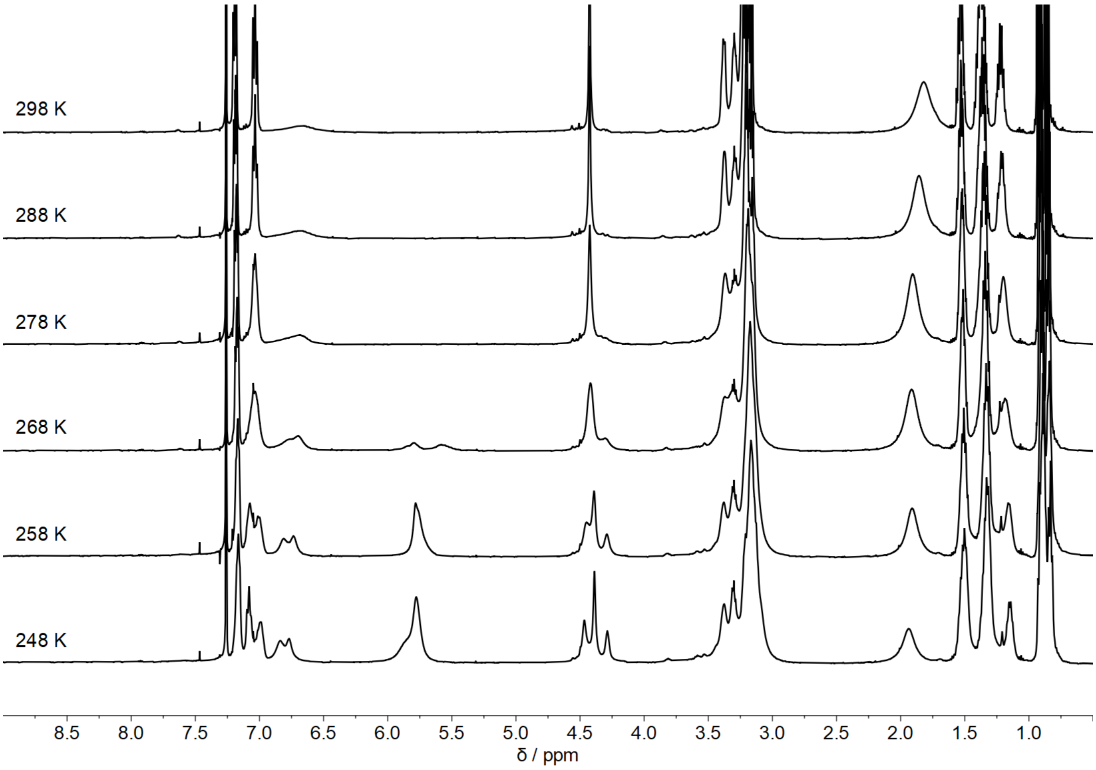

Figure S59. 500 MHz ^{1}H VT NMR of **27** (8 mM) in $CDCl_3$.

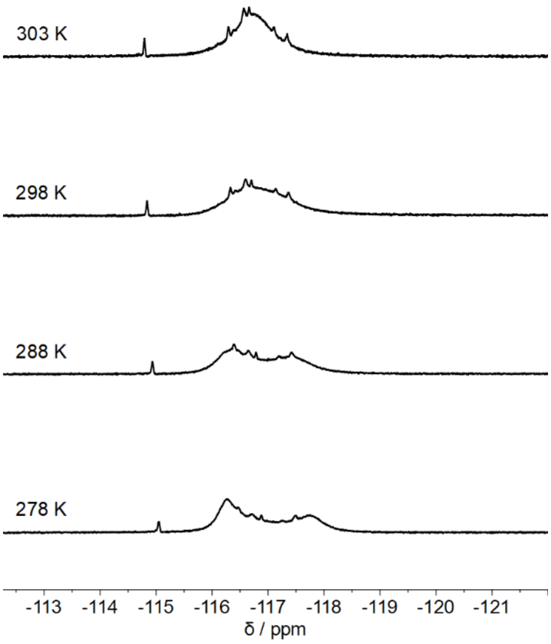

Figure S60. 565 MHz ^{19}F VT NMR of **27** (8 mM) in $CDCl_3$.

Foldamers **26** and **27** were successfully incorporated into the double lipid bilayer of small unilamellar vesicles (Figure S61), unlike **5**, which precipitated. However, the NMR signals in both the ^{1}H and ^{19}F NMR spectra of these membrane-incorporated foldamers at 298 K showed a significant increase in linewidth.

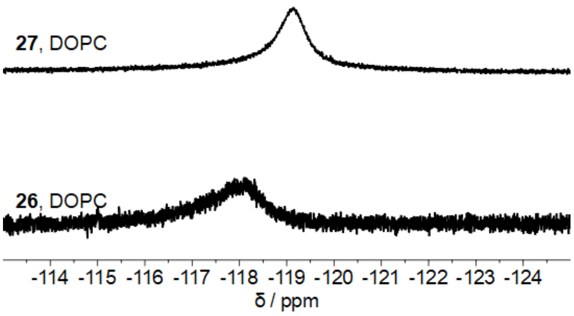

Figure S61. 565 MHz ^{19}F NMR spectra at 298 K of foldamers **27** (0.5 mM) or **26** (0.5 mM) embedded in vesicles (DOPC, 10 mM) suspended in MOPS (20 mM, pH 7.4), NaCl (100 mM), KF (0.5 mM) in D_2O.

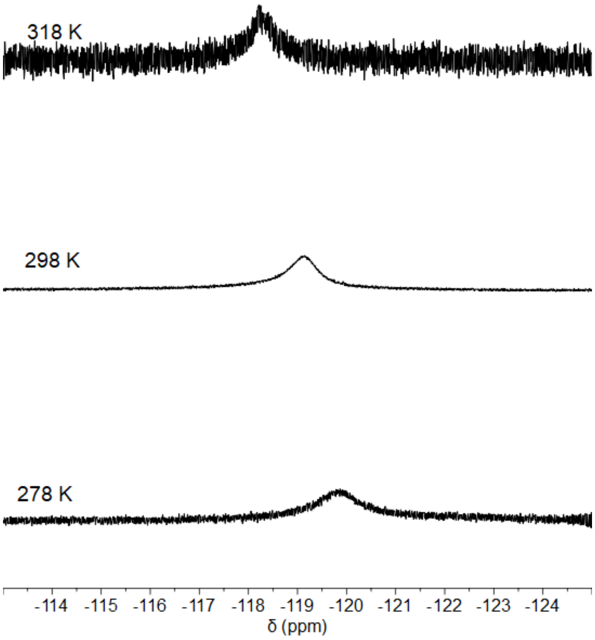

Figure S62. 565 MHz ^{19}F VT NMR of **27** (0.5 mM) embedded in the membrane of vesicles (DOPC, 10 mM) in MOPS (20 mM, pH 7.4), NaCl (100 mM), KF (0.5 mM).

To expand the approach reported in the manuscript to the study of the dynamic conformation of molecules in membrane-mimetic environments, we also incorporated into micelles the alternative foldamers **24-27** that we had synthesised to validate the choice of fluorine probe, side-chains and thiourea moieties. Compound **24** (Figure S63) exhibited decoalesced peaks for *H*2 and *H*2' and a broad peak for *H*3,3' in the ^{1}H NMR at 288 K that coalesced into two equally integrating peaks at 318 K, whilst two peaks starting to decoalesce at 288 K with a chemical shift difference of 0.1 ppm become one sharp peak at 318 K in the ^{19}F NMR. Thus the conformers of **24** remain in slow exchange, but the para-F probe of **3** is more sensitive to the hydrogen-bond directionalities in

micelles at this temperature than the *para*-CF_3 probe of **24**. Substituting the *n*-butyl side-chains of **3** with phenyl groups in **25** yielded two decoalesced and equally integrating peaks in the ^{19}F NMR spectra for these compounds incorporated in micelles, although the aromatic region in the ^{1}H NMR spectrum of **25** is complicated by the signals of the phenyl side-chains (Figure S64). Both pentamers **26** (Figure S65) and **27** (Figure S66) showed two peaks for *H*2,2' and *H*3,3' in the ^{1}H NMR spectra at all temperatures, but the ^{19}F NMR spectra at 288 K showed two unequally integrating peaks suggesting the population of at least two conformers.

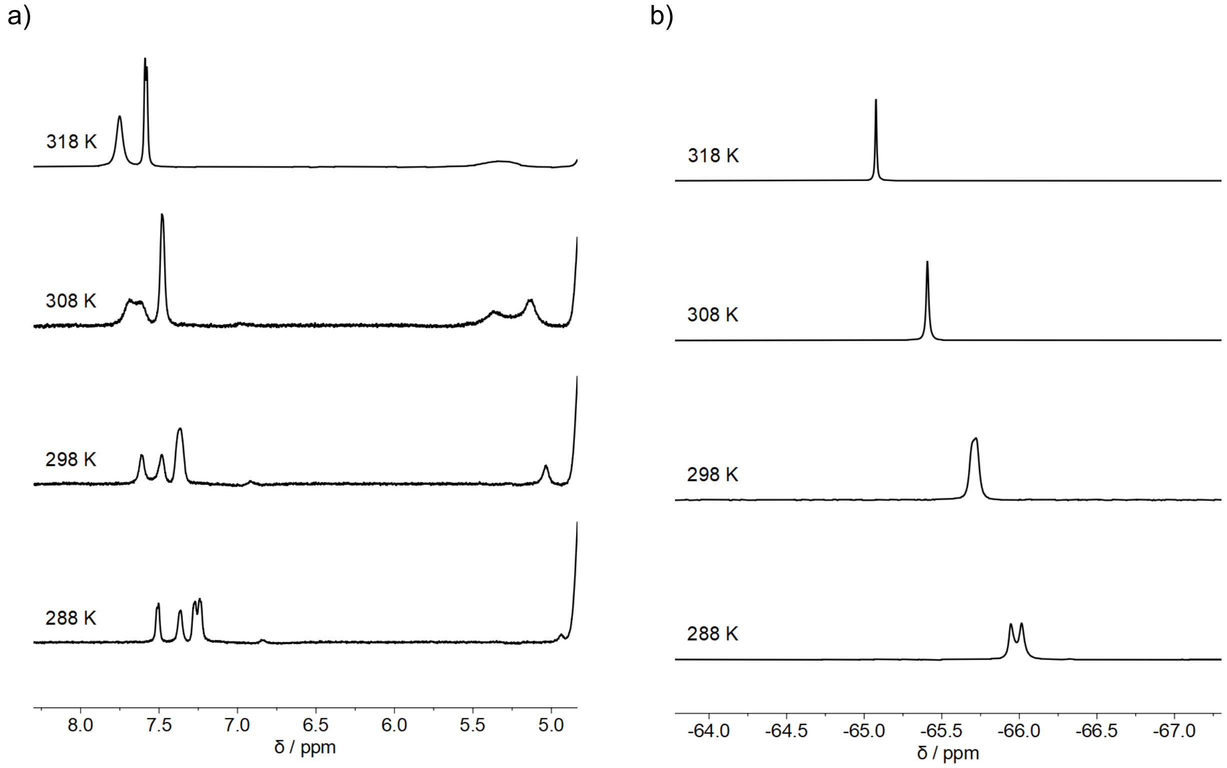

Figure S63. (a) 600 MHz ^{1}H (with presaturation[6] for the suppression of the water signal) and (b) 565 MHz ^{19}F VT NMR of **24** (1 mM) embedded in micelles (SDS, 200 mM) in MOPS (20 mM, pH 7.4), NaCl (100 mM), KF (0.05 mM).

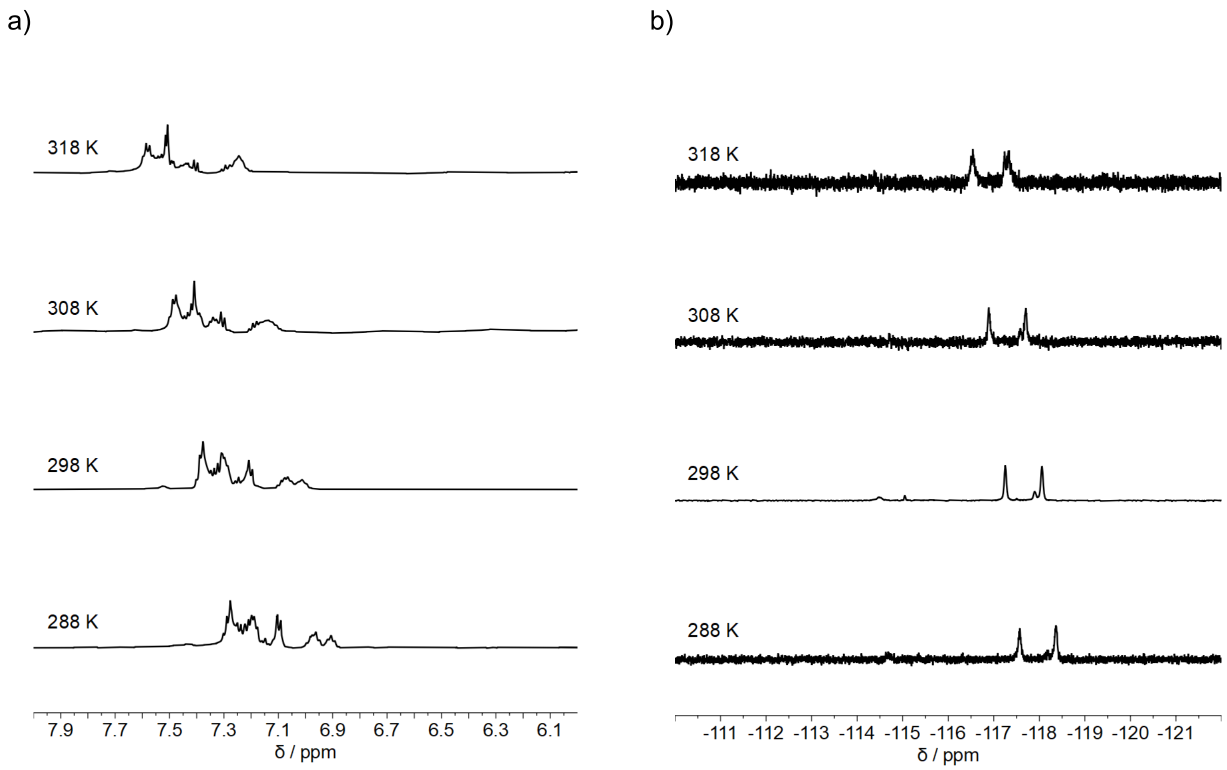

Figure S64. (a) 600 MHz ^{1}H (with presaturation[6] for the suppression of the water signal) and (b) 565 MHz ^{19}F VT NMR of **25** (1 mM) embedded in micelles (SDS, 200 mM) in MOPS (20 mM, pH 7.4), NaCl (100 mM), KF (0.05 mM).

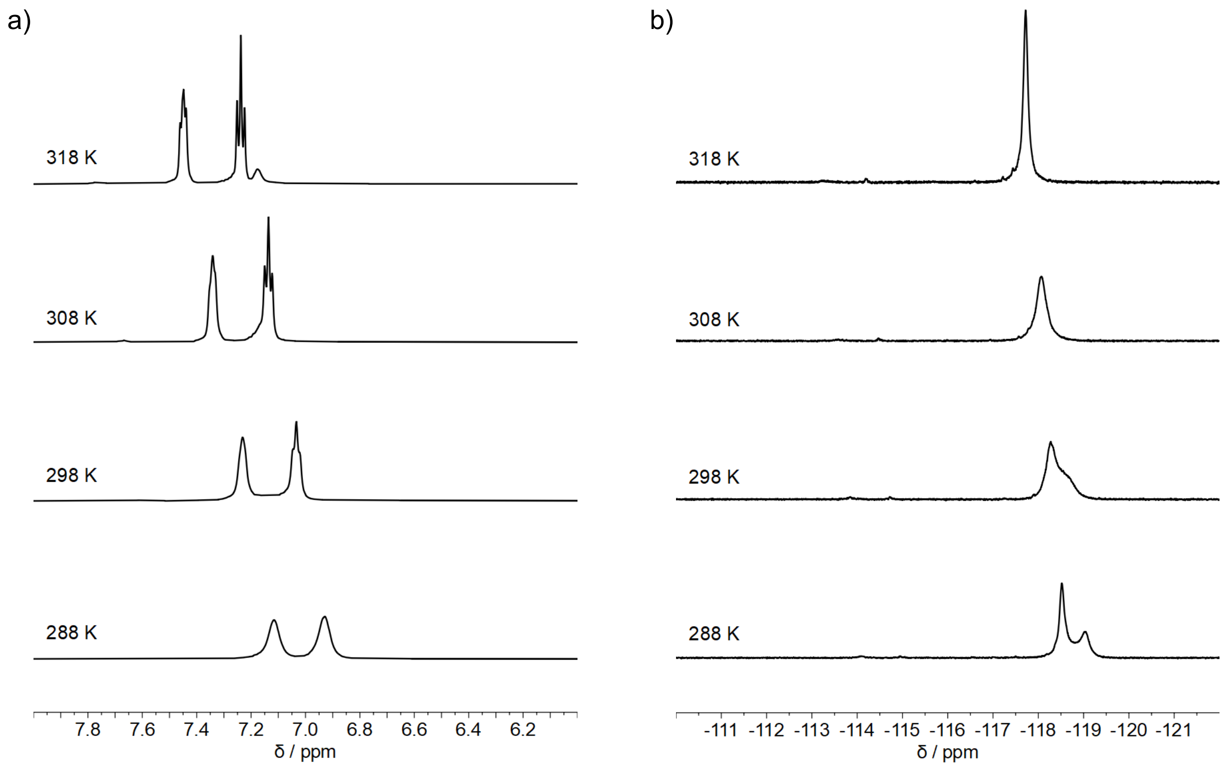

Figure S65. (a) 600 MHz ^{1}H (with presaturation[6] for the suppression of the water signal) and (b) 565 MHz ^{19}F VT NMR of **26** (1 mM) embedded in micelles (SDS, 200 mM) in MOPS (20 mM, pH 7.4), NaCl (100 mM), KF (0.05 mM).

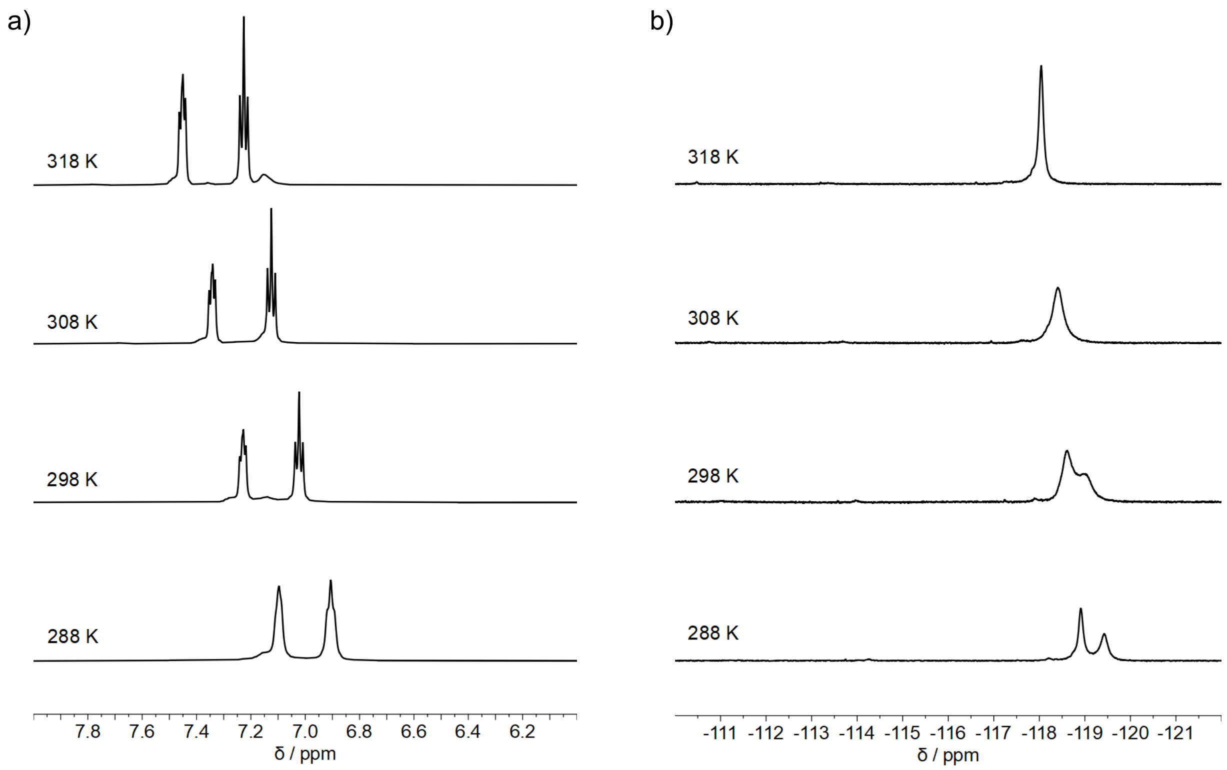

Figure S66. (a) 600 MHz ^{1}H (with presaturation[6] for the suppression of the water signal) and (b) 565 MHz ^{19}F VT NMR of **27** (1 mM) embedded in micelles (SDS, 200 mM) in MOPS (20 mM, pH 7.4), NaCl (100 mM), KF (0.05 mM).

Finally, for both pentamers **26** and **27** the ^{19}F NMR spectra show only one peak (Figures S67-S70). In contrast, in solution there are two broad peaks for **26** and a very broad signal for **27**, and in micelles they both show one peak with a shoulder at lower chemical shift. Thus, in foldamers containing hydrogen-bonded ureas (rather than thioureas) the rotation around the C-N bond is too fast to reveal different conformers at 298 K in either micelles or bicelles.

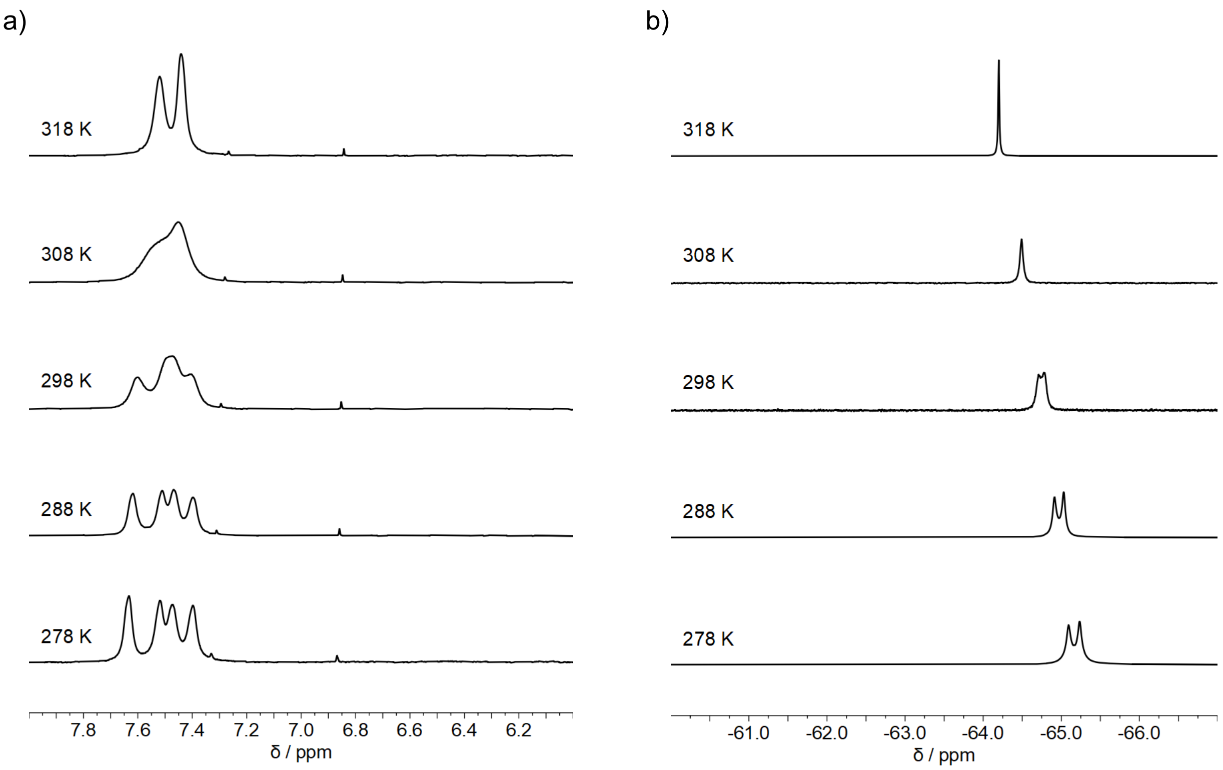

Figure S67. (a) 600 MHz ^{1}H (with presaturation[6] for the suppression of the water signal) and (b) 565 MHz ^{19}F VT NMR of **24** (1 mM) embedded in bicelles (DLPC:DHPC, 300 mM, q = 0.5) in MOPS (20 mM, pH 7.4), NaCl (100 mM), KF (0.05 mM).

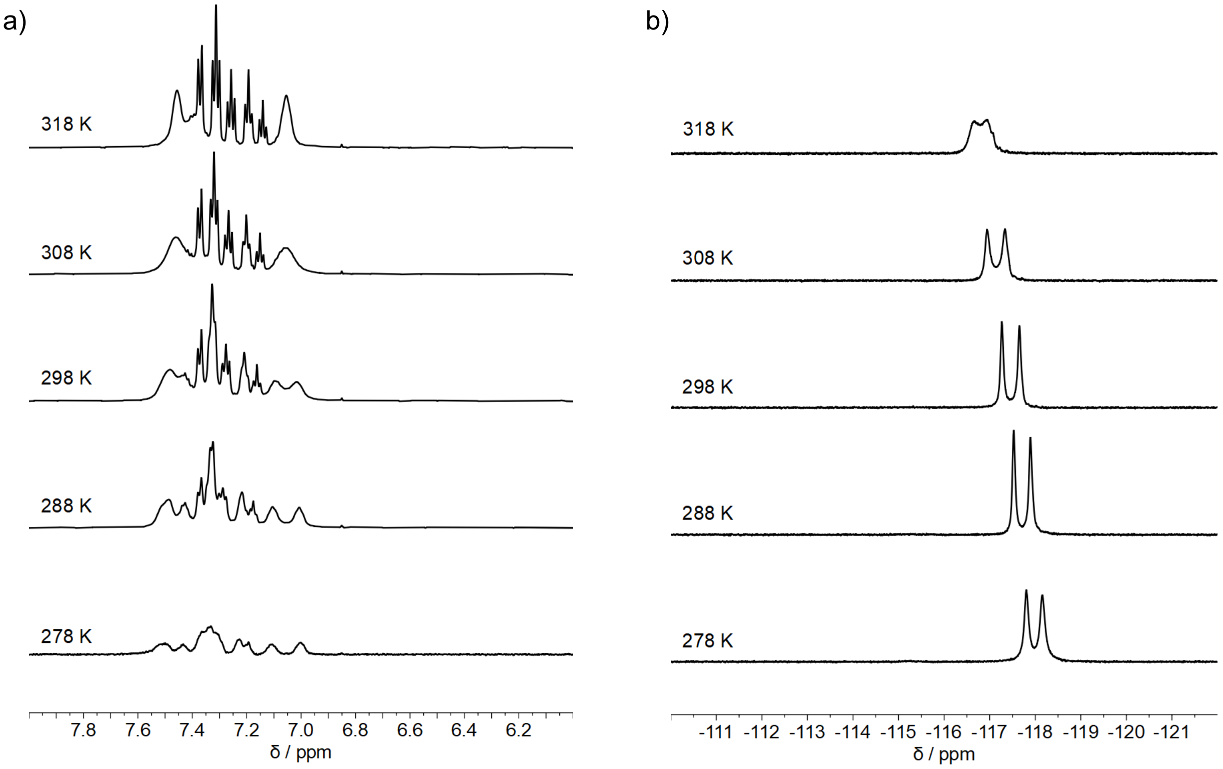

Figure S68. (a) 600 MHz ^{1}H (with presaturation[6] for the suppression of the water signal) and (b) 565 MHz ^{19}F VT NMR of **25** (1 mM) embedded in bicelles (DLPC:DHPC, 300 mM, q = 0.5) in MOPS (20 mM, pH 7.4), NaCl (100 mM), KF (0.05 mM).

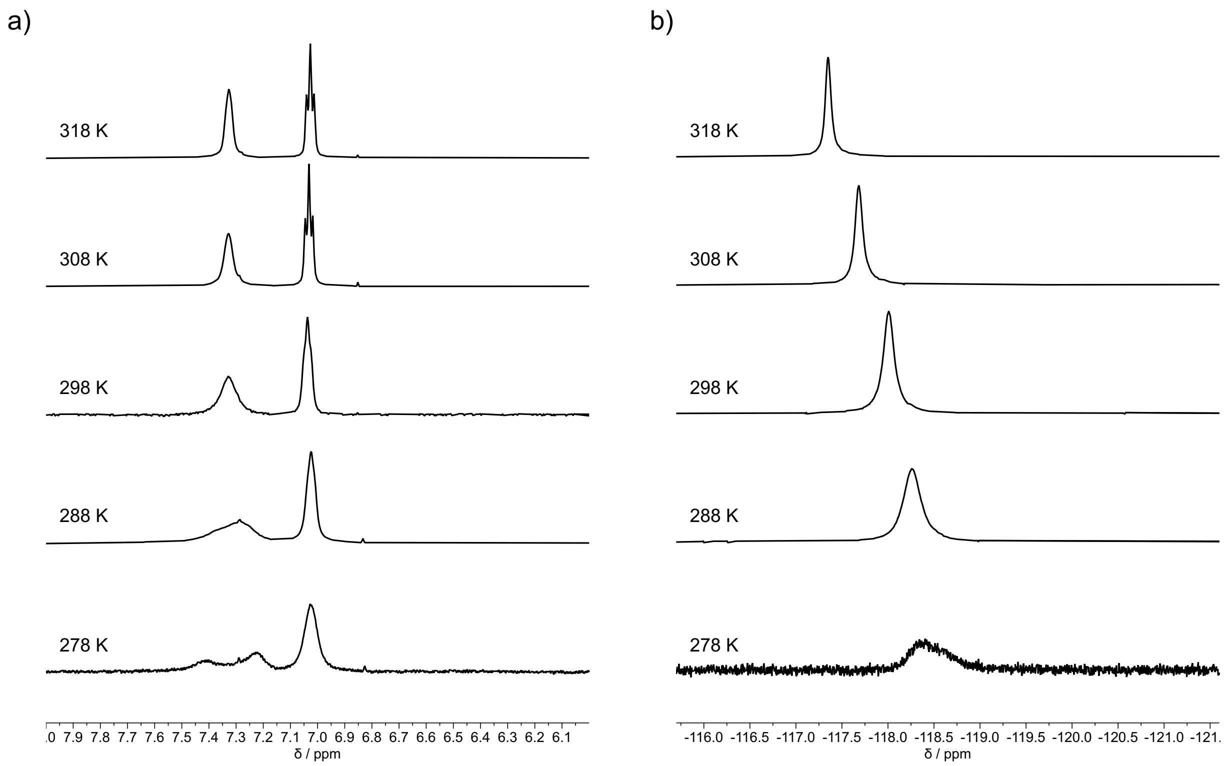

Figure S69. (a) 600 MHz ^{1}H (with presaturation[6] for the suppression of the water signal) and (b) 565 MHz ^{19}F VT NMR of **26** (1 mM) embedded in bicelles (DLPC:DHPC, 300 mM, q = 0.5) in MOPS (20 mM, pH 7.4), NaCl (100 mM), KF (0.05 mM).

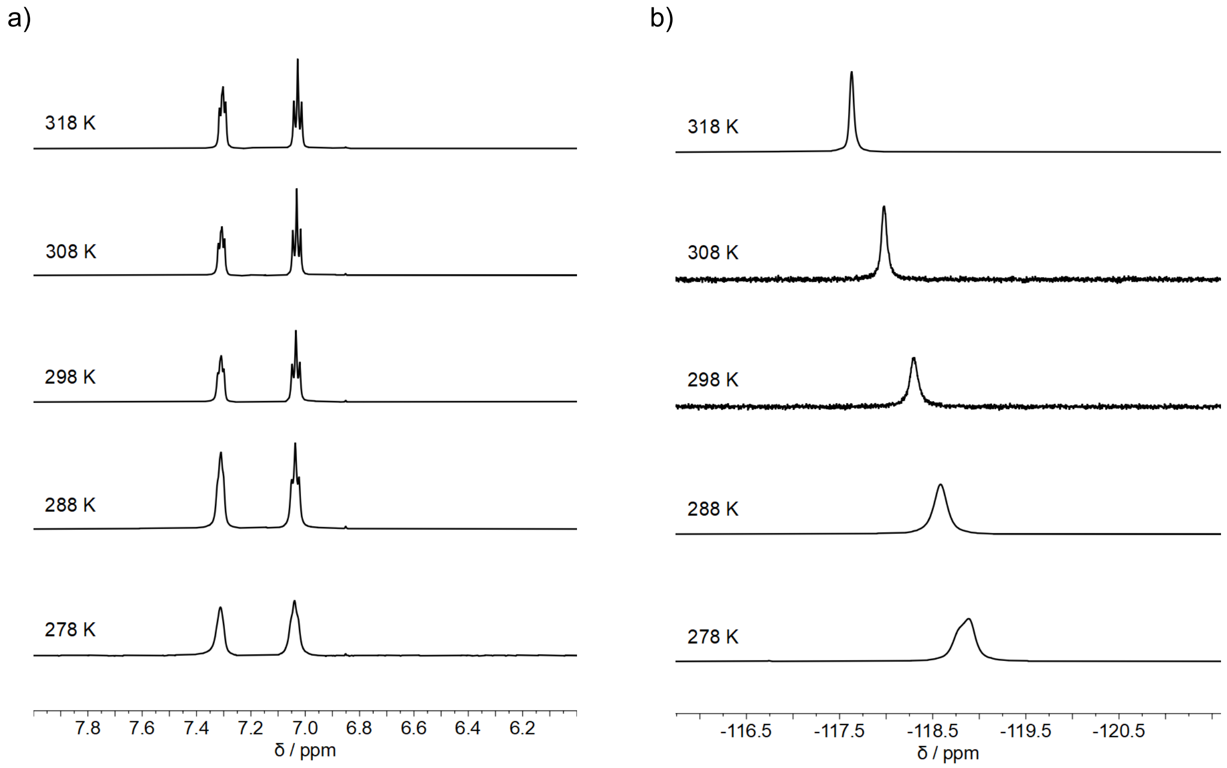

Figure S70. (a) 600 MHz ^{1}H (with presaturation[6] for the suppression of the water signal) and (b) 565 MHz ^{19}F VT NMR of **27** (1 mM) embedded in bicelles (DLPC:DHPC, 300 mM, q = 0.5) in MOPS (20 mM, pH 7.4), NaCl (100 mM), KF (0.05 mM).

S5 Computational Study

Molecular mechanics calculations were done using Schrödinger's Maestro software (2024-3 edition), with $CHCl_3$ as the solvent and OPLS2005 as the force field (charges assigned from the force field, the cut-off was none so that all non-bonded interactions are considered). The minimization method that was used was PRCG (Polar-Ribier Conjugate Gradient)[8] with a maximum iterations number of 10000, the convergence criterion was a gradient with a convergence threshold of 0.01 kJ mol^{-1} $Å^{-1}$. For each conformational search mixed torsional/low mode sampling was used as the method, the maximum number of steps was 10000, the number of structures saved for each search was 50 and the energy window for saving structures was 50.0 kJ mol^{-1}.

In the computed fifty lowest energy structures for compound **4**, the 'global' and 'broken' conformers **4a**, **4b** and **4c** were identified (see Figure 7 for the labelling and Figure S71). However, even by surveying the one hundred lowest energy structures, **4d** and **4e** could not be found.

a) b) c)

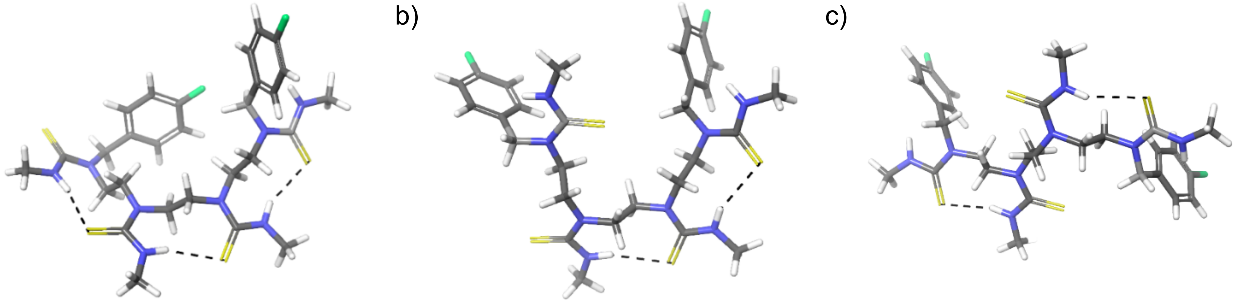

4a 17.028 kJ mol^{-1} | **4b** 19.124 kJ mol^{-1} | **4c** 24.915 kJ mol^{-1}

Figure S71. Conformations of **4** computed with force field OPLS2005 in $CHCl_3$: (a) **4a**, (b) **4b** and (c) **4c** with their corresponding computed energies.

S6 References

1. G. R. Fulmer, A. J. Miller, N. H. Sherden, H. E. Gottlieb, A. Mudelman, B. M. Stoltz, J. E. Bercaw, K. I. Goldberg *Organometallics* 2010, 29 (9), 2176-2179.
2. H. Song, Y. Xiao, Z. Zhang, W. Xiong, R. Wang, L. Guo, T. Zhou, J. Org. Chem. 2022, 87, 790-800
3. D.-Y. Cui, Y. Yang, M.-M. Bai, J.-X. Han, C.-C. Wang, H.-T. Kong, B.-Y. Shen, D.-C. Yan, C.-L. Xiao, Y.-S. Liu, E. Zhang, Bioorg. Chem. 2020, 101, 103965
4. R. C. MacDonald, R. I. MacDonald, B. P. Menco, K. Takeshita, N. K. Subbarao, L. R. Hu *Biochim. Biophys. Acta* 1991, **1061**, 297-303.
5. Dos Santos Morais, R.; Delalande, O.; Pérez, J.; Mouret, L.; Bondon, A.; Martel, A.; Appavou, M.-S.; Le Rumeur, E.; Hubert, J.-F.; Combet, S. Contrast-Matched Isotropic Bicelles: A Versatile Tool to Specifically Probe the Solution Structure of Peripheral Membrane Proteins using SANS. *Langmuir*, **2017**, *33* (26), 6572-6580.
6. Hoult, D. I. *J. Magn. Reson.* **1976**, *21* (2), 337-347.
7. J. Sandström, Dynamic NMR Spectroscopy, 1982, Academic Press Inc. (London) LTD., pp. 93-100.
8. E. Polak, G. Ribiere Revue Française d'Informatique et de Recherche Opérationelle. Série rouge, 1969, 16, 35-43.
